# Unraveling Interactions of the Necrotrophic Fungal Species *Botrytis cinerea* With 1-Methylcyclopropene or Ozone-Treated Apple Fruit Using Proteomic Analysis

**DOI:** 10.3389/fpls.2021.644255

**Published:** 2021-03-10

**Authors:** Stefanos Testempasis, Georgia Tanou, Ioannis Minas, Martina Samiotaki, Athanassios Molassiotis, Georgios Karaoglanidis

**Affiliations:** ^1^Laboratory of Plant Pathology, Faculty of Agriculture, Forestry and Natural Environment, Aristotle University, Thessaloniki, Greece; ^2^Institute of Soil Science and Water Resources, ELGO-Demeter, Thessaloniki, Greece; ^3^Laboratory of Pomology, Department of Horticulture and Landscape Architecture, Colorado State University, Colorado, CO, United States; ^4^Biomedical Sciences Research Center “Alexander Fleming”, Athens, Greece; ^5^Laboratory of Pomology, Faculty of Agriculture, Forestry and Natural Environment, Aristotle University, Thessaloniki, Greece

**Keywords:** apple proteome, gaseous O_3_, Granny Smith, gray mold, postharvest treatments, 1-methylcyclopropene

## Abstract

Gray mold caused by the necrotrophic fungus *Botrytis cinerea* is one of the major postharvest diseases of apple fruit. The exogenous application of 1-methylcyclopropene (1-MCP) and gaseous ozone (O _3_) is commonly used to ensure postharvest fruit quality. However, the effect of these treatments on the susceptibility of apple fruit to postharvest pathogens remains largely unknown. Herein, the effect of O _3_ and 1-MCP treatments on the development of gray mold on apple fruit (cv. “Granny Smith”) was investigated. Artificially inoculated apple fruits, treated or not with 1-MCP, were subjected for 2 months to cold storage [0°C, relative humidity (RH) 95%] either in an O_3_-enriched atmosphere or in a conventional cold chamber. Minor differences between 1-MCP-treated and control fruits were found in terms of disease expression; however, exposure to ozone resulted in a decrease of disease severity by more than 50% compared with 1-MCP-treated and untreated fruits. Proteomic analysis was conducted to determine proteome changes in the mesocarp tissue of control and 1-MCP- or O_3_-treated fruits in the absence or in the presence of inoculation with *B. cinerea*. In the non-inoculated fruits, 26 proteins were affected by 1-MCP, while 51 proteins were altered by ozone. Dynamic changes in fruit proteome were also observed in response to *B. cinerea.* In O_3_-treated fruits, a significant number of disease/defense-related proteins were increased in comparison with control fruit. Among these proteins, higher accumulation levels were observed for allergen, major allergen, ACC oxidase, putative NBS-LRR disease resistance protein, major latex protein (MLP)-like protein, or 2-Cys peroxiredoxin. In contrast, most of these proteins were down-accumulated in 1-MCP-treated fruits that were challenged with *B. cinerea*. These results suggest that ozone exposure may contribute to the reduction of gray mold in apple fruits, while 1-MCP was not effective in affecting this disease. This is the first study deciphering differential regulations of apple fruit proteome upon *B. cinerea* infection and postharvest storage treatments, underlying aspects of host response related to the gray mold disease.

## Introduction

Apple (*Malus domestica* Borkh) is one of the most popular fruit crops cultivated throughout the world, with more than 80,000,000 tonnes of annual production (FAO, 2018). Apple fruits are characterized by their long storage capacity that can be extended by exogenous application of compounds interfering with ethylene biosynthesis or ethylene activity that delay fruit ripening (Karagiannis et al., 2018). However, during the long period of storage, apples become susceptible to various biotic (fungi and bacteria deterioration) and abiotic factors (physiological disorders), which lead to great economic losses. Among the biotic disorders, fungal pathogens play a predominant role in fruit postharvest decay. It is estimated that more than 90 fungal species may be agents of postharvest diseases on apple fruits, and some of them can cause devastating losses that can reach up to 25% (Jijakli and Lepoivre, 2004).

Gray mold caused by the necrotrophic pathogen *Botrytis cinerea* is a major postharvest disease of apple fruits throughout the world (Konstantinou et al., 2011; Nybom et al., 2020). Infections by the pathogen occur mostly through wounds caused during harvest or during postharvest processes in the packing house, while *B. cinerea* can also infect apple fruits during bloom or just after fruit setting through the open calyx of the fruits. However, the disease symptoms appear on the infected fruits during storage. Symptoms consist in the appearance of light tan to dark brown lesions that are irregular in shape without well-defined margins between healthy and decayed tissues (Konstantinou et al., 2011; Nybom et al., 2020).

As is typical, necrotrophic pathogen *B. cinerea* during the infection process modulates the host environment by lowering the pH of the host tissues, a condition conducive for fungal development and increase in virulence (Alkan et al., 2013; Rascle et al., 2018). Host tissue acidification is mediated through the production of gluconic, fumaric, and citric acids and is regulated by *PacC*, a conserved transcription factor that is involved in *pH* regulation in several phytopathogenic fungi including *B. cinerea* (Rascle et al., 2018). The lowering of *pH* within host tissues enhances the activity of several cell wall-degrading enzymes, such as polygalacturonases, employed by necrotrophic pathogens to destroy pectin and the primary cell walls of the host, to cause cell death or to alter the integrity of the host cells (Blanco-Ulate et al., 2014).

Despite that immunity of apple cultivars to gray mold does not exist, there is a variability in the level of their susceptibility to the disease, as has been shown by several previous studies assessing cultivars’ susceptibility level with either natural infections or artificial inoculations (Spotts et al., 1999; Konstantinou et al., 2011). These differences in the level of cultivar susceptibility are associated with fruit ripening-related characteristics, such as the rate of softening as well as with the chemical constituents of the peel and the flesh tissues, including polyphenol biosynthesis and pathogenesis-related (PR) protein activity (Ma et al., 2018). Particularly, the activation of ethylene biosynthesis in the harvested fruit has been found to play a critical role in the induction of resistance responses that are associated with PR proteins, polyphenolic compounds, or lignin biosynthesis (Akagi et al., 2011).

Apple, as a climacteric fruit, is characterized by a burst of respiration and ethylene production during its long storage life, which subsequently lead to a rapid fruit ripening accompanied by an undesirable fruit softening (Karagiannis et al., 2020). The 1-methylcyclopropene (1-MCP), an inhibitor of ethylene action, has been widely used in horticultural crops, particularly in apple fruits, to delay ripening, especially following long-term storage (Li et al., 2016; Minas et al., 2018). Also, 1-MCP applications have proven to be beneficial for the control of many physiological disorders, such as apple superficial scald (Karagiannis et al., 2018). Moreover, the effects of 1-MCP treatments on decay development are controversial and differ among the several fruit commodities tested (Li et al., 2016). In some cases, 1-MCP applications increased the susceptibility of the treated fruits, while there are some examples in which 1-MCP reduced the growth of postharvest pathogens (Mir et al., 2001; Díaz et al., 2002; Janisiewicz et al., 2003; Marcos et al., 2005). Meanwhile, ozone treatments can also delay climacteric fruit ripening by directly oxidizing ethylene, while, in addition, it can reduce the residues of various mycotoxins and fungicides (Karaca and Velioglu, 2007; Minas et al., 2018). However, apple fruit exposure to a high concentration of ozone may stimulate oxidative damage and physiological disorders such as superficial scald (Yaseen et al., 2015; Lv et al., 2019). Ozone has been tested against several pathogens on several horticultural fruits, with contradictory results (Palou et al., 2002; Karaca and Velioglu, 2007).

Proteomic analysis has been used to investigate complex biological processes such as the responses of fruit tissues to pathogens (Papavasileiou et al., 2020). With the use of proteomics, the response of *B. cinerea* to plant-based elicitors and hormones (Liñeiro et al., 2016) and the *in vitro* secretome of *B. cinerea* related to pathogenesis (González-Fernández et al., 2015) have been investigated. In apple fruits, proteomic studies have focused mostly on the investigation of protein changes during the ripening process (Zheng et al., 2013; Shi et al., 2014), while the available information on the effect of postharvest treatments and pathogen’s presence on the modulation of apple proteome is limited (Buron-Moles et al., 2015).

In this work, we characterize the effect of 1-MCP and gaseous O_3_ on the development of gray mold of apple (cv. Granny Smith) fruits. To improve our fundamental understanding of *B. cinerea* development and its response to 1-MCP and gaseous O_3_ treatments, we conducted proteomic analysis to quantitatively investigate protein profiles of apple mesocarp of control and 1-MCP- or O_3_-treated fruits following inoculation with *B. cinerea* to identify proteins potentially involved in fruit responses to pathogen attack.

## Materials and Methods

### Fruit Material

Apple fruits (cv. Granny Smith) used in the experiments were collected from an orchard located in the region of Imathia (northern Greece) at the commercial harvest stage. The fruits selected for the experiments originated from trees that did not receive any fungicide application during the last 2 months before harvest. The selected fruits were healthy, without any visual defect, and they had an average weight of 150 g. The fruits immediately after harvest were transferred to the fruit storage facilities of AUTH and stored at a conventional chamber at 1°C and 95% relative humidity (RH).

The harvested fruits were divided into three groups, one for each treatment (untreated control, 1-MCP-treated, and O_3_-treated fruits; see below), and placed on single-layer wooden trays with plastic vents. For each treatment, three replicates of 10 fruits were used for artificial inoculations, as well as for the ripening measurements after 60 days of storage.

### Treatments With 1-Methylcyclopropene and O_3_

The application of 1-MCP (SmartFresh^TM^, AgroFresh Inc., Rohm and Haas, Spring House, PA, United States) was conducted 24 h after harvest. 1-MCP was applied at the commercially recommended dose of 500 μl L^–1^ for 24 h at 1°C. The application of O_3_ was initiated after the artificial inoculation of the fruits and was continuous during the entire cold storage period. For the production and monitoring of ozone concentration, an oxygen generator (model SEP-100, Anseros Klaus Non-nenmacher GmbH, Tubingen, Germany), a corona discharge ozone generator (model COM-AD-04, Anseros Klaus Non-nenmacher GmbH, Tubingen, Germany), and an ozone analyzer (model MP-6060, Anseros Klaus Non-nenmacher GmbH, Tubingen, Germany) were used.

### Ripening Parameters of Apple Fruits

Ripening parameters, including firmness, titratable acidity (TA), and total soluble solids, of apple fruits were measured on the harvest day and after 60 days storage period, as previously outlined (Karagiannis et al., 2020). On the harvest day, the ripening status of apple fruits was performed using 30 replicate fruits (10 × 3 fruits in three independent replications). Similarly, after the end of the storage period, ripening parameters were conducted on 30 replicate fruits per treatment (control, 1-MCP-treated, and O_3_-treated fruits; 10 × 3 fruits in three independent replications). Fruit firmness was measured in two opposite sites of each fruit after peeling of skin using a texture analyzer (model 53205, T.R. Turoni Srl, Forli, Italy) with a 12 mm probe. Soluble solids content (SSC) measurement was performed in juice using a refractometer (Atago PR-1, Atago Co., Ltd., Tokyo, Japan) and TA by acid–base titration of maleic acid (Karagiannis et al., 2020).

### Fungal Strain and Artificial Inoculations

A *Botrytis cinerea* isolate (Bc Z08) that had been obtained from infected apple fruits and identified for the requirements of monitoring studies conducted by our group was used in the study. Inoculum was produced by cultivating the isolate on Potato Dextrose Agar (PDA) for 7 days at 25°C under continuous light. Conidia were suspended from the petri dishes using a scalpel, and inoculum suspensions were prepared in sterile distilled water supplemented with 0.1% (v/v) Tween 80 (PanReac Applichem, Germany). Inoculum suspensions density was adjusted at 4 × 10^5^ conidia ml^–1^, using a hemocytometer counting chamber (Neubauer, Heinz Herenz Hamburg, Germany).

On all the fruit sets, artificial inoculations were conducted 48 h after harvest (24 h after 1-MCP application on 1-MCP-treated fruit). Before inoculation, the fruits had been surface-sterilized by immersion in a 70% (v/v) ethanol solution for 2 min. Then, fruits were wounded with a sterile needle (5 mm) and inoculated with 45 μl of conidial suspension. Control fruits were similarly wounded and inoculated with 45 μl of distilled sterile water.

### Storage Conditions and Disease Incidence/Severity Measurements

Following inoculation, the fruits were incubated for 3 h at 20°C to allow fungal spores to germinate and then transferred for cold storage. Control fruits and fruits that had been treated with 1-MCP were stored in a conventional cold chamber (1°C and RH 95%), while the rest of fruits were stored in an ozone-enriched atmosphere chamber (O_3_ treatment) (0.3 μl L^–1^, 1°C, RH 95%). Ozone generation was performed as described above.

After 60 days of cold storage, disease incidence (%) was measured by counting the number of the fruits with decay symptoms around the inoculation point, and the disease severity was determined by measuring the lesion diameter (cm) around the inoculation point.

### Proteomic Analysis

After 60 days of cold storage, 20–30 g of skinless flesh of the mesocarp were carefully removed from each fruit antipodal to the inoculation point, to avoid any rotten tissue and the potential presence of fungal mycelia. Samples were cut into small pieces, placed in polyethylene bags (10 fruits per bag), frozen instantly in liquid nitrogen, and stored at -80°C until further analysis. In total, 30 fruits (3 × 10 fruits in each replicate) were utilized for the analysis. Apple protein extraction was performed with 5 g of mesocarp tissue based on a phenol extraction protocol (Karagiannis et al., 2020). Two-dimensional electrophoretic separation followed Minas et al. (2016) described procedure using a BIO-RAD system. For imaging statistical analysis, silver nitrate staining was followed by two-dimensional gel electrophoresis (2-DE) scanning with a Bio-Rad GS-800 Calibrated Densitometer equipped with PDQuest Advanced 2-DE Gel Analysis Software, whereas for mass spectrum analysis, a compatible modified silver nitrate staining protocol using Silver Stain Plus kit (Bio-Rad) was performed according to Tanou et al. (2015). Statistics were done by one-way analysis of variance for significance level (*P* ≤ 0.05). Student’s *t*-test (significance level 95%) was applied to compare means. The statistically significant differences were further combined by a quantitative 2.0-fold change of spot volume. At least three biological replicates were performed for each fruit treatment.

Selected of interest spots underwent tryptic in-gel digestion and peptide fragments analyzed by matrix-assisted laser desorption ionization MS (MALDI-MS) in a time-of-flight MS (TOF-MS) (Ultraflex II, Bruker Daltonics, Bremen, Germany) as detailed by Ainalidou et al. (2016). Peptide mixtures were injected in a MALDI-TOF mass spectrometer (Autoflex-Speed, Bruker Daltonics). Raw files were searched against the Uniprot *Malus domestica* protein database using the (MASCOT Server v2.0). The mass error tolerance on the Mascot server was set to 25 ppm, methionine oxidation was considered as a variable modification, and cysteine carbamidomethylation was considered as a fixed modification. All information regarding peptide sequences, accession numbers, Mascot scores, and sequence coverage are provided in Supplementary Table S1B. For non-identified spots, peptide fragments were loaded on an LC-MS/MS using a LTQ Orbitrap XL Mass spectrometer (Thermo Fisher Scientific, Bremen, Germany) coupled online with a nanoLC Ultimate 3000 chromatography system (Dionex, Sunnyvale, CA) (Karagiannis et al., 2016). Raw files were searched against the ncbi *M. domestica* protein database using PD 1.4 and the SEQUEST HT search engine. Protein identification required minimal XCorr values of 2.0 and 2.5 for charge states of doubly and triply charged precursor ions, respectively (Supplementary Table S1A). Manual protein BLAST against current databases was performed where protein annotations were missing. Identifications are based on at least two peptides per protein. This work is Minimum Information About a Proteomics Experiment (MIAPE) compliant.

### Data Analysis

The Pearson chi-square test was used to compare disease incidence values on fruits of the three different treatments. Disease incidence percentage values were arcsine transformed for statistical analysis. Data on disease severity in terms of lesion diameter were subjected to one-way analysis of variance and the least significant differences (Duncan) at *P* = 0.05 were used for means comparison. Statistical analysis transacted using SPSS v21.0 (SPSS, Chicago, IL, United States). All the graphical presentation was created with GraphPad Prism (GraphPad Prism version 8.0.0 for Windows, GraphPad Software, San Diego, California United States)^1^.

## Results

### The Effect of 1-Methylcyclopropene and O_3_ Treatments on the Ripening Characteristic of Apple Fruit

**FIGURE 1:**
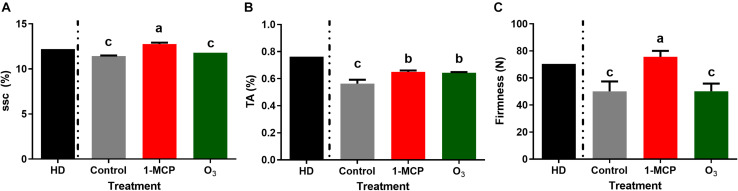
Physiological quality parameters: **(A)** SSC (soluble solids content;°Brix), **(B)** TA (titratable acidity;%), and **(C)** Firmness (fruit firmness; *N*) of “Granny Smith” apples following 1-methylcyclopropene (1-MCP) and O_3_ treatments after 60 days of cold storage in a conventional [1°C, relative humidity (RH) 95%] or in an O_3_-enriched cold chamber (0.3 μl L^–1^, 1°C, RH 95%). HD: measurements of quality parameter at harvest day. Letters on the bars indicate significant differences between the treatments, according to one-way ANOVA at *P* = 0.05.

Measurements of fruit qualitative parameters on the harvest day revealed that mean SSC, TA, and fruit firmness levels were of 12.2°Brix, 0.76%, and 70.5 N, respectively (Figure 1). In 1-MCP-treated fruits after 60 days of storage, most of the studied quality parameters remained at harvest day’s level. After 60 days of storage, SSC values were significantly higher (*P* < 0.05) in 1-MCP-treated fruits (12.8°Brix) compared with control (11.43°Brix) and O_3_-treated (11.8°Brix) fruits (Figure 1A). Similarly, fruit firmness was substantially higher (*P* < 0.05) in 1-MCP-treated fruits (75.6 N) compared with that of control (50.1 N) or O_3_-treated (50.2 N) fruits (Figure 1C). TA levels were substantially reduced in the three postharvest conditions compared with the initial ones determined on harvest day. Moreover, the reduction in TA was higher in untreated control fruits compared with that observed in 1-MCP- or O_3_-treated fruits (Figure 1B).

### The Effect of 1-Methylcyclopropene and O_3_ Treatments on Gray Mold Incidence and Severity

**FIGURE 2:**
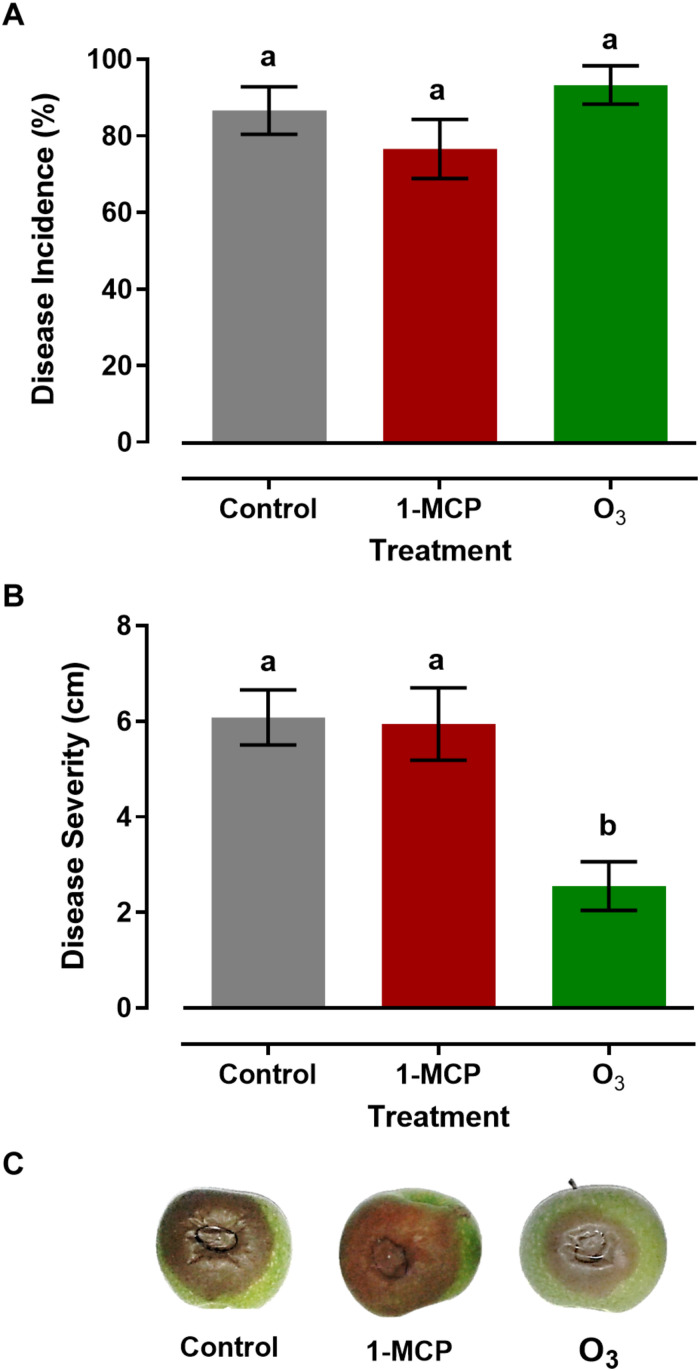
**(A)** Disease incidence (%; infected apples), **(B)** Severity (cm; lesion diameter), and **(C)** Symptoms of gray mold disease caused by *Botrytis cinerea* on artificially inoculated apple fruits (cv. “Granny Smith”) after 60 days of cold storage in a conventional [1°C, relative humidity (RH) 95%] or in an O_3_-enriched cold chamber (0.3 μl L^–1^, 1°C, RH 95%). Asterisks and letters on the bars indicate significant differences between disease incidence and severity values, according to a chi-square and one-way ANOVA at *P* = 0.05, respectively.

Measurements of gray mold incidence on apple fruit treated with 1-MCP or O_3_ showed that these treatments did not affect (*P* > 0.05) disease incidence compared with that on the control fruits. In detail, the rates of the disease incidence in response to control, 1-MCP, and O_3_ treatment were 86.6, 76.6, and 93.3%, respectively (Figure 2A). However, distinct differences were observed among the different treatments regarding disease severity. The lower (*P* < 0.05) lesion diameter was observed in O_3_-treated apple fruits with a mean value of 2.55 cm, while no difference (*P* > 0.05) was observed between 1-MCP-treated and control fruits, with mean lesion diameter values of 5.95 and 6.07 cm, respectively (Figures 2B,C). These results indicated that the O_3_ application significantly suppressed *Botrytis cinerea*, while 1-MCP treatment had no significant impact on the pathogen.

### Characterization of Apple Fruit Proteome Following Long-Term Storage

**FIGURE 3:**
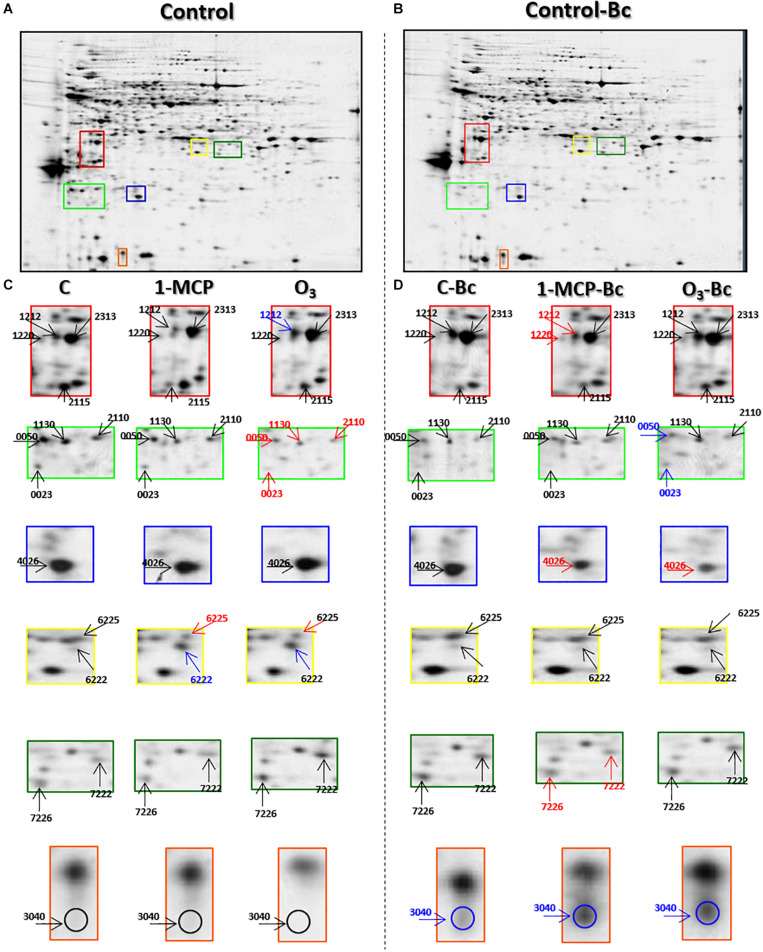
Representative silver-stained two-dimensional gel electrophoresis (2-DE) map of total proteins from **(A)** Untreated (control) and **(B)** Untreated inoculated (control-Bc) apple fruits (cv. “Granny Smith”). **(C,D)** Enlarged view panels of selected areas of control in relation to 1-methylcyclopropene (1-MCP)- and O_3_-treated apple fruits inoculated or not with the necrotrophic pathogen *Botrytis cinerea*. Blue, red, and black arrows demonstrate protein spots abundance increased, decreased, or remained unchanged, respectively, compared with the control. Enlarged in views of selected areas of full-length gels are provided as Supplementary Figure S1.

To better understand the mechanisms behind the observed resistance of apple fruits to *B. cinerea* of O_3_-treated fruits, 2-DE analysis was performed to identify protein changes between the postharvest treatments tested (Figures 3A,B). Among all treatments (inoculated or not), more than 500 protein spots were detected. At the same time, 98 of them exhibited significant differences in their abundance of either inoculated or not 1-MCP- and O_3_-treated apple fruits according to the Student *t*-test with 95% confidence interval, further validated by 2-fold change threshold (Figures 3C,D and Supplementary Table S3). According to the mass spectrum analysis, 138 proteins were identified, while 19 proteins were detected in more than one spot, and 10 proteins were not identified. In detail, these proteins were 2-methylene-furan-3-one-reductase (spot nos. 5,223 and 6,218), 5-methyltetrahydropteroyltriglutamate-homocysteine methyltransferase (spot nos. 7,720 and 7,728), ACC oxidase (ACO; spot nos. 1,132, 1,212, 1,220, 2,213, and 3,220), actin (spot nos. 23, 3,220, and 3,327), allergen (spot nos. 1,037 and 2,029), ATP-citrate synthase beta chain protein (spot nos. 8,606 and 9,603), beta galactosidase (spot nos. 3,719 and 9,708), CNP-60 (spot nos. 2,631 and 2,634), fructose-bisphosphate aldolase (spot nos. 6,222, 6,225, 7,222, and 9,227), glyceraldehyde-3-phosphate dehydrogenase (spot nos. 3,211, 9,313, and 9,314), L-3-cyanoalanine synthase (spot nos. 2,320, 3,322, and 3,324), major allergen (spot nos. 1,037, 3,040, and 4,016), NADP-dependent-D-sorbitol-6-phosphate dehydrogenase (spot nos. 5,627, 7,222, 7,226, 8,232, and 9,221), phosphoenolpyruvate carboxylase (spot nos. 6,834, 6,905, and 6,908), porin (spot no. 8,732, 9,117, 9,118), proteasome subunit alpha (spot nos. 4,105 and 8,123), serine/threonine protein phosphatase (spot nos. 109 and 1624), stem-specific protein (spot nos. 210 and 2,113), and V-ATPase (spot nos. 1,510, 1,511, and 2,727). Further information regarding the identified proteins and their functional categorization based on their gene ontology and literature is provided in Supplementary Tables S1A,B.

### Functional Classification of Proteins Affected by 1-Methylcyclopropene and O_3_ Treatments in Non-inoculated Fruit

**FIGURE 4:**
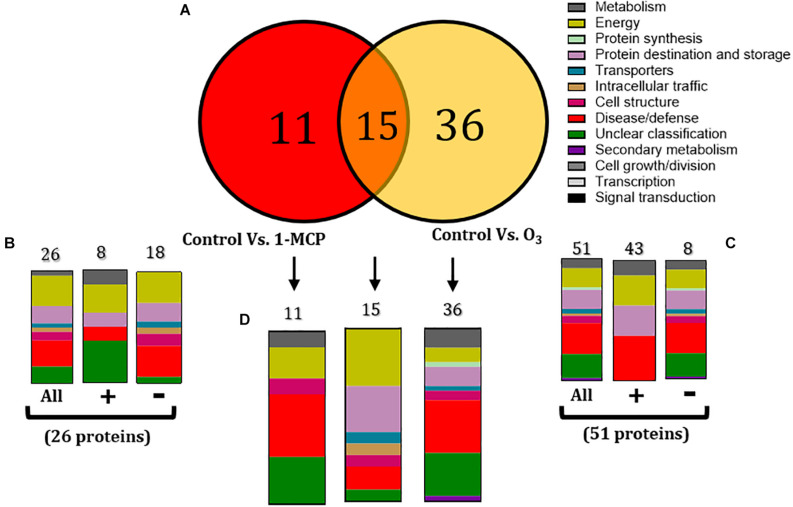
**(A)** Venn diagram demonstrating the common and distinct differentially expressed proteins in the 1-methylcyclopropene (1-MCP)- and O_3_-treated apple fruits (cv. “Granny Smith”) proteome after 60 days of cold storage [1°C, relative humidity (RH) 95%]. Graphical representation of functional classification of differentially expressed proteins in **(B)** Apple fruits treated with 1-MCP and incubated in conventional cold chamber, **(C)** Apple fruits incubated in an O_3_-enriched atmosphere chamber (0.3 μl L^– 1^, 1°C, RH 95%), and **(D)** Functional categorization of the unique and overlapping proteins presented in the Venn diagram **(A)**. Symbols (+) and (-) present the up- and downregulated proteins on each treatment.

To determine the apple proteins whose abundance changed (increased or decreased) by the 1-MCP and O_3_ applications compared with the untreated control fruits, proteins were grouped into two sets: (1) a set of 26 proteins that were changed in non-inoculated fruits by the application of 1-MCP (control vs. 1-MCP) (Figure 4B), and (2) a set of 51 proteins that were altered in non-inoculated fruits by the O_3_ application (control vs. O_3_) (Figure 4C).

**FIGURE 5:**
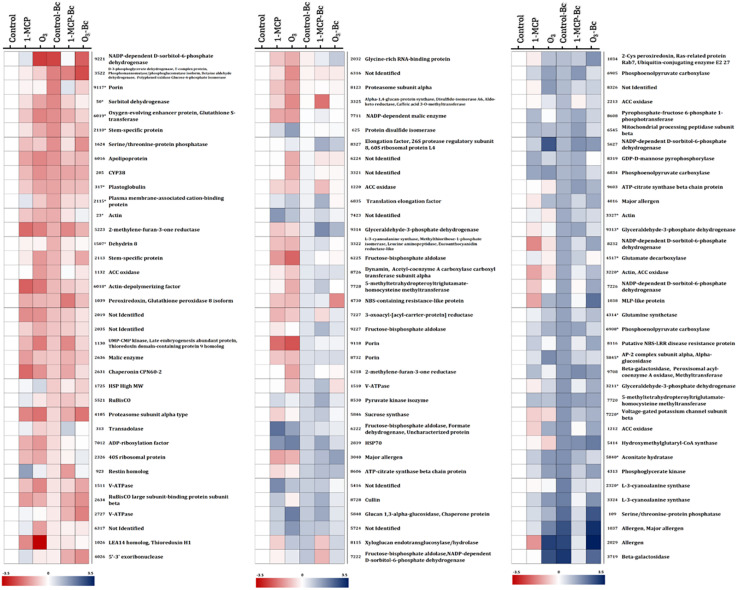
Protein abundance changes among “untreated,” “1-MCP-treated,” and “O_3_-treated” apple fruits (cv. “Granny Smith”) inoculated or not with *Botrytis cinerea* compared with the non-inoculated and untreated apple fruits (control). Heat map of proteins that demonstrated statistically significant differences among the different experimental conditions. Color scale shows the relative abundance of each protein across the experimental conditions, as it has been calculated as log2 of the ratio of the protein abundance of all experimental conditions to the protein abundance to the corresponding control. Blue and red colors indicate, respectively, enhanced and reduced abundance. (*) indicates protein accumulation prevented by O_3_ treatment. Proteins correspond to those listed in Supplementary Tables S1A,B.

Functional analysis showed that 1-MCP application affected several proteins (*n* = 26) that were mainly involved in energy (26.92%), disease/defense (23.08%), and protein destination and storage (15.38%) (Figure 4B). Moreover, most of these proteins were down-accumulated (n = 18), while many of them were involved in energy (27.78%), disease/defense (27.78%), and protein destination and storage (16.67%) (Figure 5). A similar functional classification was observed in up-accumulated (*n* = 8) proteins, which were mainly participated in energy (25%), disease/defense (12.5%), protein destination and storage (12.5%), and metabolism (12.5%) (Figures 4B, 5). The application of O_3_ on apple fruits affected a major part of proteins (*n* = 51) that were mostly involved in disease/defense (25.4%), energy (15.6%), and protein destination and storage (15.6%). Most of these proteins were up-accumulated (*n* = 43) and functionally belong to disease/defense (37.5%), energy (25%), and protein destination and storage (25%). A similar function classification also had the down-accumulated proteins (*n* = 8) with rates of 23.2, 13.95, and 13.95% (Figures 4C, 5).

An overlap of 15 proteins modulated by both 1-MCP and O_3_ treatments was detected, whereas 11 and 36 proteins were affected exclusively by 1-MCP and O_3_ treatments, respectively (Figures 4A,D). These 15 proteins were mainly involved in energy (33.3%), protein destination and storage (26.6%), and disease/defense (13.3%) (Figures 4A,D), whereas the 11 and 36 solely 1-MCP- or O_3_-triggered proteins were associated with disease/defense or their classification was unclear (Figures 4A,D).

### Functional Classification of Proteins Modulated by 1-Methylcyclopropene and O_3_ Treatments in Fruit Artificially Inoculated With *Botrytis cinerea*

**FIGURE 6:**
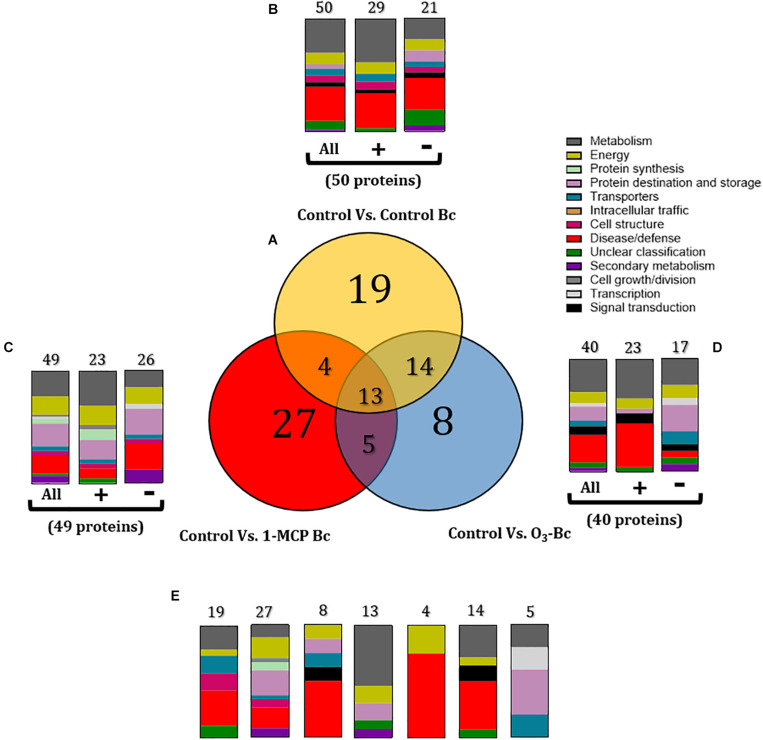
**(A)** Venn diagram demonstrating the common and distinct differentially expressed proteins of 1-methylcyclopropene (1-MCP) and O_3_ application on apple proteome after 60 days of cold storage [1°C, relative humidity (RH) 95%]. Graphical representation of the functional classification of differentially expressed proteins in **(B)** Apple fruits inoculated with *Botrytis cinerea*, **(C)** Apple fruits treated with 1-MCP and inoculated with *B. cinerea*, and **(D)** Apple fruits inoculated with *B. cinerea* and incubated in O_3_ enriched-atmosphere chamber (0.3 μl L^– 1^, 1°C, RH 95%) and **(E)** Functional categorization of the unique and overlapping proteins presented in the Venn diagram **(A)**. Symbols (+) and (-) present the up- and downregulated proteins on each treatment. Control: untreated and non-inoculated fruits. Control Bc: untreated, inoculated with *B. cinerea* fruits. 1-MCP Bc: I-MCP-treated fruits, inoculated with *B. cinerea*. O_3_ Bc: O_3_-treated fruits, inoculated with *B. cinerea*.

Proteome comparisons were also performed to identify proteins modulated by 1-MCP and O_3_ treatments in the presence of *B. cinerea* compared with the untreated and non-inoculated fruits (control). Subsequently, three sets of apple proteins were defined: (1) a set of 50 proteins that were differentiated by the presence of the pathogen in untreated fruits (control vs. control-Bc) (Figure 6B and Supplementary Table S2), (2) a set of 49 proteins that were influenced by the pathogen and 1-MCP treatment (control vs. 1-MCP-Bc) (Figure 6C and Supplementary Table S2), and (3) a set of 40 proteins that were modulated by *B. cinerea* and O_3_ treatment (control vs. O_3_-Bc) (Figure 6D and Supplementary Table S2).

Functional classification revealed that the presence of *B. cinerea* significantly affected apple proteins, mainly those involved in disease/defense, metabolism, energy, and protein destination and storage. Inoculation with the pathogen triggered a great number of proteins (*n* = 50), which operated in disease/defense (30%), metabolism (30%), and energy (10%). In detail, most of these proteins were up-accumulated (*n* = 29) and involved in metabolism (38%) and disease/defense (31%), while down-accumulated proteins (*n* = 21) were associated with disease/defense (29%) and metabolism (19%) (Figures 5, 6B and Supplementary Table S2). The combination of artificial inoculations and 1-MCP treatment affected several proteins (*n* = 49) that are involved in metabolism (22%), protein destination and storage (20%), disease/defense (16%), and energy (16%). A great number of these proteins were downregulated (*n* = 26) and functionally associated with disease/defense (23%), protein destination and storage (23%), metabolism (15%), and energy (15%). A similar functional classification was observed in the up-accumulated proteins, which were associated with metabolism (30%), protein destination and storage (17%), and energy (17%) (Figures 5, 6C and Supplementary Table S2). The combination of artificial inoculations and O_3_ treatment affected 40 proteins that were mostly involved in metabolism (30%), disease/defense (25%), and protein destination and storage (13%). Among these 40 proteins, 23 were upregulated and mainly involved in disease/defense (39%) and metabolism (35%), while 17 proteins were downregulated proteins, and they were associated with metabolism (24%) and protein destination and storage (24%) (Figures 5, 6D and Supplementary Table S2).

Details of shared and distinct affected proteins among the untreated control and the 1-MCP- and O_3_-treated fruits that had been inoculated with *B. cinerea* are shown in Figures 6A,D. The common proteins shared among the three conditions (*n* = 13) were involved in metabolism (54%), protein destination and storage (15.4%), and energy (15.4%) (Figure 6E). Proteins related to disease/defense (31.6%) and metabolism (21%) were predominant in the set of proteins (*n* = 19) exclusively affected by the presence of the pathogen on the untreated fruits (Figure 6E). Common proteins between control and 1-MCP-treated and artificially inoculated fruits (*n* = 4) were associated with disease/defense (75%) and energy (25%), while control’s shared proteins with O_3_-treated and inoculated fruits (*n* = 14) were mainly involved in disease/defense (43%) and metabolism (28%) (Figure 6E). In 1-MCP-treated apple fruits inoculated with *B. cinerea*, a set of unique proteins (*n* = 27) were identified that were mainly related to protein destination and storage (22.2%), disease/defense (18.5%), and energy (18.5%). Proteins triggered uniquely (*n* = 8) by O_3_ treatment and the pathogen were associated with disease/defense (30%), while proteins commonly shared between O_3_- and 1-MCP-treated fruits that had been inoculated with the pathogen (*n* = 5) were mostly associated with protein destination and storage (40%) (Figure 6E).

The changes in apple proteins among untreated control, 1- MCP-, and O_3_-exposed fruits inoculated or not with *B. cinerea* compared with the non-inoculated and untreated fruits (control) are presented in Figure 5. Interestingly, the variations in accumulation levels of *B. cinerea* (Bc)-responsive proteins were prevented by O_3_ treatment compared with control (Figure 5). It is noted that a set of 50 proteins were differentiated by the presence of the pathogen (control vs. control-Bc), whereas the O_3_ treatment prevented the accumulation of almost half of them (*n* = 23), and their abundance was not different compared with that in the control. Such proteins included actin (spot nos. 23, 3,220, and 3,327), sorbitol dehydrogenase (spot no. 50), plastoglobulin (spot no. 317), dedydrin 8 (spot no. 1,507), stem-specific protein (spot no. 2,110), plasma membrane-associated cation-binding protein (spot no. 2,115), L-3-cyanoalanine synthase (spot no. 2,320), glyceraldehyde-3-phosphate dehydrogenase (spot no. 3,211, 9,313), ACCO (spot no. 3,220), glutamine synthetase (spot no. 4,314), glutamate decarboxylase (spot no. 4,517), aconitate hydratase (spot no. 5,840), AP-2 complex subunit alpha (spot no. 5,845), alpha-glucosidase (spot no. 5,845), apolipoprotein (spot no. 6,016), oxygen-evolving enhancer protein (spot no. 6,019), glutathione-*S*-transferase (spot no. 6,019), phosphoenolpyruvate carboxylase (spot no. 6,908), voltage-gated potassium channel subunit beta (spot no. 7,220), and porin (spot no. 9,117) (Figure 5).

## Discussion

Applications of ozone and especially 1-MCP have been widely distributed throughout the world as postharvest treatments to extend the storage period of apple. However, most of these studies focus on the anti-ripening and antimicrobial efficacy of 1-MCP and ozone treatment, respectively, and knowledge concerning the molecular effects of these chemical applications on major postharvest spoilage agents of apple fruits is quite limited. In this study, the effect of the postharvest treatments with 1-MCP and ozone on the development of gray mold caused by *Botrytis cinerea* on apple fruit (cv. “Granny Smith”) was investigated. We further employed a comparative study of the proteomic profiles among non-inoculated and inoculated fruits that were either exposed to 1-MCP and ozone or remain untreated. Such an experimental approach provides insights into the responses of apple fruits to *B. cinerea* infection as well as into the physiological function of 1-MCP and ozone in apple postharvest biology.

The ripening of fleshy fruit is associated with changes in susceptibility to pathogen infection since susceptibility significantly increases during ripening (Papavasileiou et al., 2020). Apple is a typical climacteric fruit, and its ripening process and postharvest behavior is controlled by ethylene (Karagiannis et al., 2018). In line, apple fruit ripening parameters such as SSC, TA, and firmness underwent reduction in untreated control fruits during storage compared with harvest stage. The application of 1-MCP contributed to a retention of fruit physiological characteristics at levels comparable with those during harvest, confirming previous studies (Janisiewicz et al., 2003; Kolniak-Ostek et al., 2014). Moreover, firmness and SSC remained unaffected in O_3_-treated fruits, supporting recent works in apples (Lv et al., 2019; Karagiannis et al., 2020).

The phenotypic characterization of apple fruit responses to *B. cinerea* showed that applications of 1-MCP did not contribute to the suppression of the pathogen. Disease incidence and severity caused by *B. cinerea* in 1-MCP-treated fruits was similar to that observed on untreated control fruits. Unpublished data of our group suggested that 1-MCP applications on another apple cultivar (cv. “Red Delicious”) increased fruit susceptibility to *B. cinerea* (Karaoglanidis, unpublished data). Previous studies on the effect of 1-MCP treatments on the control of *B. cinerea* on several horticultural commodities provided variable results. For example, applications of 1-MCP failed to protect tomato and pear fruits by *B. cinerea* or even led to an increased susceptibility (Díaz et al., 2002; Akagi and Stotz, 2007; Min et al., 2018), while on cut carnation flowers, 1-MCP treatment resulted in a reduction of gray mold incidence and severity (Seglie et al., 2012). However, it should be stated that fruit ripening stage at the time of 1-MCP application may be critical, as has been shown in avocado fruits that were 1-MCP treated and artificially inoculated with *Colletotrichum gloeosporioides* (Wang et al., 2006). In that study, early harvested avocado fruits treated with 1-MCP revealed significant inhibition of fruit ripening and the synthesis of (*Z*,*Z*)-1-acetoxy-2-hydroxy-4-oxo-heneicosa-12,15-diene (AFD), one of the most active antifungal compounds in avocado fruits (Wang et al., 2006).

An important result revealed by this study was that ozone exposure strongly reduced gray mold severity compared with control. *In vitro* studies have shown that ozone treatments in kiwifruit exhibited a strong toxic impact on *B. cinerea* conidia viability and a robust mycostatic activity on pathogen’s mycelial growth (Minas et al., 2010). However, the observed reduction of disease severity on O_3_-treated apple fruits could not be correlated with direct toxic effects of O_3_ on the conidia or mycelial growth. The artificially inoculated fruits were transferred to the ozonated chambers after some hours of incubation at 20°C. This is expected to allow the germination of conidia on fruit surface. Notably, previous studies on the effect of ozone exposure on apple fruits revealed an increased spoilage rate in ozone-treated apple fruits by unspecified spoilage agents (Antos et al., 2018). In contrast, Yaseen et al. (2015) reported a reduction in *Penicillium expansum* population densities on fruit surface. However, in the latter report, only surface fungal population was measured and not disease incidence or disease severity on infected fruits. More recently, in a study aiming to determine the effects of ozone treatments on fruits of different apple cultivars, it was reported that effects on apple fruit spoilage were cultivar dependent (Juhnevica-Radenkova et al., 2019).

To characterize the mechanisms behind the responses of 1-MCP- or O_3_-treated apple to *B. cinerea*, a comparative proteomic analysis was conducted on non-inoculated and artificially inoculated fruits. Initially, we performed a direct comparison of the proteome of fruits that were subjected to the two postharvest treatments tested, without artificial inoculation with *B. cinerea*. Using this approach, we determined differences in the protein abundance imposed by 1-MCP and ozone in the absence of pathogen that could be associated with the observed phenotypic response to *B. cinerea*. Data indicated that ozone induced a stronger alteration in non-inoculated fruit proteome compared with protein changes caused by 1-MCP. Such as greater protein impact of ozone treatment compared with that imposed by 1-MCP application has been described in kiwifruit (Minas et al., 2018). Interestingly, most of the changed ozone-responsive proteins were found to be up-accumulated, while, in contrast, most of the proteins changed by 1-MCP were found to be down-accumulated. The majority of the upregulated proteins in the O_3_-treated fruits were primarily involved in disease/defense. The obvious absence of defense-related proteins triggering by 1-MCP may explain, therefore, its failure to provide an efficient protection against *B. cinerea*. A similar down-accumulation of defense proteins, such as the PR proteins, has been observed in 1-MCP-treated kiwifruit (Minas et al., 2018) or banana fruits (Kesari et al., 2010). In contrast, in O_3_-treated fruits, both in the absence and in the presence of the pathogen, an upregulation of proteins belonging to the groups of allergen and major allergens was observed.

PR proteins are among the most widespread allergen proteins associated with plants, and they are, currently, organized in 17 distinct families. PR proteins are mainly induced by plant pathogens, but in addition, they can be synthesized in response to abiotic factors such as wounding (Buron-Moles et al., 2014; Sinha et al., 2014). Since ozone is a strong oxidative agent, it is possible that fruit exposure to it may induce the observed upregulation of PR proteins. In apple fruits, allergen proteins, belonging to diverse PR protein families, have been associated with fruit resistance to several fungal and bacterial pathogens, including *B. cinerea* (Krebitz et al., 2003; Beuning et al., 2004; Ballester et al., 2017; Banani et al., 2018). For instance, a thaumatin-like (PR-5) protein and a class III chitinase (PR-8) were associated with apple fruit resistance to *B. cinerea* (Banani et al., 2018). Similarly, three Mal d 1 proteins (PR-10 family proteins) were found to be induced in apple fruits in response to stress imposed by abiotic factors such as wounding and biotic factors such as the apple pathogen *P. expansum* or the non-apple pathogen *Penicillium digitatum* (Buron-Moles et al., 2015). Interestingly, some of these were upregulated by both *Penicillium* spp., while for at least one of them, a higher induction rate was observed in fruit challenged with the apple non-pathogen *P. digitatum*, providing evidence for its role in the resistance of apple fruits to *P. digitatum* (Buron-Moles et al., 2015). In another study aimed to determine the proteome profile of apple varieties susceptible and resistant to *Alternaria alternata*, another necrotrophic pathogen of apple, two major allergens, a glucanase (PR-2 family), and a thaumatin-like protein (PR-5 family) were associated with resistance to this disease (Ni et al., 2017). In addition to apple fruits, allergen and major allergen PR proteins have been found to play a major role in the tolerance to *B. cinerea* infections on several other hosts such as strawberry or grape (Monteiro et al., 2003; Haile et al., 2019). Taking into account the variability and complexity that exist within the group of allergen and major allergen proteins of apple fruits, further studies are required to determine precisely the role of each of them in the response of apple fruits to the stress imposed by *B. cinerea* or other spoilage agents and to understand how they regulate the defense responses of the host.

Dynamic changes in fruit proteome were observed in the presence of *B. cinerea* in all the types of fruits tested (control, 1-MCP treated, and O_3_ treated). A similar strong modulation of host proteome by pathogen infection has been described in numerous previous reports including the interaction of *B. cinerea* with tomato (Tzortzakis, 2019). Interestingly, in O_3_-treated fruits, a significant number of disease/defense-related proteins were upregulated in comparison with the control fruits. Among these disease/defense-related proteins, the higher accumulation levels were observed for allergen, major allergen, putative nucleotide-binding site leucine-rich repeat (NBS-LRR) disease resistance protein, major latex protein (MLP)-like protein, and 2-Cys peroxiredoxin (2-Cys Prx). All these proteins have been found to play a significant role in the interactions of several plant species including apple with biotic stress factors, and most of them are induced by the ethylene signaling pathway. The critical role of ethylene in the initiation of defense responses of apple fruits against *B. cinerea* had previously been determined (Akagi et al., 2011). Herein, ACO, which catalyzes the last steps of ethylene biosynthesis, was induced by *B. cinerea* in the O_3_-treated fruits, while it remained unaffected by *B. cinerea* in 1-MCP-treated fruits. Moreover, ACO was down-accumulated in non-inoculated fruits that were treated with 1-MCP or exposed to ozone, confirming previous observations (Minas et al., 2018). A similar down-accumulation of ACO was observed in O_3_-exposed tomato fruits (Tzortzakis et al., 2013). However, on the same fruits, ACO was also found to be suppressed in O_3_-treated fruits challenged with *B. cinerea* (Tzortzakis et al., 2013; Tzortzakis, 2019). Our data suggest that ACO was up-accumulated by the pathogen’s presence only in O_3_-treated apple fruits. Thus, the observed relative resistance of ozone-exposed fruits to *B. cinerea* infections may be explained by the induction of ethylene signaling that would initiate defense responses.

The current proteomic analysis further indicated that 2-Cys Prx was specifically up-accumulated in the O_3_-exposed fruits that had been inoculated with *B. cinerea*. 2-Cys Prx is a distinct subgroup within the family of peroxiredoxins (PRXs) that are associated with resistance of plants to both biotic and abiotic stress, while in apple fruits, their downregulation has been linked to *A. alternata* susceptibility (Borges et al., 2013; Ni et al., 2017; Ojeda et al., 2018). Given that PRXs are highly conserved antioxidant proteins that scavenge reactive oxygen species (ROS) (Zhang et al., 2019), it can be hypothesized that the increased abundance of 2-Cys Prx in O_3_-treated fruit reduces ROS level in the host and protects apple cells from the pathogen invasion. It is well established that production of ROS is one of the earliest response of plant tissues to pathogen attacks (Lamb and Dixon, 1997). However, this defense response is primarily effective against biotrophic pathogens, while necrotrophic pathogens such as *B. cinerea* may take advantage in the infection process of host tissues by the generation of ROS produced by the host or even *B. cinerea* itself (Govrin and Levine, 2000; Heller and Tudzynski, 2011). In our study, several ROS-related proteins such as PRX, glutathione peroxidase 8 isoform, oxygen-evolving enhancer protein, and glutathione *S*-transferase were depressed in 1-MCP-treated fruits that were challenged with *B. cinerea*. These proteins are part of the antioxidant system of the plants participating in their redox homeostasis by suppressing ROS accumulation (Ozyigit et al., 2016; Gullner et al., 2018), while many of them are considered as ripening-induced proteins in apple fruits and other commodities (Nishitani et al., 2010; Zheng et al., 2013; Shi et al., 2014). The observed down-accumulation of these proteins in apple exposed to both 1-MCP and *B. cinerea* suggests that high ROS accumulation occurs in fruits, which facilitates pathogen growth and contributes to the increased susceptibility of 1-MCP-treated apple to pathogen.

The upregulation of the NBS-LRR protein in the O_3_-treated fruits in the presence of *B. cinerea* was another interesting finding of this study. Several putative NBS-LRR proteins have been identified from the whole genome data set of apple, although functional characterization was not conducted (Arya et al., 2014). The NBS-LRR proteins are considered R gene products, and their overexpression is usually associated with resistance to biotrophic or hemibiotrophic pathogens (Galli et al., 2010). However, recently, it was demonstrated that an NBS-LRR protein confers resistance of wheat to *Rhizoctonia cerealis* (Zhu et al., 2017). An induction of an NBS-LRR protein encoding gene was observed in ripe strawberry fruits inoculated by *B. cinerea*, while the same gene was found to be downregulated in unripe and pathogen-tolerant fruits (Haile et al., 2019). Therefore, taking into account that NBS-LRR proteins are involved in hypersensitive responses at the site of pathogen entry, it was proposed that their upregulation may assist *B. cinerea* in infecting the ripe strawberry fruits (Haile et al., 2019). Whether a similar response occurs in O_3_-treated fruits challenged with *B. cinerea* remains to be further investigated.

The last defense-associated protein found to be upregulated in the O_3_-exposed fruits inoculated with *B. cinerea* was the MLP. MLP and the orthologs of MLP (MLP-like protein) belong to the PR10 family and have been found in several plant species, including apple, and most of them are expressed during fruit ripening (He et al., 2020; Song et al., 2020). Although the precise molecular basis of protection of plants by MLPs against plant pathogens currently is unclear, evidence has been provided for their induction on several plant hosts against a wide range of plant pathogens such as fungi, viruses, and phytoplasmas (Segarra et al., 2013; Yang et al., 2015; Gai et al., 2018; Song et al., 2020).

In the current study, we focused on the determination of dynamic changes in apple fruit proteome in response to 1-MCP or ozone treatments. However, the same treatments may also affect the proteome and the secretome of the fungus itself. It is already known from previous studies that the biochemical characteristics of the fungus growth substrate may strongly influence its secretome that plays a crucial role in its pathogenicity (Shah et al., 2009; Li et al., 2012). For instance, it has been shown that in host tissues of lower pH, the fungus preferentially secretes proteins associated with proteolysis rather than cell wall-degrading enzymes (CWDEs), while in host tissues of higher pH, CWDEs are preferentially secreted by *B. cinerea* (Li et al., 2012). Future research on the role of the tested postharvest treatments on the differential induction of fungus secretome could provide further explanations for the observed tolerance of O_3_-treated apple fruits to gray mold.

## Conclusion

Data presented in the current study represent a first attempt to investigate the impact of 1-MCP and ozone applications on the development of *Botrytis cinerea*, a major agent of postharvest decay of “Granny Smith” apple fruit. Although 1-MCP was not able to affect the *B. cinerea* development, ozone treatment effectively suppressed the disease severity. Proteomic analysis has allowed the detection of various apple fruit proteins and their specific accumulation pattern in response to 1-MCP and ozone treatments in the presence and the absence of *B. cinerea*, thus allowing an identification of differentially accumulated proteins in both types of interactions. Fruits treated with ozone exhibited larger proteome programming than 1-MCP exposed apples and possessed a more sensitive defense system when the fruits were challenged with subsequent *B. cinerea* infection. The current study provides a deeper understanding of the interactions among 1-MCP or ozone treatments and *B. cinerea* in apple fruits and underlines the mechanism of action of the two postharvest applications against gray mold.

## Data Availability Statement

The datasets presented in this study can be found in online repositories. The names of the repository/repositories and accession number(s) can be found in the article/Supplementary Material.

## Author Contributions

GT, IM, AM, and GK designed the experiments. ST performed the experiments. ST, MS, and GT performed analysis and interpretation of the data. ST and GK drafted the manuscript. IM, AM, GT, and GK revised the manuscript. All authors revised the manuscript and approved the final version to be published.

## Conflict of Interest

The authors declare that the research was conducted in the absence of any commercial or financial relationships that could be construed as a potential conflict of interest.

## Footnotes

1 www.graphpad.com
