# Supplementary material for: Unraveling Interactions of the Necrotrophic Fungal Species Botrytis cinerea With 1-Methylcyclopropene or Ozone-Treated Apple Fruit Using Proteomic Analysis
Source: Front Plant Sci. 2021 Mar 10;12:644255. doi: 10.3389/fpls.2021.644255 (PMC7988217; doi:10.3389/fpls.2021.644255)
Supplement: Supplementary file 1 [file Table_1.DOCX]

**Table S1A**. Identification details of differentiable proteins in the mesocarp of untreated, 1-MCP and O_3_ treated apples (cv. Granny Smith) inoculated/or not with the pathogen *B. cinerea* after of 60 days of cold storage.

| Spot N^o^ **^a^** | Accession Number**^b^** | Suggested Name**^c^** | Matching criteria**^d^** | Organism **^e^** | Subcellular Localization**^f^** | Functional category**^g^** | Score | Coverage | # Proteins | # Unique Peptides | # Peptides | # PSMs | # AAs | MW [kDa] | calc. pI | Peptide confidence | Sequence | # PSMs | # Proteins | # Protein Groups | Protein Group Accessions | Modifications | ΔCn | XCorr | Charge | MH+ [Da] | ΔM [ppm] | RT [min] | # Missed Cleavages |
| --- | --- | --- | --- | --- | --- | --- | --- | --- | --- | --- | --- | --- | --- | --- | --- | --- | --- | --- | --- | --- | --- | --- | --- | --- | --- | --- | --- | --- | --- |
| 23 | gi\|657976500 | Actin |  | *Malus domestica* | Cytoskeleton | 09.04-Cell structure/Cytosk  eleton | 37.42 | 15.12 | 10 | 2 | 6 | 28 | 377 | 41.7 | 5.49 | High | VAPEEHPVLLTEA PLNPK | 16 | 7 | 1 | 657976500 |  | 0.00 | 3.82 | 3 | 1954.07 | 0.59 | 42.01 | 0 |
|  |  |  |  |  |  |  |  |  |  |  |  |  |  |  |  | High | AGFAGDDAPR | 3 | 10 | 1 | 657976500 |  | 0.00 | 3.41 | 2 | 976.45 | -0.19 | 37.96 | 0 |
|  |  |  |  |  |  |  |  |  |  |  |  |  |  |  |  | Low | GYSFTTTAER | 5 | 8 | 1 | 657976500 |  | 0.00 | 2.36 | 2 | 1132.53 | -0.05 | 40.02 | 0 |
|  |  |  |  |  |  |  |  |  |  |  |  |  |  |  |  | Low | CDVDIRK | 1 | 10 | 1 | 657976500 | C1(Carbamidomethyl) | 0.03 | 1.36 | 2 | 905.45 | -0.34 | 34.86 | 1 |
|  |  |  |  |  |  |  |  |  |  |  |  |  |  |  |  | Low | LDLAGR | 1 | 10 | 1 | 657976500 |  | 0.00 | 1.29 | 2 | 644.37 | -0.05 | 38.77 | 0 |
|  |  |  |  |  |  |  |  |  |  |  |  |  |  |  |  | Low | GILTLK | 2 | 10 | 1 | 657976500 |  | 0.00 | 0.85 | 2 | 644.43 | 0.31 | 10.37 | 0 |
| 317 | gi\|657963783 | Plastoglobulin | 100% to Prunus mume gi\|645231139  E-value: 0.0 | *Malus domestica* | Chloroplastic | 02.07-  Energy/Pentose phosphate | 5.44 | 5.63 | 1 | 1 | 2 | 2 | 355 | 38.0 | 4.83 | High | GDGGLFVLAR | 1 | 1 | 1 | 657963783 |  | 0.00 | 3.31 | 2 | 1004.55 | -0.07 | 43.00 | 0 |
|  |  |  |  |  |  |  |  |  |  |  |  |  |  |  |  | Low | EGSFQPPEIK | 1 | 1 | 1 | 657963783 |  | 0.00 | 2.13 | 2 | 1131.57 | 0.04 | 38.71 | 0 |
| 923 | gi\|658013978 | Restin |  | *Malus domestica* | Nucleus | 20.1-Secondary metabolism/Phen ylpropanoids/Phe  nolics | 146.30 | 38.32 | 2 | 22 | 56 | 113 | 1378 | 154.4 | 4.70 | High | DGSHAAETALVE DDKPSVIER | 1 | 1 | 1 | 658013978 |  | 0.00 | 3.68 | 3 | 2239.09 | 0.96 | 39.82 | 0 |
|  |  |  |  |  |  |  |  |  |  |  |  |  |  |  |  | High | LQEALGSFTNR | 2 | 1 | 1 | 658013978 |  | 0.00 | 3.67 | 2 | 1235.64 | 0.74 | 41.93 | 0 |
|  |  |  |  |  |  |  |  |  |  |  |  |  |  |  |  | High | LTEDVSTYESK | 2 | 2 | 1 | 658013978 |  | 0.00 | 3.55 | 2 | 1271.60 | 0.73 | 38.86 | 0 |
|  |  |  |  |  |  |  |  |  |  |  |  |  |  |  |  | High | LQEQITEAEEK | 2 | 2 | 1 | 658013978 |  | 0.00 | 3.30 | 2 | 1317.65 | 0.23 | 37.44 | 0 |
|  |  |  |  |  |  |  |  |  |  |  |  |  |  |  |  | High | LKSAEEQLEQQGK | 3 | 2 | 1 | 658013978 |  | 0.00 | 3.29 | 3 | 1487.77 | 0.06 | 36.71 | 1 |
|  |  |  |  |  |  |  |  |  |  |  |  |  |  |  |  | High | TSSLEVALQAANE  K | 2 | 1 | 1 | 658013978 |  | 0.00 | 3.17 | 2 | 1460.76 | 0.68 | 43.40 | 0 |
|  |  |  |  |  |  |  |  |  |  |  |  |  |  |  |  | High | AVAAQENLAIVTK | 2 | 1 | 1 | 658013978 |  | 0.00 | 3.15 | 2 | 1327.76 | 0.68 | 41.28 | 0 |
|  |  |  |  |  |  |  |  |  |  |  |  |  |  |  |  | High | SAEEQLEQQGK | 3 | 2 | 1 | 658013978 | Q5(Deamidated) | 0.00 | 3.12 | 2 | 1247.58 | 1.03 | 36.96 | 0 |
|  |  |  |  |  |  |  |  |  |  |  |  |  |  |  |  | High | LSDLEVEVER | 1 | 1 | 1 | 658013978 |  | 0.00 | 3.12 | 2 | 1188.61 | 0.45 | 41.29 | 0 |
|  |  |  |  |  |  |  |  |  |  |  |  |  |  |  |  | High | ELFEALNVATEEK | 1 | 2 | 1 | 658013978 |  | 0.00 | 3.09 | 2 | 1492.75 | 1.27 | 45.58 | 0 |
|  |  |  |  |  |  |  |  |  |  |  |  |  |  |  |  | High | LEDASSSFTEK | 2 | 2 | 1 | 658013978 |  | 0.00 | 3.03 | 2 | 1213.56 | 0.35 | 38.97 | 0 |
|  |  |  |  |  |  |  |  |  |  |  |  |  |  |  |  | High | LNALQGQVK | 2 | 1 | 1 | 658013978 | Q5(Deamidated) | 0.00 | 2.91 | 2 | 971.55 | 0.23 | 38.91 | 0 |
|  |  |  |  |  |  |  |  |  |  |  |  |  |  |  |  | High | QLTGQVQEYQEK | 2 | 2 | 1 | 658013978 |  | 0.00 | 2.91 | 2 | 1450.72 | 0.95 | 38.26 | 0 |
|  |  |  |  |  |  |  |  |  |  |  |  |  |  |  |  | High | GDGGSVAGLEVK | 1 | 2 | 1 | 658013978 |  | 0.00 | 2.89 | 2 | 1088.56 | -0.02 | 39.24 | 0 |
|  |  |  |  |  |  |  |  |  |  |  |  |  |  |  |  | High | NAELEESLSK | 2 | 2 | 1 | 658013978 |  | 0.00 | 2.85 | 2 | 1119.55 | 0.47 | 39.43 | 0 |
|  |  |  |  |  |  |  |  |  |  |  |  |  |  |  |  | High | DLEANELLEK | 2 | 1 | 1 | 658013978 |  | 0.00 | 2.69 | 2 | 1173.60 | 0.28 | 43.78 | 0 |
|  |  |  |  |  |  |  |  |  |  |  |  |  |  |  |  | High | FASSESTNEELR | 1 | 1 | 1 | 658013978 |  | 0.00 | 2.51 | 2 | 1369.62 | 0.64 | 37.36 | 0 |
|  |  |  |  |  |  |  |  |  |  |  |  |  |  |  |  | Medium | RLEDASSSFTEK | 2 | 2 | 1 | 658013978 |  | 0.00 | 2.48 | 2 | 1369.66 | 0.63 | 38.09 | 1 |
|  |  |  |  |  |  |  |  |  |  |  |  |  |  |  |  | Medium | VSELEEIK | 1 | 2 | 1 | 658013978 |  | 0.00 | 2.46 | 2 | 946.51 | -0.06 | 40.06 | 0 |
|  |  |  |  |  |  |  |  |  |  |  |  |  |  |  |  | Medium | ESETAFATATEK | 2 | 1 | 1 | 658013978 |  | 0.00 | 2.44 | 2 | 1284.60 | 0.89 | 39.12 | 0 |
|  |  |  |  |  |  |  |  |  |  |  |  |  |  |  |  | Medium | DAEANSLLEK | 2 | 2 | 1 | 658013978 |  | 0.00 | 2.43 | 2 | 1089.54 | 1.55 | 40.00 | 0 |
|  |  |  |  |  |  |  |  |  |  |  |  |  |  |  |  | Medium | ALDLHSASEAR | 3 | 1 | 1 | 658013978 |  | 0.00 | 2.43 | 2 | 1169.59 | 0.22 | 37.39 | 0 |
|  |  |  |  |  |  |  |  |  |  |  |  |  |  |  |  | Low | SAEEQLEQQGK | 1 | 2 | 1 | 658013978 |  | 0.00 | 2.33 | 2 | 1246.59 | 0.25 | 35.86 | 0 |
|  |  |  |  |  |  |  |  |  |  |  |  |  |  |  |  | Low | DIGSTVSTPSK | 2 | 1 | 1 | 658013978 |  | 0.00 | 2.25 | 2 | 1091.56 | 0.31 | 38.21 | 0 |
|  |  |  |  |  |  |  |  |  |  |  |  |  |  |  |  | Low | DLLNEQVTK | 1 | 2 | 1 | 658013978 |  | 0.00 | 2.25 | 2 | 1059.57 | 0.05 | 41.00 | 0 |
|  |  |  |  |  |  |  |  |  |  |  |  |  |  |  |  | Low | LNALQGQVK | 2 | 1 | 1 | 658013978 |  | 0.00 | 2.25 | 2 | 970.57 | 0.05 | 38.65 | 0 |
|  |  |  |  |  |  |  |  |  |  |  |  |  |  |  |  | Low | VSNGEVPPVEK | 1 | 1 | 1 | 658013978 | N3(Deamidated) | 0.00 | 2.22 | 2 | 1155.59 | 1.82 | 37.30 | 0 |
|  |  |  |  |  |  |  |  |  |  |  |  |  |  |  |  | Low | KDDLLSQSLSNNA  ELEQK | 1 | 1 | 1 | 658013978 |  | 0.00 | 2.17 | 3 | 2032.02 | 0.87 | 41.96 | 1 |
|  |  |  |  |  |  |  |  |  |  |  |  |  |  |  |  | Low | NSELQALHETLVR | 3 | 1 | 1 | 658013978 |  | 0.00 | 2.14 | 2 | 1509.80 | 1.14 | 42.60 | 0 |
|  |  |  |  |  |  |  |  |  |  |  |  |  |  |  |  | Low | LQEQITEAEEK | 2 | 2 | 1 | 658013978 | Q4(Deamidated) | 0.00 | 2.11 | 2 | 1318.64 | 0.41 | 38.53 | 0 |
|  |  |  |  |  |  |  |  |  |  |  |  |  |  |  |  | Low | FIAVEQK | 1 | 1 | 1 | 658013978 |  | 0.00 | 2.07 | 2 | 834.47 | 0.23 | 39.19 | 0 |
|  |  |  |  |  |  |  |  |  |  |  |  |  |  |  |  | Low | SASLKEELDNSLA  K | 3 | 2 | 1 | 658013978 |  | 0.00 | 2.05 | 2 | 1504.79 | 1.25 | 41.58 | 1 |
|  |  |  |  |  |  |  |  |  |  |  |  |  |  |  |  | Low | TIEDLMQQHSSEG  QK | 3 | 1 | 1 | 658013978 | M6(Oxidation) | 0.00 | 2.02 | 2 | 1746.80 | 1.08 | 37.33 | 0 |
|  |  |  |  |  |  |  |  |  |  |  |  |  |  |  |  | Low | EKLSDLEVEVER | 1 | 1 | 1 | 658013978 |  | 0.02 | 1.98 | 3 | 1445.75 | -0.50 | 41.43 | 1 |
|  |  |  |  |  |  |  |  |  |  |  |  |  |  |  |  | Low | NKEIALLDK | 3 | 2 | 1 | 658013978 |  | 0.00 | 1.96 | 2 | 1043.61 | -0.28 | 39.15 | 1 |
|  |  |  |  |  |  |  |  |  |  |  |  |  |  |  |  | Low | SLEELEAK | 2 | 1 | 1 | 658013978 |  | 0.00 | 1.93 | 2 | 918.48 | -0.21 | 40.10 | 0 |
|  |  |  |  |  |  |  |  |  |  |  |  |  |  |  |  | Low | LQEAIGR | 2 | 1 | 1 | 658013978 |  | 0.00 | 1.91 | 2 | 786.45 | 0.02 | 37.27 | 0 |
|  |  |  |  |  |  |  |  |  |  |  |  |  |  |  |  | Low | LHSELQLAHATVA  EK | 3 | 1 | 1 | 658013978 |  | 0.00 | 1.84 | 3 | 1646.89 | 0.09 | 39.82 | 0 |
|  |  |  |  |  |  |  |  |  |  |  |  |  |  |  |  | Low | LQLADTK | 1 | 1 | 1 | 658013978 |  | 0.00 | 1.81 | 2 | 788.45 | 0.23 | 37.51 | 0 |
|  |  |  |  |  |  |  |  |  |  |  |  |  |  |  |  | Low | STVEELTDKHSR | 2 | 1 | 1 | 658013978 |  | 0.00 | 1.79 | 3 | 1401.70 | -0.15 | 38.18 | 1 |
|  |  |  |  |  |  |  |  |  |  |  |  |  |  |  |  | Low | VYEEHXAEAER | 3 | 1 | 1 | 658013978 | X6(L) | 0.00 | 1.79 | 2 | 1345.64 | 0.02 | 36.86 | 0 |

|  |  |  |  |  |  |  |  |  |  |  |  |  |  |  |  | Low | EMEDQMASIQGE  LK | 2 | 2 | 1 | 658013978 | M2(Oxidation);  M6(Oxidation) | 0.00 | 1.75 | 2 | 1640.72 | 1.02 | 38.68 | 0 |
| --- | --- | --- | --- | --- | --- | --- | --- | --- | --- | --- | --- | --- | --- | --- | --- | --- | --- | --- | --- | --- | --- | --- | --- | --- | --- | --- | --- | --- | --- |
|  |  |  |  |  |  |  |  |  |  |  |  |  |  |  |  | Low | SQGVDLEEK | 2 | 1 | 1 | 658013978 |  | 0.00 | 1.71 | 2 | 1004.49 | 0.20 | 37.91 | 0 |
|  |  |  |  |  |  |  |  |  |  |  |  |  |  |  |  | Low | DETVEQLQAAK | 2 | 2 | 1 | 658013978 |  | 0.00 | 1.71 | 2 | 1231.62 | 0.37 | 38.61 | 0 |
|  |  |  |  |  |  |  |  |  |  |  |  |  |  |  |  | Low | VSELELMLETEK | 1 | 2 | 1 | 658013978 | M7(Oxidation) | 0.00 | 1.64 | 2 | 1436.72 | 1.24 | 42.77 | 0 |
|  |  |  |  |  |  |  |  |  |  |  |  |  |  |  |  | Low | TSSLEVALQAANE  KER | 1 | 1 | 1 | 658013978 |  | 0.00 | 1.62 | 2 | 1745.91 | 1.74 | 41.60 | 1 |
|  |  |  |  |  |  |  |  |  |  |  |  |  |  |  |  | Low | LYEEQVR | 2 | 2 | 1 | 658013978 |  | 0.00 | 1.59 | 2 | 936.48 | -0.25 | 37.77 | 0 |
|  |  |  |  |  |  |  |  |  |  |  |  |  |  |  |  | Low | LKLSEENFGK | 1 | 1 | 1 | 658013978 |  | 0.17 | 1.59 | 3 | 1164.63 | 0.43 | 39.09 | 1 |
|  |  |  |  |  |  |  |  |  |  |  |  |  |  |  |  | Low | FASSESTNEELRK | 6 | 1 | 1 | 658013978 |  | 0.00 | 1.57 | 3 | 1497.72 | -0.01 | 36.60 | 1 |
|  |  |  |  |  |  |  |  |  |  |  |  |  |  |  |  | Low | KLQEQITEAEEK | 3 | 2 | 1 | 658013978 |  | 0.00 | 1.54 | 2 | 1445.75 | 0.04 | 38.74 | 1 |
|  |  |  |  |  |  |  |  |  |  |  |  |  |  |  |  | Low | STVEELTDK | 2 | 1 | 1 | 658013978 |  | 0.00 | 1.50 | 2 | 1021.51 | 0.57 | 39.04 | 0 |
|  |  |  |  |  |  |  |  |  |  |  |  |  |  |  |  | Low | HSESENSELKHEV  LLTK | 1 | 1 | 1 | 658013978 |  | 0.00 | 1.48 | 4 | 1980.00 | -0.19 | 38.94 | 1 |
|  |  |  |  |  |  |  |  |  |  |  |  |  |  |  |  | Low | VYEEHXAEAERK | 1 | 1 | 1 | 658013978 | X6(L) | 0.00 | 1.41 | 3 | 1473.73 | 0.26 | 37.25 | 1 |
|  |  |  |  |  |  |  |  |  |  |  |  |  |  |  |  | Low | SEIENLK | 1 | 1 | 1 | 658013978 |  | 0.01 | 1.37 | 2 | 832.44 | 0.04 | 37.20 | 0 |
|  |  |  |  |  |  |  |  |  |  |  |  |  |  |  |  | Low | YSTAVVEK | 1 | 2 | 1 | 658013978 |  | 0.00 | 1.36 | 2 | 896.47 | -0.14 | 36.40 | 0 |
|  |  |  |  |  |  |  |  |  |  |  |  |  |  |  |  | Low | AEVAEKPLLQNSL  K | 4 | 2 | 1 | 658013978 |  | 0.00 | 1.36 | 2 | 1539.88 | 0.65 | 40.35 | 0 |
|  |  |  |  |  |  |  |  |  |  |  |  |  |  |  |  | Low | ALEFEK | 2 | 2 | 1 | 658013978 |  | 0.03 | 1.32 | 2 | 736.39 | -0.17 | 40.01 | 0 |
|  |  |  |  |  |  |  |  |  |  |  |  |  |  |  |  | Low | VSNGEVPPVEK | 1 | 1 | 1 | 658013978 |  | 0.00 | 1.32 | 2 | 1154.61 | 0.45 | 36.40 | 0 |
|  |  |  |  |  |  |  |  |  |  |  |  |  |  |  |  | Low | VSNGEVPPVEKER | 1 | 1 | 1 | 658013978 | N3(Deamidated) | 0.06 | 1.29 | 3 | 1440.73 | -0.02 | 36.53 | 1 |
|  |  |  |  |  |  |  |  |  |  |  |  |  |  |  |  | Low | VSELEEIKLK | 1 | 2 | 1 | 658013978 |  | 0.00 | 1.04 | 3 | 1187.69 | 0.15 | 40.49 | 1 |
| 1026 | gi\|657942890 | Desiccation protectant protein Lea14 homolog |  | *Malus domestica* | Unclear | 11.05-  Disease/Defense  /Stress responses | 29.88 | 28.67 | 1 | 4 | 4 | 17 | 150 | 16.5 | 5.55 | High | VIASGTIPDPGSLK | 3 | 1 | 1 | 657942890 |  | 0.00 | 3.83 | 2 | 1354.76 | 1.00 | 42.39 | 0 |
|  |  |  |  |  |  |  |  |  |  |  |  |  |  |  |  | High | MGLSHVEYLSK | 9 | 1 | 1 | 657942890 | M1(Oxidation) | 0.00 | 3.63 | 2 | 1279.64 | 0.88 | 39.66 | 0 |
|  |  |  |  |  |  |  |  |  |  |  |  |  |  |  |  | High | ISNVAKPEAEVTD  VDFK | 3 | 1 | 1 | 657942890 |  | 0.00 | 2.89 | 3 | 1861.95 | -0.60 | 40.38 | 0 |
|  |  |  |  |  |  |  |  |  |  |  |  |  |  |  |  | High | ISNVAKPEAEVTD  VDFKK | 2 | 1 | 1 | 657942890 |  | 0.00 | 2.58 | 3 | 1990.05 | 1.71 | 39.63 | 1 |
| 1026 | gi\|657991543 | Thioredoxin |  | *Malus domestica* | Cytosol | 02.20-  Energy/Electron- transport | 18.93 | 25.60 | 3 | 2 | 4 | 9 | 125 | 13.7 | 5.55 | High | HAAPADVPADVA TASA | 2 | 1 | 1 | 657991543 |  | 0.00 | 2.90 | 2 | 1463.71 | 1.08 | 39.39 | 0 |
|  |  |  |  |  |  |  |  |  |  |  |  |  |  |  |  | High | KNPEVTFLK | 4 | 3 | 1 | 657991543 |  | 0.00 | 2.70 | 3 | 1075.61 | -0.07 | 39.82 | 1 |
|  |  |  |  |  |  |  |  |  |  |  |  |  |  |  |  | Low | VDVDELK | 2 | 3 | 1 | 657991543 |  | 0.00 | 2.29 | 2 | 817.43 | 1.12 | 39.99 | 0 |
|  |  |  |  |  |  |  |  |  |  |  |  |  |  |  |  | Low | NPEVTFLK | 1 | 3 | 1 | 657991543 |  | 0.00 | 1.96 | 2 | 947.52 | -0.19 | 42.15 | 0 |
| 1034 | gi\|657999067 | 2-Cys peroxiredoxin  BAS1 |  | *Malus domestica* | Chloroplast | 11.06-  Disease/Defense  /Detoxification | 19.53 | 17.71 | 1 | 1 | 5 | 14 | 271 | 29.4 | 7.97 | High | EGVIQHSTVNNLA IGR | 7 | 1 | 1 | 657999067 |  | 0.00 | 2.85 | 3 | 1707.91 | 0.50 | 41.16 | 0 |
|  |  |  |  |  |  |  |  |  |  |  |  |  |  |  |  | Low | GSKEYFSAI | 2 | 1 | 1 | 657999067 |  | 0.00 | 1.99 | 2 | 1001.49 | 0.32 | 43.29 | 1 |
|  |  |  |  |  |  |  |  |  |  |  |  |  |  |  |  | Low | YPLISDVTK | 1 | 1 | 1 | 657999067 |  | 0.00 | 1.85 | 2 | 1035.57 | -0.05 | 43.44 | 0 |
|  |  |  |  |  |  |  |  |  |  |  |  |  |  |  |  | Low | GLFIIDK | 2 | 1 | 1 | 657999067 |  | 0.00 | 1.81 | 2 | 805.48 | 0.30 | 46.00 | 0 |
|  |  |  |  |  |  |  |  |  |  |  |  |  |  |  |  | Low | LSEYIGK | 2 | 1 | 1 | 657999067 |  | 0.00 | 1.54 | 2 | 809.44 | 0.21 | 39.08 | 0 |
| 1034 | gi\|657999259 | Ras-related protein Rab7 |  | *Malus domestica* | Nucleus | 10.04-Signal  transduction/Me diators | 8.56 | 27.67 | 3 | 2 | 5 | 6 | 206 | 23.1 | 5.08 | High | TSLMNQYVNK | 1 | 1 | 1 | 657999259 | M4(Oxidation) | 0.00 | 2.57 | 2 | 1213.59 | 0.50 | 37.47 | 0 |
|  |  |  |  |  |  |  |  |  |  |  |  |  |  |  |  | High | VIILGDSGVGK | 2 | 3 | 1 | 657999259 |  | 0.00 | 2.26 | 2 | 1057.63 | 0.27 | 43.15 | 0 |
|  |  |  |  |  |  |  |  |  |  |  |  |  |  |  |  | Low | GADCCVLVYDVN SMK | 1 | 1 | 1 | 657999259 | C4(Carbamidomethyl);  C5(Carbamidomethyl); M14(Oxidation) | 0.00 | 1.72 | 2 | 1746.75 | 1.63 | 42.33 | 0 |
|  |  |  |  |  |  |  |  |  |  |  |  |  |  |  |  | Low | ATIGADFLTK | 1 | 3 | 1 | 657999259 |  | 0.00 | 1.57 | 2 | 1036.57 | 0.39 | 43.58 | 0 |
|  |  |  |  |  |  |  |  |  |  |  |  |  |  |  |  | Low | GNIPYYETSAK | 1 | 1 | 1 | 657999259 |  | 0.00 | 1.25 | 2 | 1242.60 | 0.51 | 40.08 | 0 |
| 1034 | gi\|658000539 | Ubiquitin- conjugating enzyme E2 27 |  | *Malus domestica* | Cytosol | 06.13-Protein destination and storage/Proteolys  is | 6.11 | 19.49 | 2 | 1 | 3 | 3 | 195 | 21.3 | 5.10 | High | DYQTFVGTAR | 1 | 2 | 1 | 658000539 |  | 0.00 | 2.23 | 2 | 1157.56 | -0.39 | 41.45 | 0 |
|  |  |  |  |  |  |  |  |  |  |  |  |  |  |  |  | Low | LVEMGFPEAQAR | 1 | 2 | 1 | 658000539 | M4(Oxidation) | 0.00 | 1.98 | 2 | 1363.67 | 0.88 | 40.08 | 0 |
|  |  |  |  |  |  |  |  |  |  |  |  |  |  |  |  | Low | STLEAAGGDENLA  LEK | 1 | 1 | 1 | 658000539 |  | 0.00 | 1.89 | 2 | 1617.80 | 0.28 | 40.81 | 0 |
| 1038 | gi\|658008681 | MLP-like protein 329 |  | *Malus domestica* | Cytoplasm | 11.02-  Disease/Defense  /Defense-related | 29.90 | 39.24 | 1 | 1 | 8 | 21 | 158 | 17.8 | 5.40 | High | YTIDGNVETYK | 2 | 2 | 2 | 658008685;65  8008681 |  | 0.00 | 2.95 | 2 | 1302.62 | 0.76 | 40.71 | 0 |
|  |  |  |  |  |  |  |  |  |  |  |  |  |  |  |  | High | LETMEAEVEIK | 3 | 1 | 1 | 658008681 | M4(Oxidation) | 0.00 | 2.87 | 2 | 1307.64 | 0.62 | 40.47 | 0 |
|  |  |  |  |  |  |  |  |  |  |  |  |  |  |  |  | High | DVDAHLIKE | 2 | 2 | 2 | 658008685;65  8008681 |  | 0.00 | 2.81 | 2 | 1039.54 | -0.57 | 39.28 | 1 |
|  |  |  |  |  |  |  |  |  |  |  |  |  |  |  |  | Low | YTIDGNVETYKEK | 3 | 2 | 2 | 658008685;65  8008681 |  | 0.00 | 2.36 | 2 | 1559.76 | 1.03 | 39.42 | 1 |
|  |  |  |  |  |  |  |  |  |  |  |  |  |  |  |  | Low | DVDAHLIK | 5 | 2 | 2 | 658008685;65  8008681 |  | 0.00 | 1.74 | 2 | 910.50 | -0.12 | 40.56 | 0 |

|  |  |  |  |  |  |  |  |  |  |  |  |  |  |  |  | Low | IIFQVTPNSEGGSV  K | 1 | 1 | 1 | 658008681 | N8(Deamidated) | 0.00 | 1.61 | 2 | 1576.82 | 0.95 | 44.34 | 0 |
| --- | --- | --- | --- | --- | --- | --- | --- | --- | --- | --- | --- | --- | --- | --- | --- | --- | --- | --- | --- | --- | --- | --- | --- | --- | --- | --- | --- | --- | --- |
|  |  |  |  |  |  |  |  |  |  |  |  |  |  |  |  | Low | ITLEYK | 2 | 1 | 1 | 658008681 |  | 0.00 | 1.41 | 2 | 766.43 | -0.07 | 39.80 | 0 |
|  |  |  |  |  |  |  |  |  |  |  |  |  |  |  |  | Low | IIFQVTPNSEGGSV  K | 2 | 1 | 1 | 658008681 |  | 0.00 | 1.30 | 2 | 1575.84 | 1.11 | 42.79 | 0 |
|  |  |  |  |  |  |  |  |  |  |  |  |  |  |  |  | Low | EKVEIDEANK | 1 | 1 | 1 | 658008681 |  | 0.02 | 0.50 | 2 | 1174.60 | 0.47 | 36.22 | 1 |
| 1039 | gi\|657946863 | Peroxiredoxin |  | *Malus domestica* | Plastid | 11.06-  Disease/Defense  /Detoxification | 21.58 | 19.75 | 1 | 2 | 3 | 16 | 162 | 17.4 | 5.41 | High | DVLFLADGSAK | 3 | 1 | 1 | 657946863 |  | 0.00 | 3.28 | 2 | 1135.60 | -0.02 | 46.35 | 0 |
|  |  |  |  |  |  |  |  |  |  |  |  |  |  |  |  | High | VQSVSVHSLAAGK | 6 | 1 | 1 | 657946863 |  | 0.00 | 2.72 | 2 | 1282.71 | 0.60 | 39.47 | 0 |
|  |  |  |  |  |  |  |  |  |  |  |  |  |  |  |  | Low | HVPGFIEK | 7 | 1 | 1 | 657946863 |  | 0.00 | 1.59 | 2 | 926.51 | -0.09 | 45.25 | 0 |
| 1039 | gi\|658000239 | Glutathione peroxidase |  | *Malus domestica* | Nuclear | 11.06-  Disease/Defense  /Detoxification | 5.68 | 24.54 | 9 | 1 | 4 | 6 | 163 | 18.6 | 5.00 | High | GNDVDLSLYK | 2 | 9 | 1 | 658000239 |  | 0.00 | 2.20 | 2 | 1123.56 | 0.45 | 42.70 | 0 |
|  |  |  |  |  |  |  |  |  |  |  |  |  |  |  |  | Low | EIEDFVCTR | 2 | 9 | 1 | 658000239 | C7(Carbamidomethyl) | 0.00 | 1.64 | 2 | 1168.53 | 0.08 | 42.06 | 0 |
|  |  |  |  |  |  |  |  |  |  |  |  |  |  |  |  | Low | IEVNGDNTAPVYK | 1 | 2 | 1 | 658000239 |  | 0.00 | 1.30 | 2 | 1419.71 | 1.19 | 38.92 | 0 |
|  |  |  |  |  |  |  |  |  |  |  |  |  |  |  |  | Low | SEFPIFDK | 1 | 4 | 1 | 658000239 |  | 0.00 | 1.16 | 2 | 982.49 | -0.23 | 45.57 | 0 |
| 1130 | gi\|658023330 | UMP-CMP kinase |  | *Malus domestica* | Chloroplast | 01.03-  Metabolism/Nuc leotides | 9.31 | 23.11 | 2 | 2 | 6 | 11 | 212 | 23.6 | 5.21 | High | SGSENGTMISDMI K | 2 | 2 | 1 | 658023330 | M8(Oxidation); M12(Oxidation) | 0.00 | 2.65 | 2 | 1501.65 | 1.11 | 38.75 | 0 |
|  |  |  |  |  |  |  |  |  |  |  |  |  |  |  |  | High | AAFEDVTK | 2 | 2 | 1 | 658023330 |  | 0.00 | 2.30 | 2 | 880.44 | 0.24 | 39.65 | 0 |
|  |  |  |  |  |  |  |  |  |  |  |  |  |  |  |  | Low | SGSENGTMISDMI K | 1 | 2 | 1 | 658023330 | N5(Deamidated);  M8(Oxidation); M12(Oxidation) | 0.00 | 1.81 | 2 | 1502.63 | -1.17 | 39.10 | 0 |
|  |  |  |  |  |  |  |  |  |  |  |  |  |  |  |  | Low | NQGREDDNIETIR | 2 | 3 | 1 | 658023330 |  | 0.00 | 1.40 | 2 | 1559.74 | 0.93 | 38.45 | 1 |
|  |  |  |  |  |  |  |  |  |  |  |  |  |  |  |  | Low | EDDNIETIR | 1 | 3 | 1 | 658023330 |  | 0.00 | 1.39 | 2 | 1104.52 | 0.07 | 39.85 | 0 |
|  |  |  |  |  |  |  |  |  |  |  |  |  |  |  |  | Low | FLIDGFPR | 2 | 3 | 1 | 658023330 |  | 0.00 | 1.35 | 2 | 964.53 | 0.13 | 47.39 | 0 |
|  |  |  |  |  |  |  |  |  |  |  |  |  |  |  |  | Low | AIFAPK | 1 | 2 | 1 | 658023330 |  | 0.00 | 1.05 | 2 | 646.39 | 0.20 | 39.74 | 0 |
| 1130 | gi\|657959671 | Late embryogenesis abundant protein | 99% to Prunus mume gi\|645237380 Evalue: 2e-79 | *Malus domestica* | Cell wall | 11.05-  Disease/Defense  /Stress responses | 6.70 | 5.00 | 1 | 1 | 2 | 4 | 240 | 26.2 | 5.33 | High | KTEDAVGTLAEK | 2 | 1 | 1 | 657959671 |  | 0.00 | 3.15 | 2 | 1261.66 | 0.68 | 38.29 | 1 |
|  |  |  |  |  |  |  |  |  |  |  |  |  |  |  |  | Low | TEDAVGTLAEK | 2 | 1 | 1 | 657959671 |  | 0.00 | 1.69 | 2 | 1133.57 | 0.33 | 38.46 | 0 |
| 1130 | gi\|657962934 | Thioredoxin domain-containing  protein |  | *Malus domestica* | Nucleus | 02.20-  Energy/Electron- transport | 5.91 | 26.32 | 1 | 1 | 6 | 8 | 209 | 23.9 | 5.45 | High | VDDDDLEALR | 1 | 1 | 1 | 657962934 |  | 0.00 | 2.53 | 2 | 1160.54 | 0.61 | 42.14 | 0 |
|  |  |  |  |  |  |  |  |  |  |  |  |  |  |  |  | Low | AQVIFYEGESSMK | 1 | 1 | 1 | 657962934 | M12(Oxidation) | 0.00 | 1.61 | 2 | 1504.70 | 1.31 | 41.35 | 0 |
|  |  |  |  |  |  |  |  |  |  |  |  |  |  |  |  | Low | SPFLAEK | 2 | 1 | 1 | 657962934 |  | 0.00 | 1.60 | 2 | 791.43 | 0.20 | 40.93 | 0 |
|  |  |  |  |  |  |  |  |  |  |  |  |  |  |  |  | Low | AVQEILEK | 1 | 1 | 1 | 657962934 |  | 0.00 | 1.48 | 2 | 929.53 | -0.41 | 39.42 | 0 |
|  |  |  |  |  |  |  |  |  |  |  |  |  |  |  |  | Low | LDEEIAALDR | 2 | 1 | 1 | 657962934 |  | 0.00 | 1.33 | 2 | 1144.59 | 0.97 | 42.24 | 0 |
|  |  |  |  |  |  |  |  |  |  |  |  |  |  |  |  | Low | VVCHFYR | 1 | 1 | 1 | 657962934 | C3(Carbamidomethyl) | 0.00 | 0.87 | 3 | 980.48 | 0.20 | 37.77 | 0 |
| 1624 | gi\|658008993 | Serine/threonine- protein  phosphatase |  | *Malus domestica* | Unclear | 10.04-Signal transduction/Kin  ases | 19.06 | 11.24 | 5 | 3 | 7 | 18 | 587 | 65.5 | 5.21 | High | FAATVEPAHLK | 5 | 3 | 1 | 658008993 |  | 0.00 | 2.89 | 2 | 1183.65 | 0.09 | 39.50 | 0 |
|  |  |  |  |  |  |  |  |  |  |  |  |  |  |  |  | High | LLRDNEAEVR | 6 | 5 | 1 | 658008993 |  | 0.00 | 2.60 | 3 | 1214.65 | 0.10 | 38.59 | 1 |
|  |  |  |  |  |  |  |  |  |  |  |  |  |  |  |  | High | NDDIQLR | 1 | 3 | 1 | 658008993 |  | 0.00 | 2.18 | 2 | 873.44 | -0.23 | 39.55 | 0 |
|  |  |  |  |  |  |  |  |  |  |  |  |  |  |  |  | Low | TDLVPAYVR | 2 | 3 | 1 | 658008993 |  | 0.00 | 1.96 | 2 | 1033.57 | -0.51 | 43.43 | 0 |
|  |  |  |  |  |  |  |  |  |  |  |  |  |  |  |  | Low | LLAVEGCAALGK | 1 | 4 | 1 | 658008993 | C7(Carbamidomethyl) | 0.00 | 1.39 | 2 | 1201.66 | 0.56 | 42.05 | 0 |
|  |  |  |  |  |  |  |  |  |  |  |  |  |  |  |  | Low | LLPVVVNAAK | 2 | 2 | 1 | 658008993 |  | 0.00 | 1.37 | 2 | 1023.66 | -0.21 | 43.75 | 0 |
|  |  |  |  |  |  |  |  |  |  |  |  |  |  |  |  | Low | DEFPDVR | 1 | 5 | 1 | 658008993 |  | 0.00 | 1.36 | 2 | 877.40 | -0.55 | 41.36 | 0 |
| 2113 | gi\|657997261 | Stem-specific  protein |  | *Malus domestica* | Chloroplast | 12-Unclear  classification | 26.59 | 30.80 | 2 | 2 | 8 | 15 | 250 | 26.9 | 6.40 | High | SPEALQSPQSGSAS  ALK | 3 | 1 | 1 | 657997261 |  | 0.00 | 3.75 | 2 | 1657.84 | 1.58 | 39.76 | 0 |
|  |  |  |  |  |  |  |  |  |  |  |  |  |  |  |  | High | DRGPYPADQVVR | 2 | 2 | 1 | 657997261 |  | 0.00 | 3.13 | 3 | 1372.70 | -0.43 | 38.72 | 1 |
|  |  |  |  |  |  |  |  |  |  |  |  |  |  |  |  | Low | GCFFTSSGGLR | 1 | 2 | 1 | 657997261 | C2(Carbamidomethyl) | 0.00 | 2.55 | 2 | 1188.55 | 0.41 | 42.76 | 0 |
|  |  |  |  |  |  |  |  |  |  |  |  |  |  |  |  | Low | GPYPADQVVR | 2 | 2 | 1 | 657997261 |  | 0.00 | 2.27 | 2 | 1101.57 | 0.10 | 39.93 | 0 |
|  |  |  |  |  |  |  |  |  |  |  |  |  |  |  |  | Low | GCGNSFAPFPK | 1 | 2 | 1 | 657997261 | C2(Carbamidomethyl) | 0.00 | 2.20 | 2 | 1181.54 | 0.48 | 41.93 | 0 |
|  |  |  |  |  |  |  |  |  |  |  |  |  |  |  |  | Low | GPYPADQVVR | 1 | 2 | 1 | 657997261 | Q7(Deamidated) | 0.00 | 2.16 | 2 | 1102.56 | 2.20 | 40.11 | 0 |
|  |  |  |  |  |  |  |  |  |  |  |  |  |  |  |  | Low | DGFLAR | 2 | 1 | 1 | 657997261 |  | 0.00 | 1.83 | 2 | 678.36 | 0.34 | 41.23 | 0 |
|  |  |  |  |  |  |  |  |  |  |  |  |  |  |  |  | Low | VDSSGEMCGANF  K | 1 | 2 | 1 | 657997261 | M7(Oxidation);  C8(Carbamidomethyl) | 0.00 | 1.40 | 2 | 1417.57 | 0.94 | 37.05 | 0 |
|  |  |  |  |  |  |  |  |  |  |  |  |  |  |  |  | Low | QNPLLPR | 1 | 2 | 1 | 657997261 |  | 0.00 | 1.26 | 2 | 837.49 | 0.48 | 41.00 | 0 |
|  |  |  |  |  |  |  |  |  |  |  |  |  |  |  |  | Low | VDSSGEMCGANF  K | 1 | 2 | 1 | 657997261 | C8(Carbamidomethyl) | 0.00 | 0.82 | 2 | 1401.58 | 0.98 | 39.11 | 0 |
| 2631 | gi\|658021595 | Chaperonin |  | *Malus domestica* | Chloroplast | 06.01-Protein destination and storage/Folding  and Stabillity | 33.65 | 34.43 | 3 | 1 | 22 | 32 | 575 | 61.1 | 6.15 | High | NVVLEQSFGAPK | 2 | 3 | 1 | 658021595 |  | 0.00 | 3.16 | 2 | 1288.69 | 0.74 | 42.91 | 0 |
|  |  |  |  |  |  |  |  |  |  |  |  |  |  |  |  | High | NVVLEQSFGAPK | 2 | 3 | 1 | 658021595 | Q6(Deamidated) | 0.00 | 2.69 | 2 | 1289.67 | 1.02 | 43.20 | 0 |
|  |  |  |  |  |  |  |  |  |  |  |  |  |  |  |  | Low | VGIETLGTCK | 1 | 2 | 1 | 658021595 | C9(Carbamidomethyl) | 0.00 | 2.35 | 2 | 1077.56 | 0.15 | 40.81 | 0 |
|  |  |  |  |  |  |  |  |  |  |  |  |  |  |  |  | Low | IGGASEAEVSEK | 1 | 2 | 1 | 658021595 |  | 0.00 | 2.32 | 2 | 1176.57 | 0.25 | 37.55 | 0 |
|  |  |  |  |  |  |  |  |  |  |  |  |  |  |  |  | Low | ITVSKDDTVILDG  AGDKK | 1 | 3 | 1 | 658021595 |  | 0.00 | 2.18 | 3 | 1875.01 | 0.23 | 40.68 | 2 |

|  |  |  |  |  |  |  |  |  |  |  |  |  |  |  |  | Low | GVEELADAVK | 1 | 3 | 1 | 658021595 |  | 0.00 | 2.17 | 2 | 1030.54 | 0.33 | 42.04 | 0 |
| --- | --- | --- | --- | --- | --- | --- | --- | --- | --- | --- | --- | --- | --- | --- | --- | --- | --- | --- | --- | --- | --- | --- | --- | --- | --- | --- | --- | --- | --- |
|  |  |  |  |  |  |  |  |  |  |  |  |  |  |  |  | Low | SVAAGMNAMDLR | 2 | 3 | 1 | 658021595 | M9(Oxidation) | 0.00 | 2.10 | 2 | 1251.58 | 0.47 | 39.49 | 0 |
|  |  |  |  |  |  |  |  |  |  |  |  |  |  |  |  | Low | EIGELIAK | 1 | 3 | 1 | 658021595 |  | 0.01 | 1.96 | 2 | 872.51 | -0.04 | 41.79 | 0 |
|  |  |  |  |  |  |  |  |  |  |  |  |  |  |  |  | Low | AIFTEGCK | 1 | 3 | 1 | 658021595 | C7(Carbamidomethyl) | 0.00 | 1.94 | 2 | 925.44 | -0.15 | 39.69 | 0 |
|  |  |  |  |  |  |  |  |  |  |  |  |  |  |  |  | Low | EGVITISDGK | 1 | 2 | 1 | 658021595 |  | 0.00 | 1.92 | 2 | 1018.54 | -0.32 | 40.38 | 0 |
|  |  |  |  |  |  |  |  |  |  |  |  |  |  |  |  | Low | SVAAGMNAMDLR | 1 | 3 | 1 | 658021595 |  | 0.00 | 1.89 | 2 | 1235.59 | 0.60 | 42.05 | 0 |
|  |  |  |  |  |  |  |  |  |  |  |  |  |  |  |  | Low | AGIIDPLK | 2 | 3 | 1 | 658021595 |  | 0.00 | 1.82 | 2 | 826.50 | 0.01 | 44.00 | 0 |
|  |  |  |  |  |  |  |  |  |  |  |  |  |  |  |  | Low | LSNLNSIVK | 1 | 3 | 1 | 658021595 |  | 0.00 | 1.79 | 2 | 987.58 | -0.12 | 41.92 | 0 |
|  |  |  |  |  |  |  |  |  |  |  |  |  |  |  |  | Low | SSIELSTSDYDK | 1 | 3 | 1 | 658021595 |  | 0.00 | 1.74 | 2 | 1344.62 | 0.86 | 40.68 | 0 |
|  |  |  |  |  |  |  |  |  |  |  |  |  |  |  |  | Low | GEYVDMIK | 1 | 3 | 1 | 658021595 | M6(Oxidation) | 0.00 | 1.61 | 2 | 970.45 | -0.56 | 38.69 | 0 |
|  |  |  |  |  |  |  |  |  |  |  |  |  |  |  |  | Low | GYISPYFITNPK | 1 | 3 | 1 | 658021595 |  | 0.00 | 1.47 | 2 | 1399.73 | 0.33 | 44.98 | 0 |
|  |  |  |  |  |  |  |  |  |  |  |  |  |  |  |  | Low | FGVEAR | 1 | 3 | 1 | 658021595 |  | 0.00 | 1.44 | 2 | 678.36 | -0.01 | 38.61 | 0 |
|  |  |  |  |  |  |  |  |  |  |  |  |  |  |  |  | Low | ILELALQK | 1 | 3 | 1 | 658021595 | Q7(Deamidated) | 0.00 | 1.25 | 2 | 928.57 | 1.04 | 45.11 | 0 |
|  |  |  |  |  |  |  |  |  |  |  |  |  |  |  |  | Low | LLEQDNPDLGYD  AAK | 1 | 3 | 1 | 658021595 |  | 0.00 | 1.22 | 2 | 1661.80 | 1.56 | 41.41 | 0 |
|  |  |  |  |  |  |  |  |  |  |  |  |  |  |  |  | Low | VTDALNATK | 1 | 3 | 1 | 658021595 |  | 0.00 | 1.16 | 2 | 932.50 | 0.16 | 37.23 | 0 |
|  |  |  |  |  |  |  |  |  |  |  |  |  |  |  |  | Low | SSIELSTSDYDKEK | 1 | 3 | 1 | 658021595 |  | 0.00 | 1.13 | 2 | 1601.76 | 0.72 | 38.97 | 1 |
|  |  |  |  |  |  |  |  |  |  |  |  |  |  |  |  | Low | NIGASLVK | 1 | 3 | 1 | 658021595 |  | 0.00 | 1.04 | 2 | 801.48 | -0.11 | 39.88 | 0 |
|  |  |  |  |  |  |  |  |  |  |  |  |  |  |  |  | Low | ILELALQK | 1 | 3 | 1 | 658021595 |  | 0.00 | 1.00 | 2 | 927.59 | -0.20 | 43.71 | 0 |
|  |  |  |  |  |  |  |  |  |  |  |  |  |  |  |  | Low | DDTVILDGAGDK  K | 1 | 3 | 1 | 658021595 |  | 0.06 | 0.97 | 2 | 1346.68 | 0.62 | 39.52 | 1 |
|  |  |  |  |  |  |  |  |  |  |  |  |  |  |  |  | Low | IGGASEAEVSEKK | 2 | 2 | 1 | 658021595 |  | 0.00 | 0.87 | 2 | 1304.67 | 1.63 | 36.71 | 1 |
|  |  |  |  |  |  |  |  |  |  |  |  |  |  |  |  | Low | SVAAGMNAMDLR | 2 | 3 | 1 | 658021595 | M6(Oxidation);  M9(Oxidation) | 0.00 | 0.66 | 2 | 1267.58 | 1.29 | 36.99 | 0 |
| 3211 | gi\|657960827 | Glyceraldehyde-3- phosphate dehydrogenase |  | *Malus domestica* | Chloroplast | 11.05-  Disease/Defense  /Stress responses | 6.96 | 9.60 | 2 | 1 | 4 | 5 | 427 | 45.1 | 7.74 | High | AGLALSSSFVK | 1 | 2 | 1 | 657960827 |  | 0.00 | 2.82 | 2 | 1079.61 | 0.30 | 43.89 | 0 |
|  |  |  |  |  |  |  |  |  |  |  |  |  |  |  |  | Low | YAADGPLR | 1 | 2 | 1 | 657960827 |  | 0.00 | 2.14 | 2 | 862.44 | -0.17 | 38.63 | 0 |
|  |  |  |  |  |  |  |  |  |  |  |  |  |  |  |  | Low | GAGQNIIPSSTGAA  K | 1 | 2 | 1 | 657960827 |  | 0.00 | 2.00 | 2 | 1371.72 | 0.86 | 39.04 | 0 |
|  |  |  |  |  |  |  |  |  |  |  |  |  |  |  |  | Low | LTGMAFR | 1 | 2 | 1 | 657960827 | M4(Oxidation) | 0.00 | 1.22 | 2 | 811.41 | -0.30 | 38.40 | 0 |
|  |  |  |  |  |  |  |  |  |  |  |  |  |  |  |  | Low | LTGMAFR | 1 | 2 | 1 | 657960827 |  | 0.05 | 0.83 | 2 | 795.42 | -0.26 | 41.51 | 0 |
| 3322 | gi\|658007299 | L-3-cyanoalanine synthase |  | *Malus domestica* | Mitochondrion | 01.01-  Metabolism/Ami no Acid | 24.97 | 18.62 | 1 | 3 | 6 | 10 | 376 | 40.9 | 7.77 | High | DRPALSMINDAEE K | 1 | 3 | 2 | 658007299;65  8016130 | M7(Oxidation) | 0.00 | 4.28 | 3 | 1604.76 | -0.06 | 36.50 | 0 |
|  |  |  |  |  |  |  |  |  |  |  |  |  |  |  |  | High | VIEVTSEDAVNMA  R | 2 | 1 | 1 | 658007299 | M12(Oxidation) | 0.00 | 3.63 | 2 | 1549.76 | 2.20 | 37.65 | 0 |
|  |  |  |  |  |  |  |  |  |  |  |  |  |  |  |  | High | VTEGCGAYIAVK | 2 | 1 | 1 | 658007299 | C5(Carbamidomethyl) | 0.00 | 2.94 | 2 | 1267.64 | 0.50 | 37.08 | 0 |
|  |  |  |  |  |  |  |  |  |  |  |  |  |  |  |  | High | TQVSQLIGR | 2 | 3 | 2 | 658007299;65  8016130 | Q5(Deamidated) | 0.00 | 2.91 | 2 | 1002.56 | 0.64 | 39.05 | 0 |
|  |  |  |  |  |  |  |  |  |  |  |  |  |  |  |  | Medium | TQVSQLIGR | 1 | 3 | 2 | 658007299;65  8016130 |  | 0.00 | 2.50 | 2 | 1001.57 | -0.27 | 38.22 | 0 |
|  |  |  |  |  |  |  |  |  |  |  |  |  |  |  |  | Medium | TPIVYLNK | 1 | 3 | 2 | 658007299;65  8016130 |  | 0.00 | 2.24 | 2 | 947.56 | 0.01 | 38.37 | 0 |
|  |  |  |  |  |  |  |  |  |  |  |  |  |  |  |  | Medium | MVLTMPSYTSLER | 1 | 1 | 1 | 658007299 | M1(Oxidation);  M5(Oxidation) | 0.00 | 2.22 | 2 | 1559.75 | 1.78 | 39.20 | 0 |
| 3322 | gi\|658005391 | Methylthioribose-1 phosphate  isomerase |  | *Malus domestica* | Chloroplast | 01.01-  Metabolism/Ami no Acid | 21.39 | 16.49 | 1 | 4 | 6 | 9 | 382 | 40.5 | 5.80 | High | GSLQLLDQTK | 2 | 2 | 2 | 658005391;65  7947312 | Q8(Deamidated) | 0.00 | 3.35 | 2 | 1103.60 | 1.84 | 40.19 | 0 |
|  |  |  |  |  |  |  |  |  |  |  |  |  |  |  |  | High | QIASAVATTTSDA  R | 1 | 1 | 1 | 658005391 |  | 0.00 | 3.04 | 2 | 1391.71 | 1.32 | 35.85 | 0 |
|  |  |  |  |  |  |  |  |  |  |  |  |  |  |  |  | High | VNAVVVGAYR | 1 | 1 | 1 | 658005391 |  | 0.00 | 2.75 | 2 | 1047.59 | -0.18 | 37.65 | 0 |
|  |  |  |  |  |  |  |  |  |  |  |  |  |  |  |  | High | QIASAVATTTSDA  R | 1 | 1 | 1 | 658005391 | Q1(Deamidated) | 0.00 | 2.72 | 2 | 1392.70 | 1.23 | 36.91 | 0 |
|  |  |  |  |  |  |  |  |  |  |  |  |  |  |  |  | High | SLHTEGVLER | 1 | 2 | 2 | 658005391;65  7947312 |  | 0.00 | 2.62 | 3 | 1140.60 | -0.14 | 35.33 | 0 |
|  |  |  |  |  |  |  |  |  |  |  |  |  |  |  |  | Medium | GSLQLLDQTK | 1 | 2 | 2 | 658005391;65  7947312 |  | 0.00 | 2.45 | 2 | 1102.61 | 0.08 | 39.49 | 0 |
|  |  |  |  |  |  |  |  |  |  |  |  |  |  |  |  | Medium | GTDAFDIK | 1 | 1 | 1 | 658005391 |  | 0.00 | 2.26 | 2 | 866.43 | -0.08 | 38.55 | 0 |
|  |  |  |  |  |  |  |  |  |  |  |  |  |  |  |  | Medium | IGTYNLAVSAK | 1 | 1 | 1 | 658005391 |  | 0.00 | 2.19 | 2 | 1136.63 | -0.33 | 38.32 | 0 |
| 3322 | gi\|658063654 | Leucine aminopeptidase |  | *Malus domestica* | Chloroplast | 06.13-Protein destination and storage/Proteolys  is | 10.00 | 8.90 | 6 | 4 | 4 | 4 | 573 | 59.8 | 6.80 | High | FDMGGSAAVLGA AK | 1 | 4 | 1 | 658063654 | M3(Oxidation) | 0.00 | 2.94 | 2 | 1310.64 | 0.40 | 38.49 | 0 |
|  |  |  |  |  |  |  |  |  |  |  |  |  |  |  |  | Medium | GLGEAAAAAAK | 1 | 1 | 1 | 658063654 |  | 0.00 | 2.37 | 2 | 929.50 | -0.16 | 35.70 | 0 |
|  |  |  |  |  |  |  |  |  |  |  |  |  |  |  |  | Medium | ELVNSPANVLTPG  K | 1 | 4 | 1 | 658063654 |  | 0.00 | 2.36 | 2 | 1438.79 | 0.76 | 40.42 | 0 |
|  |  |  |  |  |  |  |  |  |  |  |  |  |  |  |  | Medium | TIEVNNTDAEGR | 1 | 5 | 1 | 658063654 |  | 0.00 | 2.34 | 2 | 1318.63 | 1.52 | 35.14 | 0 |
| 3322 | gi\|658009013 | Leucoanthocyanidi n reductase |  | *Malus domestica* | Cytoplasm | 20.2-Secondary  metabolism/Terp enoids | 8.96 | 12.99 | 2 | 4 | 4 | 4 | 354 | 38.9 | 5.58 | Medium | VSTENTVVESR | 1 | 1 | 1 | 658009013 |  | 0.00 | 2.40 | 2 | 1220.61 | 0.58 | 34.55 | 0 |

|  |  |  |  |  |  |  |  |  |  |  |  |  |  |  |  | Medium | VLIAGATGFIGR | 1 | 1 | 1 | 658009013 |  | 0.00 | 2.38 | 2 | 1174.69 | 0.24 | 42.10 | 0 |
| --- | --- | --- | --- | --- | --- | --- | --- | --- | --- | --- | --- | --- | --- | --- | --- | --- | --- | --- | --- | --- | --- | --- | --- | --- | --- | --- | --- | --- | --- |
|  |  |  |  |  |  |  |  |  |  |  |  |  |  |  |  | Medium | FLPSEFGHDVDR | 1 | 2 | 1 | 658009013 |  | 0.00 | 2.14 | 2 | 1418.67 | 0.77 | 39.10 | 0 |
|  |  |  |  |  |  |  |  |  |  |  |  |  |  |  |  | Medium | AYFVDGTDIGK | 1 | 2 | 1 | 658009013 |  | 0.00 | 2.04 | 2 | 1185.58 | 0.39 | 40.45 | 0 |
| 3325 | gi\|658050324 | Alpha-1,4-glucan- protein synthase |  | *Malus domestica* | Chloroplast | 09.01-Cell structure/Cell  wall | 34.30 | 28.73 | 7 | 6 | 11 | 22 | 362 | 41.1 | 5.97 | High | FVDAVLTIPK | 2 | 5 | 1 | 658050324 |  | 0.00 | 3.30 | 2 | 1102.65 | 0.07 | 44.61 | 0 |
|  |  |  |  |  |  |  |  |  |  |  |  |  |  |  |  | High | AVEANGVVAK | 1 | 1 | 1 | 658050324 | N5(Deamidated) | 0.00 | 2.75 | 2 | 958.52 | -0.21 | 37.36 | 0 |
|  |  |  |  |  |  |  |  |  |  |  |  |  |  |  |  | High | DINALEQHIK | 3 | 4 | 1 | 658050324 |  | 0.00 | 2.58 | 2 | 1180.63 | 0.84 | 41.70 | 0 |
|  |  |  |  |  |  |  |  |  |  |  |  |  |  |  |  | High | DCTTVQGCYLELS  K | 1 | 3 | 1 | 658050324 | C2(Carbamidomethyl);  C8(Carbamidomethyl) | 0.00 | 2.48 | 2 | 1673.75 | 1.70 | 42.37 | 0 |
|  |  |  |  |  |  |  |  |  |  |  |  |  |  |  |  | High | VICDHLGLGVK | 5 | 4 | 1 | 658050324 | C3(Carbamidomethyl) | 0.00 | 2.44 | 2 | 1210.66 | 0.39 | 42.11 | 0 |
|  |  |  |  |  |  |  |  |  |  |  |  |  |  |  |  | High | VDDYFIK | 2 | 1 | 1 | 658050324 |  | 0.00 | 2.31 | 2 | 899.45 | -0.57 | 42.91 | 0 |
|  |  |  |  |  |  |  |  |  |  |  |  |  |  |  |  | Low | ASNPFVNLK | 2 | 4 | 1 | 658050324 |  | 0.00 | 1.94 | 2 | 989.54 | 0.05 | 43.03 | 0 |
|  |  |  |  |  |  |  |  |  |  |  |  |  |  |  |  | Low | ASCISFK | 2 | 3 | 1 | 658050324 | C3(Carbamidomethyl) | 0.00 | 1.80 | 2 | 812.40 | 0.76 | 39.68 | 0 |
|  |  |  |  |  |  |  |  |  |  |  |  |  |  |  |  | Low | VPEGFDYELYNR | 1 | 2 | 1 | 658050324 |  | 0.00 | 1.74 | 2 | 1501.70 | 0.26 | 43.72 | 0 |
|  |  |  |  |  |  |  |  |  |  |  |  |  |  |  |  | Low | GYPFSLR | 1 | 6 | 1 | 658050324 |  | 0.00 | 1.01 | 2 | 839.44 | 0.27 | 44.14 | 0 |
|  |  |  |  |  |  |  |  |  |  |  |  |  |  |  |  | Low | EGADFVR | 2 | 4 | 1 | 658050324 |  | 0.00 | 0.82 | 2 | 793.38 | 0.02 | 40.16 | 0 |
| 3325 | gi\|657957773 | Disulfide- isomerase |  | *Malus domestica* | Endoplasmatic reticulum | 06.01-Protein destination and storage/Folding  and Stabillity | 33.75 | 25.48 | 1 | 3 | 12 | 26 | 361 | 39.7 | 5.73 | High | NAEALAEFVNK | 3 | 1 | 1 | 657957773 |  | 0.00 | 4.49 | 2 | 1205.62 | 0.00 | 43.52 | 0 |
|  |  |  |  |  |  |  |  |  |  |  |  |  |  |  |  | High | YGVSGYPTLK | 3 | 1 | 1 | 657957773 |  | 0.00 | 2.72 | 2 | 1084.57 | 0.06 | 41.59 | 0 |
|  |  |  |  |  |  |  |  |  |  |  |  |  |  |  |  | High | YGNIYLK | 1 | 1 | 1 | 657957773 |  | 0.00 | 2.64 | 2 | 870.47 | -0.23 | 40.29 | 0 |
|  |  |  |  |  |  |  |  |  |  |  |  |  |  |  |  | Low | IEEEVGKLEGSAA  R | 5 | 2 | 2 | 657957773;65  7992452 |  | 0.00 | 1.95 | 2 | 1487.77 | 1.21 | 38.66 | 1 |
|  |  |  |  |  |  |  |  |  |  |  |  |  |  |  |  | Low | GADYAKNEIQR | 3 | 1 | 1 | 657957773 |  | 0.00 | 1.94 | 2 | 1264.63 | -0.09 | 37.84 | 1 |
|  |  |  |  |  |  |  |  |  |  |  |  |  |  |  |  | Low | KNILYTFASSS | 1 | 1 | 1 | 657957773 |  | 0.00 | 1.87 | 2 | 1230.64 | 0.21 | 44.74 | 1 |
|  |  |  |  |  |  |  |  |  |  |  |  |  |  |  |  | Low | ADEFTLKK | 2 | 2 | 2 | 657957773;65  7992452 |  | 0.00 | 1.73 | 2 | 951.51 | -0.05 | 39.24 | 1 |
|  |  |  |  |  |  |  |  |  |  |  |  |  |  |  |  | Low | SILIAK | 1 | 2 | 2 | 657957773;65  7992452 |  | 0.04 | 1.33 | 2 | 644.43 | 0.22 | 40.80 | 0 |
|  |  |  |  |  |  |  |  |  |  |  |  |  |  |  |  | Low | ADEFTLK | 2 | 2 | 2 | 657957773;65  7992452 |  | 0.00 | 1.28 | 2 | 823.42 | 0.02 | 41.46 | 0 |
|  |  |  |  |  |  |  |  |  |  |  |  |  |  |  |  | Low | SLAPTYEK | 3 | 2 | 2 | 657957773;65  7992452 |  | 0.00 | 1.19 | 2 | 908.47 | -0.20 | 38.34 | 0 |
|  |  |  |  |  |  |  |  |  |  |  |  |  |  |  |  | Low | NILYTFASSS | 1 | 1 | 1 | 657957773 |  | 0.00 | 1.02 | 2 | 1102.54 | 0.34 | 46.94 | 0 |
|  |  |  |  |  |  |  |  |  |  |  |  |  |  |  |  | Low | LAPEYEK | 1 | 2 | 2 | 657957773;65  7992452 |  | 0.10 | 0.46 | 2 | 849.44 | -0.07 | 36.54 | 0 |
| 3325 | gi\|657992826 | Aldo-keto reductase |  | *Malus domestica* | Chloroplast | 11.06-  Disease/Defense  /Detoxification | 15.83 | 21.78 | 10 | 3 | 9 | 12 | 349 | 38.4 | 5.85 | High | NLDSNIDSLK | 2 | 1 | 1 | 657992826 |  | 0.00 | 2.98 | 2 | 1118.57 | -0.06 | 41.38 | 0 |
|  |  |  |  |  |  |  |  |  |  |  |  |  |  |  |  | High | YIGLSEASPDTIR | 1 | 10 | 1 | 657992826 |  | 0.00 | 2.93 | 2 | 1421.73 | 1.17 | 42.14 | 0 |
|  |  |  |  |  |  |  |  |  |  |  |  |  |  |  |  | High | VQLGNCGFEVSK | 2 | 1 | 1 | 657992826 | C6(Carbamidomethyl) | 0.00 | 2.77 | 2 | 1337.65 | 0.84 | 41.70 | 0 |
|  |  |  |  |  |  |  |  |  |  |  |  |  |  |  |  | Low | IKNLDSNIDSLK | 1 | 1 | 1 | 657992826 |  | 0.00 | 1.64 | 2 | 1359.75 | 1.32 | 40.40 | 1 |
|  |  |  |  |  |  |  |  |  |  |  |  |  |  |  |  | Low | FVGENLDK | 1 | 1 | 1 | 657992826 |  | 0.00 | 1.61 | 2 | 921.47 | 0.10 | 39.05 | 0 |
|  |  |  |  |  |  |  |  |  |  |  |  |  |  |  |  | Low | IEILGK | 1 | 1 | 1 | 657992826 |  | 0.00 | 1.50 | 2 | 672.43 | 1.05 | 41.64 | 0 |
|  |  |  |  |  |  |  |  |  |  |  |  |  |  |  |  | Low | FVGENLDKNK | 1 | 1 | 1 | 657992826 |  | 0.04 | 1.05 | 3 | 1163.61 | 0.15 | 38.23 | 1 |
|  |  |  |  |  |  |  |  |  |  |  |  |  |  |  |  | Low | GFFGGK | 2 | 3 | 1 | 657992826 |  | 0.04 | 0.86 | 2 | 612.31 | 0.22 | 41.20 | 0 |
|  |  |  |  |  |  |  |  |  |  |  |  |  |  |  |  | Low | EVVESVSANSYVA  SHPR | 1 | 1 | 1 | 657992826 |  | 0.00 | 0.78 | 2 | 1830.90 | 1.33 | 39.38 | 0 |
| 3325 | gi\|657945783 | Caffeic acid 3-O- methyltransferase |  | *Malus domestica* | Cytoplasm | 20.1-Secondary metabolism/Phen ylpropanoids/Phe  nolics | 14.24 | 15.47 | 11 | 2 | 8 | 10 | 433 | 47.1 | 6.52 | Low | TEKEFEALAK | 1 | 8 | 1 | 657945783 |  | 0.00 | 2.37 | 3 | 1165.61 | 0.57 | 39.87 | 1 |
|  |  |  |  |  |  |  |  |  |  |  |  |  |  |  |  | High | NCYAALPDNGK | 1 | 6 | 1 | 657945783 | C2(Carbamidomethyl) | 0.00 | 2.34 | 2 | 1222.55 | 0.52 | 38.22 | 0 |
|  |  |  |  |  |  |  |  |  |  |  |  |  |  |  |  | Medium | DAVLEGGIPFNK | 1 | 9 | 1 | 657945783 |  | 0.00 | 2.18 | 2 | 1259.66 | 0.70 | 43.93 | 0 |
|  |  |  |  |  |  |  |  |  |  |  |  |  |  |  |  | Low | GDAIFMK | 1 | 11 | 1 | 657945783 | M6(Oxidation) | 0.00 | 2.03 | 2 | 797.39 | 0.26 | 39.52 | 0 |
|  |  |  |  |  |  |  |  |  |  |  |  |  |  |  |  | Low | NPDAPVMLDR | 1 | 5 | 1 | 657945783 | M7(Oxidation) | 0.00 | 1.94 | 2 | 1143.55 | 0.39 | 38.89 | 0 |
|  |  |  |  |  |  |  |  |  |  |  |  |  |  |  |  | Low | GSGFQGFR | 2 | 7 | 1 | 657945783 |  | 0.00 | 1.72 | 2 | 855.41 | -0.37 | 39.92 | 0 |
|  |  |  |  |  |  |  |  |  |  |  |  |  |  |  |  | Low | LYGLGPVCK | 1 | 5 | 1 | 657945783 | C8(Carbamidomethyl) | 0.00 | 1.53 | 2 | 1006.54 | -0.19 | 41.77 | 0 |
|  |  |  |  |  |  |  |  |  |  |  |  |  |  |  |  | Low | NPDAPVMLDR | 1 | 5 | 1 | 657945783 |  | 0.09 | 1.07 | 2 | 1127.55 | -0.54 | 41.10 | 0 |
|  |  |  |  |  |  |  |  |  |  |  |  |  |  |  |  | Low | EFEALAK | 1 | 8 | 1 | 657945783 |  | 0.03 | 0.97 | 2 | 807.43 | 0.36 | 40.20 | 0 |
| 3522 | gi\|657951571 | D-3-  phosphoglycerate dehydrogenase |  | *Malus domestica* | Unclear | 01.01-  Metabolism/Ami no Acid | 16.07 | 15.55 | 3 | 4 | 10 | 14 | 598 | 62.9 | 7.77 | High | LAVQLVAGGSGV K | 1 | 1 | 1 | 657951571 |  | 0.00 | 3.10 | 2 | 1198.72 | 0.23 | 42.04 | 0 |
|  |  |  |  |  |  |  |  |  |  |  |  |  |  |  |  | High | GGVIDEDALVR | 1 | 3 | 1 | 657951571 |  | 0.00 | 3.02 | 2 | 1143.60 | 0.22 | 41.71 | 0 |
|  |  |  |  |  |  |  |  |  |  |  |  |  |  |  |  | Medium | LGEPGINLLK | 2 | 1 | 1 | 657951571 |  | 0.00 | 2.42 | 2 | 1053.63 | -1.08 | 44.54 | 0 |
|  |  |  |  |  |  |  |  |  |  |  |  |  |  |  |  | Medium | TLAVMGFGK | 2 | 2 | 1 | 657951571 | M5(Oxidation) | 0.00 | 2.13 | 2 | 939.50 | 0.00 | 41.55 | 0 |
|  |  |  |  |  |  |  |  |  |  |  |  |  |  |  |  | Low | DGVPHLTK | 2 | 1 | 1 | 657951571 |  | 0.00 | 1.89 | 2 | 866.47 | -0.44 | 38.55 | 0 |
|  |  |  |  |  |  |  |  |  |  |  |  |  |  |  |  | Low | ISLCDALIVR | 1 | 1 | 1 | 657951571 | C4(Carbamidomethyl) | 0.00 | 1.82 | 2 | 1159.65 | 0.55 | 45.71 | 0 |
|  |  |  |  |  |  |  |  |  |  |  |  |  |  |  |  | Low | VLNDDTFAK | 2 | 1 | 1 | 657951571 |  | 0.00 | 1.70 | 2 | 1022.52 | -0.10 | 39.94 | 0 |
|  |  |  |  |  |  |  |  |  |  |  |  |  |  |  |  | Low | FASAISESGEIK | 1 | 1 | 1 | 657951571 |  | 0.00 | 1.51 | 2 | 1238.63 | 0.31 | 39.95 | 0 |

|  |  |  |  |  |  |  |  |  |  |  |  |  |  |  |  | Low | VKDGVPHLTK | 1 | 1 | 1 | 657951571 |  | 0.00 | 1.35 | 2 | 1093.64 | 0.05 | 39.14 | 1 |
| --- | --- | --- | --- | --- | --- | --- | --- | --- | --- | --- | --- | --- | --- | --- | --- | --- | --- | --- | --- | --- | --- | --- | --- | --- | --- | --- | --- | --- | --- |
|  |  |  |  |  |  |  |  |  |  |  |  |  |  |  |  | Low | YVGVSLVGK | 1 | 2 | 1 | 657951571 |  | 0.00 | 1.02 | 2 | 921.54 | -0.11 | 41.27 | 0 |
| 3522 | gi\|657943380 | Phosphomannomut ase/phosphoglucom utase isoform | 100% to Prunus mume gi\|645255050 E-  value: 0.0 | *Malus domestica* | Chloroplast | 01.05-  Metabolism/Sug ars and polysaccharides | 15.46 | 14.34 | 3 | 2 | 9 | 14 | 544 | 58.3 | 5.91 | High | SAAVDSTGLEFNR | 1 | 3 | 1 | 657943380 |  | 0.00 | 3.62 | 2 | 1366.66 | 1.10 | 40.92 | 0 |
|  |  |  |  |  |  |  |  |  |  |  |  |  |  |  |  | High | AITQAVLDNK | 1 | 3 | 1 | 657943380 |  | 0.00 | 2.76 | 2 | 1072.60 | 0.03 | 39.30 | 0 |
|  |  |  |  |  |  |  |  |  |  |  |  |  |  |  |  | Low | LEDAISR | 1 | 3 | 1 | 657943380 |  | 0.00 | 1.97 | 2 | 803.43 | -0.35 | 37.26 | 0 |
|  |  |  |  |  |  |  |  |  |  |  |  |  |  |  |  | Low | NVIDEAIR | 2 | 3 | 1 | 657943380 |  | 0.00 | 1.80 | 2 | 929.51 | 0.06 | 40.87 | 0 |
|  |  |  |  |  |  |  |  |  |  |  |  |  |  |  |  | Low | HLENYIDSDPK | 3 | 3 | 1 | 657943380 |  | 0.00 | 1.65 | 2 | 1330.63 | 0.52 | 39.81 | 0 |
|  |  |  |  |  |  |  |  |  |  |  |  |  |  |  |  | Low | EFPALDTSALDK | 1 | 3 | 1 | 657943380 |  | 0.00 | 1.50 | 2 | 1306.65 | 0.50 | 44.34 | 0 |
|  |  |  |  |  |  |  |  |  |  |  |  |  |  |  |  | Low | DYGEAVLK | 1 | 3 | 1 | 657943380 |  | 0.05 | 1.39 | 2 | 894.46 | -0.12 | 39.82 | 0 |
|  |  |  |  |  |  |  |  |  |  |  |  |  |  |  |  | Low | KLEDAISR | 3 | 3 | 1 | 657943380 |  | 0.00 | 1.35 | 2 | 931.52 | -0.17 | 37.59 | 1 |
|  |  |  |  |  |  |  |  |  |  |  |  |  |  |  |  | Low | LQNGSDIR | 1 | 3 | 1 | 657943380 | Q2(Deamidated);  N3(Deamidated) | 0.09 | 1.10 | 2 | 904.44 | -0.44 | 36.90 | 0 |
| 3522 | gi\|658022678 | Betaine aldehyde dehydrogenase |  | *Malus domestica* | Chloroplast | 01.01-  Metabolism/Ami no Acid | 14.83 | 11.13 | 3 | 4 | 5 | 8 | 503 | 54.8 | 5.69 | High | IAFTGSTMTGSK | 1 | 2 | 1 | 658022678 | M8(Oxidation) | 0.00 | 2.79 | 2 | 1216.59 | 0.45 | 38.49 | 0 |
|  |  |  |  |  |  |  |  |  |  |  |  |  |  |  |  | Medium | IADPLEEGCR | 2 | 2 | 1 | 658022678 | C9(Carbamidomethyl) | 0.00 | 2.39 | 2 | 1159.54 | 0.77 | 41.06 | 0 |
|  |  |  |  |  |  |  |  |  |  |  |  |  |  |  |  | Medium | LGPVVSGGQYEK | 2 | 1 | 1 | 658022678 |  | 0.00 | 2.28 | 2 | 1233.65 | 1.25 | 39.70 | 0 |
|  |  |  |  |  |  |  |  |  |  |  |  |  |  |  |  | Medium | EEVFGPVLCVK | 2 | 3 | 1 | 658022678 | C9(Carbamidomethyl) | 0.00 | 2.06 | 2 | 1276.66 | 0.60 | 44.61 | 0 |
|  |  |  |  |  |  |  |  |  |  |  |  |  |  |  |  | Low | APISLPMEQFK | 1 | 1 | 1 | 658022678 | M7(Oxidation) | 0.00 | 1.65 | 2 | 1276.66 | 1.37 | 42.39 | 0 |
| 3522 | gi\|657970680 | T-complex protein |  | *Malus domestica* | Cytoplasm | 06.01-Protein destination and storage/Folding  and Stabillity | 14.49 | 12.33 | 2 | 1 | 7 | 12 | 527 | 57.0 | 5.88 | High | VDEIITCAPR | 2 | 2 | 1 | 657970680 | C7(Carbamidomethyl) | 0.00 | 3.12 | 2 | 1173.59 | 0.53 | 39.79 | 0 |
|  |  |  |  |  |  |  |  |  |  |  |  |  |  |  |  | Low | GSTNLEAIQIIK | 2 | 2 | 1 | 657970680 |  | 0.00 | 1.96 | 2 | 1286.73 | 0.75 | 44.28 | 0 |
|  |  |  |  |  |  |  |  |  |  |  |  |  |  |  |  | Low | SHAIEAFSR | 3 | 2 | 1 | 657970680 |  | 0.00 | 1.92 | 2 | 1017.51 | -0.28 | 40.22 | 0 |
|  |  |  |  |  |  |  |  |  |  |  |  |  |  |  |  | Low | IFKDEASEEKGER | 1 | 2 | 1 | 657970680 |  | 0.01 | 1.91 | 3 | 1537.75 | -0.24 | 38.76 | 2 |
|  |  |  |  |  |  |  |  |  |  |  |  |  |  |  |  | Low | IGLGQPK | 1 | 2 | 1 | 657970680 |  | 0.00 | 1.59 | 2 | 712.44 | 0.15 | 37.64 | 0 |
|  |  |  |  |  |  |  |  |  |  |  |  |  |  |  |  | Low | VLVDISK | 1 | 2 | 1 | 657970680 |  | 0.00 | 1.39 | 2 | 773.48 | -0.05 | 39.97 | 0 |
|  |  |  |  |  |  |  |  |  |  |  |  |  |  |  |  | Low | GISEAFK | 1 | 2 | 1 | 657970680 |  | 0.00 | 1.08 | 2 | 751.40 | 0.28 | 39.13 | 0 |
|  |  |  |  |  |  |  |  |  |  |  |  |  |  |  |  | Low | GISEAFK | 1 | 2 | 1 | 657970680 | N-Term(Acetyl) | 0.00 | 0.51 | 2 | 793.41 | 1.64 | 41.28 | 0 |
| 3522 | gi\|658007487 | Glucose-6- phosphate  isomerase |  | *Malus domestica* | Cytosol | 02.01-  Energy/Glycolys is | 10.89 | 12.78 | 3 | 3 | 9 | 11 | 618 | 67.4 | 6.25 | High | AIIAEGNCGSPR | 1 | 3 | 1 | 658007487 | C8(Carbamidomethyl) | 0.00 | 2.93 | 2 | 1244.61 | 0.59 | 37.48 | 0 |
|  |  |  |  |  |  |  |  |  |  |  |  |  |  |  |  | High | VNQGLTVYGNK | 2 | 3 | 1 | 658007487 |  | 0.00 | 2.48 | 2 | 1192.63 | 0.43 | 39.36 | 0 |
|  |  |  |  |  |  |  |  |  |  |  |  |  |  |  |  | Medium | FSNDVVSGK | 1 | 2 | 1 | 658007487 |  | 0.00 | 2.05 | 2 | 952.47 | 0.02 | 37.43 | 0 |
|  |  |  |  |  |  |  |  |  |  |  |  |  |  |  |  | Low | NGLLEVQK | 1 | 3 | 1 | 658007487 | N1(Deamidated) | 0.00 | 1.63 | 2 | 901.50 | 0.39 | 41.81 | 0 |
|  |  |  |  |  |  |  |  |  |  |  |  |  |  |  |  | Low | AAGEVLALQK | 1 | 3 | 1 | 658007487 |  | 0.00 | 1.55 | 2 | 999.58 | -0.20 | 39.95 | 0 |
|  |  |  |  |  |  |  |  |  |  |  |  |  |  |  |  | Low | EFDLDGNR | 1 | 1 | 1 | 658007487 |  | 0.04 | 1.50 | 2 | 965.43 | -0.18 | 40.42 | 0 |
|  |  |  |  |  |  |  |  |  |  |  |  |  |  |  |  | Low | EAGLNFAK | 1 | 3 | 1 | 658007487 |  | 0.00 | 1.30 | 2 | 849.45 | 0.33 | 39.70 | 0 |
|  |  |  |  |  |  |  |  |  |  |  |  |  |  |  |  | Low | FQAAFK | 1 | 2 | 1 | 658007487 |  | 0.01 | 1.17 | 2 | 711.38 | 0.12 | 39.07 | 0 |
|  |  |  |  |  |  |  |  |  |  |  |  |  |  |  |  | Low | TLEALLK | 2 | 1 | 1 | 658007487 |  | 0.00 | 0.97 | 2 | 787.49 | -0.47 | 43.53 | 0 |
| 3522 | gi\|657959555 | Polyphenol oxidase |  | *Malus domestica* | Chloroplast | 20.1-Secondary metabolism/Phen ylpropanoids/Phe  nolics | 10.62 | 10.73 | 7 | 2 | 8 | 10 | 587 | 65.1 | 6.71 | High | LGYVYDEK | 2 | 4 | 1 | 657959555 |  | 0.00 | 2.77 | 2 | 986.48 | -0.37 | 39.28 | 0 |
|  |  |  |  |  |  |  |  |  |  |  |  |  |  |  |  | High | AQDEVLVIK | 1 | 5 | 1 | 657959555 |  | 0.00 | 2.56 | 2 | 1014.58 | 0.07 | 41.06 | 0 |
|  |  |  |  |  |  |  |  |  |  |  |  |  |  |  |  | Low | KAQDEVLVIK | 1 | 5 | 1 | 657959555 |  | 0.00 | 1.91 | 2 | 1142.68 | 0.33 | 40.22 | 1 |
|  |  |  |  |  |  |  |  |  |  |  |  |  |  |  |  | Low | LGGTDIEK | 1 | 1 | 1 | 657959555 |  | 0.00 | 1.63 | 2 | 832.44 | -0.04 | 36.85 | 0 |
|  |  |  |  |  |  |  |  |  |  |  |  |  |  |  |  | Low | ALPDDDPR | 2 | 6 | 1 | 657959555 |  | 0.00 | 1.25 | 2 | 898.43 | -0.11 | 36.69 | 0 |
|  |  |  |  |  |  |  |  |  |  |  |  |  |  |  |  | Low | AIELMR | 1 | 3 | 1 | 657959555 | M5(Oxidation) | 0.04 | 1.24 | 2 | 748.40 | 0.29 | 38.28 | 0 |
|  |  |  |  |  |  |  |  |  |  |  |  |  |  |  |  | Low | VKVCDSLDTK | 1 | 1 | 1 | 657959555 | C4(Carbamidomethyl) | 0.00 | 1.14 | 2 | 1164.59 | -1.48 | 37.85 | 1 |
|  |  |  |  |  |  |  |  |  |  |  |  |  |  |  |  | Low | ENLTTMYQQMVS  K | 1 | 3 | 1 | 657959555 | M6(Oxidation);  M10(Oxidation) | 0.00 | 1.14 | 2 | 1604.73 | 0.84 | 38.53 | 0 |
| 5414 | gi\|657979903 | Hydroxymethylglut aryl-CoA synthase |  | *Malus domestica* | Cytoplasm | 01.06-  Metabolism/Lipi d and sterol | 24.53 | 10.75 | 2 | 3 | 5 | 12 | 465 | 51.0 | 6.40 | High | DAAATTNANGVV ANGH | 2 | 1 | 1 | 657979903 | N9(Deamidated); N14(Deamidated) | 0.00 | 4.56 | 2 | 1484.66 | -0.55 | 36.70 | 0 |
|  |  |  |  |  |  |  |  |  |  |  |  |  |  |  |  | High | DAAATTNANGVV  ANGH | 2 | 1 | 1 | 657979903 | N9(Deamidated) | 0.00 | 4.18 | 2 | 1483.68 | -1.12 | 35.91 | 0 |
|  |  |  |  |  |  |  |  |  |  |  |  |  |  |  |  | High | DAAATTNANGVV  ANGH | 1 | 1 | 1 | 657979903 |  | 0.00 | 3.22 | 2 | 1482.69 | 0.86 | 35.25 | 0 |
|  |  |  |  |  |  |  |  |  |  |  |  |  |  |  |  | High | ASQQVAKPLYDA  K | 4 | 2 | 1 | 657979903 | Q3(Deamidated) | 0.00 | 2.96 | 3 | 1419.75 | 0.51 | 35.11 | 0 |
|  |  |  |  |  |  |  |  |  |  |  |  |  |  |  |  | High | LEVGSETVIDK | 1 | 2 | 1 | 657979903 |  | 0.00 | 2.70 | 2 | 1189.63 | -0.08 | 38.29 | 0 |
|  |  |  |  |  |  |  |  |  |  |  |  |  |  |  |  | Low | LEVGSETVIDKSK | 1 | 2 | 1 | 657979903 |  | 0.00 | 2.15 | 2 | 1404.76 | 0.16 | 36.58 | 1 |
|  |  |  |  |  |  |  |  |  |  |  |  |  |  |  |  | Low | LSQTCYLK | 1 | 2 | 1 | 657979903 | Q3(Deamidated);  C5(Carbamidomethyl) | 0.00 | 2.06 | 2 | 1013.50 | -0.11 | 36.80 | 0 |
| 5845 | gi\|657952945 | AP-2 complex subunit alpha |  | *Malus domestica* | Chloroplast | 07.99-  Transporters/Oth ers | 9.69 | 3.24 | 3 | 3 | 4 | 4 | 1019 | 113.5 | 6.11 | High | MAQLLDER | 1 | 2 | 1 | 657952945 | M1(Oxidation) | 0.00 | 2.69 | 2 | 991.49 | 0.38 | 36.91 | 0 |

|  |  |  |  |  |  |  |  |  |  |  |  |  |  |  |  | High | KAEDTEVDTAEQS  AIK | 1 | 3 | 1 | 657952945 |  | 0.00 | 2.43 | 3 | 1734.84 | 0.23 | 35.54 | 1 |
| --- | --- | --- | --- | --- | --- | --- | --- | --- | --- | --- | --- | --- | --- | --- | --- | --- | --- | --- | --- | --- | --- | --- | --- | --- | --- | --- | --- | --- | --- |
|  |  |  |  |  |  |  |  |  |  |  |  |  |  |  |  | High | AAEYLALSR | 1 | 3 | 1 | 657952945 |  | 0.00 | 2.41 | 2 | 993.54 | 0.38 | 39.06 | 0 |
|  |  |  |  |  |  |  |  |  |  |  |  |  |  |  |  | Low | AEDTEVDTAEQSA  IK | 1 | 3 | 1 | 657952945 |  | 0.00 | 2.16 | 2 | 1606.75 | 1.66 | 37.39 | 0 |
|  |  |  |  |  |  |  |  |  |  |  |  |  |  |  |  |  |  |  |  |  |  |  |  |  |  |  |  |  |  |
| 5845 | gi\|658027395 | Alpha-glucosidase |  | *Malus domestica* | Cytoplasm | 01.05-  Metabolism/Sug ars and polysaccharides | 6.95 | 3.78 | 1 | 2 | 3 | 3 | 793 | 88.1 | 6.00 | High | GANLNANSDIR | 1 | 1 | 1 | 658027395 |  | 0.00 | 2.56 | 2 | 1144.57 | 0.34 | 34.87 | 0 |
|  |  |  |  |  |  |  |  |  |  |  |  |  |  |  |  | High | DNYETYIR | 1 | 1 | 1 | 658027395 |  | 0.00 | 2.37 | 2 | 1073.49 | 0.08 | 39.39 | 0 |
|  |  |  |  |  |  |  |  |  |  |  |  |  |  |  |  | Low | SSIFGLGEHTK | 1 | 1 | 1 | 658027395 |  | 0.00 | 2.02 | 2 | 1175.61 | 0.69 | 38.13 | 0 |
| 5848 | gi\|658003341 | Glucan 1,3-alpha- glucosidase |  | *Malus domestica* | Vacuole | 09.01-Cell structure/Cell  wall | 18.61 | 6.75 | 2 | 5 | 6 | 7 | 934 | 106.7 | 6.37 | High | DALHVEGVEHR | 2 | 2 | 1 | 658003341 |  | 0.00 | 3.26 | 3 | 1261.63 | -0.15 | 34.69 | 0 |
|  |  |  |  |  |  |  |  |  |  |  |  |  |  |  |  | High | GPGVEVSEPYR | 1 | 2 | 1 | 658003341 |  | 0.00 | 3.01 | 2 | 1189.59 | 0.66 | 36.82 | 0 |
|  |  |  |  |  |  |  |  |  |  |  |  |  |  |  |  | High | DEEDVEQVDSK | 1 | 2 | 1 | 658003341 |  | 0.00 | 2.58 | 2 | 1292.55 | 1.20 | 35.89 | 0 |
|  |  |  |  |  |  |  |  |  |  |  |  |  |  |  |  | High | GALIEPANQK | 1 | 2 | 1 | 658003341 |  | 0.00 | 2.40 | 2 | 1040.57 | 0.58 | 35.47 | 0 |
|  |  |  |  |  |  |  |  |  |  |  |  |  |  |  |  | High | HASVYLPGK | 1 | 2 | 1 | 658003341 |  | 0.00 | 2.32 | 2 | 971.53 | -0.24 | 34.69 | 0 |
|  |  |  |  |  |  |  |  |  |  |  |  |  |  |  |  | Low | MLFPHPEEMQR | 1 | 2 | 1 | 658003341 | M1(Oxidation);  M9(Oxidation) | 0.00 | 2.01 | 2 | 1446.65 | 0.20 | 36.37 | 0 |
| 5848 | gi\|658001510 | Chaperone protein |  | *Malus domestica* | Chloroplast | 06.01-Protein destination and storage/Folding  and Stabillity | 16.70 | 5.48 | 7 | 5 | 5 | 6 | 912 | 100.9 | 6.15 | High | TAVVEGLAQR | 1 | 3 | 1 | 658001510 | Q9(Deamidated) | 0.00 | 3.20 | 2 | 1044.57 | -0.40 | 37.61 | 0 |
| 5848 |  |  |  |  |  |  |  |  |  |  |  |  |  |  |  | High | TAVVEGLAQR | 1 | 3 | 1 | 658001510 |  | 0.00 | 3.17 | 2 | 1043.58 | -0.04 | 36.85 | 0 |
| 5848 |  |  |  |  |  |  |  |  |  |  |  |  |  |  |  | High | VESASGDTTFQAL  K | 1 | 3 | 1 | 658001510 |  | 0.00 | 2.93 | 2 | 1453.72 | 1.00 | 38.46 | 0 |
| 5848 |  |  |  |  |  |  |  |  |  |  |  |  |  |  |  | High | TKNNPVLIGEPGV  GK | 1 | 7 | 1 | 658001510 |  | 0.00 | 2.69 | 3 | 1522.86 | 0.25 | 36.60 | 1 |
| 5848 |  |  |  |  |  |  |  |  |  |  |  |  |  |  |  | High | NNPVLIGEPGVGK | 1 | 7 | 1 | 658001510 |  | 0.00 | 2.56 | 2 | 1293.72 | 0.78 | 38.91 | 0 |
| 5848 |  |  |  |  |  |  |  |  |  |  |  |  |  |  |  | Medium | ALVVAAQLSSR | 1 | 2 | 1 | 658001510 |  | 0.00 | 2.16 | 2 | 1114.66 | -1.04 | 38.87 | 0 |
| 6019 | gi\|657976562 | Oxygen-evolving enhancer protein |  | *Malus domestica* | Chloroplast | 11.02-  Disease/defense/ Defense-related | 7.46 | 15.67 | 2 | 2 | 3 | 3 | 268 | 28.7 | 8.59 | High | TADGDEGGKHQLI TATVK | 1 | 2 | 1 | 657976562 |  | 0.00 | 3.01 | 3 | 1840.94 | 0.37 | 34.67 | 1 |
| 6019 |  |  |  |  |  |  |  |  |  |  |  |  |  |  |  | High | EVEFPGQVLR | 1 | 1 | 1 | 657976562 |  | 0.00 | 2.44 | 2 | 1173.63 | 0.17 | 42.02 | 0 |
| 6019 |  |  |  |  |  |  |  |  |  |  |  |  |  |  |  | Low | SIADYGSPEEFLAK | 1 | 1 | 1 | 657976562 |  | 0.00 | 2.01 | 2 | 1526.74 | 1.82 | 42.99 | 0 |
| 6019 | gi\|657955010 | Glutathione S- transferase |  | *Malus domestica* | Cytosol | 11.06-  Disease/Defense  /Detoxification | 5.40 | 6.57 | 3 | 1 | 1 | 2 | 213 | 24.0 | 6.65 | High | MTTDAAVVEENE AK | 1 | 3 | 1 | 657955010 | M1(Oxidation) | 0.00 | 2.81 | 2 | 1523.69 | 1.38 | 35.25 | 0 |
| 6019 |  |  |  |  |  |  |  |  |  |  |  |  |  |  |  | High | MTTDAAVVEENE  AK | 1 | 3 | 1 | 657955010 |  | 0.00 | 2.60 | 2 | 1507.70 | 0.94 | 36.54 | 0 |
| 6222 | gi\|657993900 | Fructose- bisphosphate  aldolase |  | *Malus domestica* | Chloroplast | 02.01-  Energy/Glycolys is | 19.66 | 11.45 | 3 | 4 | 4 | 6 | 393 | 42.5 | 8.02 | High | GILAIDESNATCG K | 1 | 2 | 1 | 657993900 | C12(Carbamidomethyl) | 0.00 | 4.34 | 2 | 1448.71 | 1.24 | 39.82 | 0 |
|  |  |  |  |  |  |  |  |  |  |  |  |  |  |  |  | High | GILAIDESNATCG K | 1 | 2 | 1 | 657993900 | N9(Deamidated); C12(Carbamidomethyl) | 0.00 | 4.06 | 2 | 1449.69 | -0.71 | 40.74 | 0 |
|  |  |  |  |  |  |  |  |  |  |  |  |  |  |  |  | High | LDSIGLDNTEVNR | 1 | 2 | 1 | 657993900 |  | 0.00 | 3.26 | 2 | 1445.73 | 1.31 | 39.61 | 0 |
|  |  |  |  |  |  |  |  |  |  |  |  |  |  |  |  | High | FVDVLCDQK | 1 | 2 | 1 | 657993900 | C6(Carbamidomethyl) | 0.00 | 2.78 | 2 | 1123.55 | -0.05 | 40.00 | 0 |
|  |  |  |  |  |  |  |  |  |  |  |  |  |  |  |  | High | FVDVLCDQK | 1 | 2 | 1 | 657993900 | C6(Carbamidomethyl);  Q8(Deamidated) | 0.00 | 2.72 | 2 | 1124.53 | 0.70 | 41.11 | 0 |
|  |  |  |  |  |  |  |  |  |  |  |  |  |  |  |  | High | ANSLAQLGK | 1 | 3 | 1 | 657993900 |  | 0.00 | 2.50 | 2 | 901.51 | -0.57 | 36.10 | 0 |
| 6222 | gi\|658009454 | Uncharacterized  protein |  |  | Cell membrane | 12-Unclear  classification | 12.93 | 10.36 | 1 | 3 | 4 | 6 | 386 | 42.7 | 7.72 | High | DGFEVGNADK | 1 | 1 | 1 | 658009454 |  | 0.00 | 3.24 | 2 | 1051.47 | 0.14 | 38.01 | 0 |
|  |  |  |  |  |  |  |  |  |  |  |  |  |  |  |  | High | TFGVNGYGIDSMA  K | 1 | 1 | 1 | 658009454 | M12(Oxidation) | 0.00 | 2.66 | 2 | 1475.69 | 1.28 | 39.83 | 0 |
|  |  |  |  |  |  |  |  |  |  |  |  |  |  |  |  | High | SLNQFIEK | 2 | 1 | 1 | 658009454 | Q4(Deamidated) | 0.00 | 2.64 | 2 | 979.51 | -0.14 | 39.45 | 0 |
|  |  |  |  |  |  |  |  |  |  |  |  |  |  |  |  | Low | SLNQFIEK | 1 | 1 | 1 | 658009454 |  | 0.00 | 2.27 | 2 | 978.53 | -0.51 | 38.67 | 0 |
|  |  |  |  |  |  |  |  |  |  |  |  |  |  |  |  | Low | EELRFPAK | 1 | 1 | 1 | 658009454 |  | 0.00 | 2.13 | 2 | 989.54 | -0.58 | 36.36 | 1 |
| 6222 | gi\|658012400 | Formate dehydrogenase |  | *Malus domestica* | Mitochondrion | 11.06-  Disease/Defense  /Detoxification | 7.45 | 6.49 | 1 | 2 | 3 | 3 | 385 | 42.1 | 6.99 | High | IDPELEQQTGAK | 1 | 1 | 1 | 658012400 |  | 0.00 | 2.63 | 2 | 1328.67 | 0.69 | 36.42 | 0 |
|  |  |  |  |  |  |  |  |  |  |  |  |  |  |  |  | High | VKIDPELEQQTGA  K | 1 | 1 | 1 | 658012400 |  | 0.00 | 2.51 | 2 | 1555.83 | 1.41 | 36.34 | 1 |
|  |  |  |  |  |  |  |  |  |  |  |  |  |  |  |  | Low | FEEDLDAMLPK | 1 | 1 | 1 | 658012400 | M8(Oxidation) | 0.00 | 2.31 | 2 | 1323.61 | 0.68 | 40.58 | 0 |
| 7220 | gi\|658062690 | Voltage-gated potassium channel  subunit beta |  | *Malus domestica* | Chloroplast | 07.01-  Transporterts/Ion s | 8.77 | 7.76 | 3 | 1 | 2 | 3 | 232 | 26.3 | 5.62 | High | AEEIMGQAIR | 1 | 3 | 1 | 658062690 | M5(Oxidation) | 0.00 | 3.47 | 2 | 1133.56 | 0.05 | 35.35 | 0 |
|  |  |  |  |  |  |  |  |  |  |  |  |  |  |  |  | High | AEEIMGQAIR | 1 | 3 | 1 | 658062690 | M5(Oxidation);  Q7(Deamidated) | 0.00 | 3.12 | 2 | 1134.55 | 1.44 | 36.14 | 0 |

|  |  |  |  |  |  |  |  |  |  |  |  |  |  |  |  | Low | AMNYVIDK | 1 | 3 | 1 | 658062690 | M2(Oxidation) | 0.00 | 2.19 | 2 | 969.47 | 0.59 | 35.60 | 0 |
| --- | --- | --- | --- | --- | --- | --- | --- | --- | --- | --- | --- | --- | --- | --- | --- | --- | --- | --- | --- | --- | --- | --- | --- | --- | --- | --- | --- | --- | --- |
| 7222 | gi\|657989479 | Fructose- bisphosphate aldolase cytoplasmic isozyme |  | *Malus domestica* | Cytosol | 02.01-  Energy/Glycolys is | 10.01 | 7.26 | 9 | 2 | 2 | 3 | 358 | 38.4 | 8.31 | High | GILAADESTGTIGK | 1 | 9 | 1 | 657989479 |  | 0.00 | 4.07 | 2 | 1332.70 | 0.91 | 39.12 | 0 |
|  |  |  |  |  |  |  |  |  |  |  |  |  |  |  |  | High | LGEGAAESLHVK | 2 | 3 | 1 | 657989479 |  | 0.00 | 3.18 | 2 | 1210.64 | 0.35 | 36.15 | 0 |
| 7222 | gi\|657980729 | NADP-dependent D-sorbitol-6- phosphate  dehydrogenase |  | *Malus domestica* | Cytoplasm | 02.10-  Energy/TCA pathway | 9.95 | 8.39 | 2 | 3 | 3 | 4 | 310 | 34.9 | 7.23 | High | REELFITTK | 1 | 2 | 1 | 657980729 |  | 0.00 | 2.89 | 2 | 1136.63 | -0.23 | 37.39 | 1 |
|  |  |  |  |  |  |  |  |  |  |  |  |  |  |  |  | High | TASLLGEDK | 2 | 2 | 1 | 657980729 |  | 0.00 | 2.44 | 2 | 933.49 | -0.44 | 37.10 | 0 |
|  |  |  |  |  |  |  |  |  |  |  |  |  |  |  |  | High | SVAQICLR | 1 | 2 | 1 | 657980729 | C6(Carbamidomethyl) | 0.00 | 2.43 | 2 | 946.51 | -0.13 | 37.66 | 0 |
| 7226 | gi\|658057933 | NADP-dependent D-sorbitol-6- phosphate  dehydrogenase |  | *Malus domestica* | Cytoplasm | 02.10-  Energy/TCA pathway | 5.93 | 5.82 | 5 | 2 | 2 | 2 | 292 | 32.9 | 6.29 | High | REDLFITTK | 1 | 5 | 1 | 658057933 |  | 0.00 | 2.97 | 2 | 1122.62 | 0.33 | 37.98 | 1 |
|  |  |  |  |  |  |  |  |  |  |  |  |  |  |  |  | High | TVAQVVLR | 1 | 2 | 1 | 658057933 |  | 0.00 | 2.95 | 2 | 885.55 | -0.26 | 37.73 | 0 |
| 8123 | gi\|657948234 | Proteasome subunit alpha |  | *Malus domestica* | Cytosol | 06.13-Protein destination and storage/Proteolys  is | 22.53 | 16.06 | 4 | 3 | 5 | 9 | 249 | 27.1 | 7.43 | High | ALLEVVESGGK | 2 | 4 | 1 | 657948234 |  | 0.00 | 3.53 | 2 | 1101.62 | 0.29 | 41.72 | 0 |
|  |  |  |  |  |  |  |  |  |  |  |  |  |  |  |  | High | GTDTIVLGVEKK | 1 | 2 | 1 | 657948234 |  | 0.00 | 2.90 | 2 | 1259.72 | 0.79 | 37.03 | 1 |
|  |  |  |  |  |  |  |  |  |  |  |  |  |  |  |  | High | GTDTIVLGVEK | 2 | 2 | 1 | 657948234 |  | 0.00 | 2.75 | 2 | 1131.63 | 0.23 | 40.54 | 0 |
|  |  |  |  |  |  |  |  |  |  |  |  |  |  |  |  | Low | NIEVAVMTK | 1 | 2 | 1 | 657948234 | M7(Oxidation) | 0.00 | 2.59 | 2 | 1020.54 | -0.77 | 36.35 | 0 |
|  |  |  |  |  |  |  |  |  |  |  |  |  |  |  |  | Low | YIAGLQQK | 2 | 2 | 1 | 657948234 | Q7(Deamidated) | 0.00 | 2.40 | 2 | 921.50 | 0.57 | 36.83 | 0 |
|  |  |  |  |  |  |  |  |  |  |  |  |  |  |  |  | Low | NIEVAVMTK | 1 | 2 | 1 | 657948234 |  | 0.00 | 2.15 | 2 | 1004.54 | 0.05 | 38.62 | 0 |
| 8327 | gi\|657977276 | Elongation factor |  | *Malus domestica* | Ribosome | 05.04-Protein synthesis/Transla  tion factors | 16.50 | 13.87 | 6 | 1 | 6 | 7 | 447 | 49.3 | 9.07 | High | YYCTVIDAPGHR | 2 | 6 | 1 | 657977276 | C3(Carbamidomethyl) | 0.00 | 2.94 | 3 | 1451.67 | -0.17 | 36.70 | 0 |
|  |  |  |  |  |  |  |  |  |  |  |  |  |  |  |  | Low | GFVASNSKDDPAK | 1 | 3 | 1 | 657977276 |  | 0.00 | 2.45 | 2 | 1335.65 | 0.63 | 33.11 | 1 |
|  |  |  |  |  |  |  |  |  |  |  |  |  |  |  |  | Low | ARYEEIVK | 1 | 2 | 1 | 657977276 |  | 0.00 | 2.33 | 3 | 1007.55 | 0.49 | 34.50 | 1 |
|  |  |  |  |  |  |  |  |  |  |  |  |  |  |  |  | Low | IGGIGTVPVGR | 1 | 6 | 1 | 657977276 |  | 0.00 | 2.32 | 2 | 1025.61 | -0.23 | 38.82 | 0 |
|  |  |  |  |  |  |  |  |  |  |  |  |  |  |  |  | Low | STTTGHLIYK | 1 | 6 | 1 | 657977276 |  | 0.00 | 2.28 | 2 | 1120.60 | 0.05 | 34.11 | 0 |
|  |  |  |  |  |  |  |  |  |  |  |  |  |  |  |  | Low | LPLQDVYK | 1 | 6 | 1 | 657977276 | Q4(Deamidated) | 0.00 | 2.04 | 2 | 976.54 | 0.26 | 40.17 | 0 |
| 8327 | gi\|657949619 | 26S protease regulatory subunit 8 homolog A |  | *Malus domestica* | Cytoplasm/Nuc leus | 06.13-Protein destination and storage/Proteolys  is | 15.71 | 11.99 | 2 | 2 | 5 | 6 | 417 | 47.0 | 8.60 | High | AVCTEAGMFALR | 1 | 2 | 1 | 657949619 | C3(Carbamidomethyl); M8(Oxidation) | 0.00 | 3.00 | 2 | 1341.63 | 0.84 | 39.21 | 0 |
|  |  |  |  |  |  |  |  |  |  |  |  |  |  |  |  | High | VHVTQEDFEMAV  AK | 1 | 2 | 1 | 657949619 | M10(Oxidation) | 0.00 | 2.93 | 2 | 1619.77 | 0.54 | 37.68 | 0 |
|  |  |  |  |  |  |  |  |  |  |  |  |  |  |  |  | Low | KIEFPNPSEESR | 1 | 2 | 1 | 657949619 |  | 0.00 | 2.79 | 2 | 1432.71 | 1.00 | 37.34 | 1 |
|  |  |  |  |  |  |  |  |  |  |  |  |  |  |  |  | Low | GVLLYGPPGTGK | 2 | 4 | 2 | 657949619;65  7980610 |  | 0.00 | 2.64 | 2 | 1158.65 | 0.41 | 40.01 | 0 |
|  |  |  |  |  |  |  |  |  |  |  |  |  |  |  |  | Low | IEFPNPSEESR | 1 | 2 | 1 | 657949619 |  | 0.00 | 2.11 | 2 | 1304.61 | 0.62 | 39.54 | 0 |
| 8327 | gi\|658029902 | 60S ribosomal protein L4 |  | *Malus domestica* | Cytosol | 05.01-Protein  synthesis/Riboso mal proteins | 8.60 | 5.43 | 3 | 2 | 3 | 3 | 405 | 44.8 | 10.32 | High | MSLLAEEQR | 1 | 2 | 1 | 658029902 | M1(Oxidation) | 0.00 | 3.21 | 2 | 1092.54 | 0.11 | 36.66 | 0 |
|  |  |  |  |  |  |  |  |  |  |  |  |  |  |  |  | High | GPLIVYGTEGAK | 1 | 3 | 1 | 658029902 |  | 0.00 | 3.15 | 2 | 1204.66 | 0.21 | 39.17 | 0 |
|  |  |  |  |  |  |  |  |  |  |  |  |  |  |  |  | Low | KGPLIVYGTEGAK | 1 | 3 | 1 | 658029902 |  | 0.00 | 2.24 | 2 | 1332.75 | 1.01 | 36.78 | 1 |
| 8726 | gi\|657979411 | Dynamin |  | *Malus domestica* | Cytoplasm | 03.22-Cell growth/division/  Cell Cycle | 40.24 | 16.74 | 2 | 4 | 13 | 17 | 926 | 100.3 | 9.06 | High | SAFDAAANGPVD R | 1 | 2 | 1 | 657979411 |  | 0.00 | 4.05 | 2 | 1290.61 | 0.26 | 37.65 | 0 |
|  |  |  |  |  |  |  |  |  |  |  |  |  |  |  |  | High | IEELLQEDQNVK | 1 | 2 | 1 | 657979411 |  | 0.00 | 3.51 | 2 | 1457.75 | 1.46 | 39.15 | 0 |
|  |  |  |  |  |  |  |  |  |  |  |  |  |  |  |  | High | SAFDAAANGPVD  R | 1 | 2 | 1 | 657979411 | N8(Deamidated) | 0.00 | 3.27 | 2 | 1291.59 | -0.13 | 38.44 | 0 |
|  |  |  |  |  |  |  |  |  |  |  |  |  |  |  |  | High | HYSDPAQNGDVN  SGPNSGSR | 3 | 2 | 1 | 657979411 | N12(Deamidated) | 0.00 | 3.04 | 3 | 2059.87 | -1.09 | 34.07 | 0 |
|  |  |  |  |  |  |  |  |  |  |  |  |  |  |  |  | High | MVVALVDMER | 1 | 2 | 1 | 657979411 | M1(Oxidation);  M8(Oxidation) | 0.00 | 2.98 | 2 | 1194.59 | 1.08 | 38.24 | 0 |
|  |  |  |  |  |  |  |  |  |  |  |  |  |  |  |  | Low | RPADPEEELR | 1 | 2 | 1 | 657979411 |  | 0.00 | 2.69 | 3 | 1211.60 | -0.02 | 34.64 | 0 |
|  |  |  |  |  |  |  |  |  |  |  |  |  |  |  |  | Low | HYSDPAQNGDVN  SGPNSGSR | 1 | 2 | 1 | 657979411 |  | 0.00 | 2.60 | 3 | 2058.89 | 1.04 | 33.50 | 0 |
|  |  |  |  |  |  |  |  |  |  |  |  |  |  |  |  | Low | SQIVQDELVK | 1 | 2 | 1 | 657979411 |  | 0.00 | 2.49 | 2 | 1158.64 | -0.11 | 37.91 | 0 |
|  |  |  |  |  |  |  |  |  |  |  |  |  |  |  |  | Low | SQQVSASALR | 1 | 2 | 1 | 657979411 |  | 0.00 | 2.42 | 2 | 1046.56 | -0.05 | 34.48 | 0 |
|  |  |  |  |  |  |  |  |  |  |  |  |  |  |  |  | Low | AAAASSYSGGGG  AQESISR | 1 | 1 | 1 | 657979411 |  | 0.00 | 2.28 | 2 | 1726.80 | 1.46 | 34.87 | 0 |
|  |  |  |  |  |  |  |  |  |  |  |  |  |  |  |  | Low | SLALELCR | 1 | 2 | 1 | 657979411 | C7(Carbamidomethyl) | 0.00 | 2.25 | 2 | 961.51 | -0.36 | 40.17 | 0 |
|  |  |  |  |  |  |  |  |  |  |  |  |  |  |  |  | Low | LIDLPGLDQR | 1 | 2 | 1 | 657979411 |  | 0.00 | 2.24 | 2 | 1139.64 | 0.58 | 43.34 | 0 |
|  |  |  |  |  |  |  |  |  |  |  |  |  |  |  |  | Low | APISIDLQR | 1 | 2 | 1 | 657979411 |  | 0.00 | 2.24 | 2 | 1012.58 | 0.04 | 39.69 | 0 |
|  |  |  |  |  |  |  |  |  |  |  |  |  |  |  |  | Low | GQDAEQAIMNR | 1 | 1 | 1 | 657979411 | M9(Oxidation) | 0.00 | 2.16 | 2 | 1248.56 | 0.17 | 34.75 | 0 |
|  |  |  |  |  |  |  |  |  |  |  |  |  |  |  |  | Low | QSLSDGSLDTMTR | 1 | 2 | 1 | 657979411 | M11(Oxidation) | 0.00 | 2.02 | 2 | 1426.65 | 1.17 | 36.57 | 0 |

| 8726 | gi\|657991756 | Acetyl-coenzyme A carboxylase carboxyl transferase subunit  alpha |  | *Malus domestica* | Mitochondrion | 01.06-  Metabolism/Lipi d and sterol | 15.08 | 9.37 | 1 | 1 | 6 | 6 | 758 | 84.3 | 8.87 | High | IEDLNGEINER | 1 | 1 | 1 | 657991756 |  | 0.00 | 2.97 | 2 | 1301.64 | 1.58 | 37.88 | 0 |
| --- | --- | --- | --- | --- | --- | --- | --- | --- | --- | --- | --- | --- | --- | --- | --- | --- | --- | --- | --- | --- | --- | --- | --- | --- | --- | --- | --- | --- | --- |
|  |  |  |  |  |  |  |  |  |  |  |  |  |  |  |  | Low | QSLAAAVESSNLK | 1 | 1 | 1 | 657991756 |  | 0.00 | 2.79 | 2 | 1317.70 | 0.50 | 38.79 | 0 |
|  |  |  |  |  |  |  |  |  |  |  |  |  |  |  |  | Low | TPVADLEGEVEK | 1 | 1 | 1 | 657991756 |  | 0.00 | 2.70 | 2 | 1286.65 | 1.33 | 39.62 | 0 |
|  |  |  |  |  |  |  |  |  |  |  |  |  |  |  |  | Low | KPVEQTLPSEIK | 1 | 1 | 1 | 657991756 |  | 0.00 | 2.28 | 2 | 1368.77 | 1.06 | 35.83 | 0 |
|  |  |  |  |  |  |  |  |  |  |  |  |  |  |  |  | Low | DDEDPYSFDASR | 1 | 1 | 1 | 657991756 |  | 0.00 | 2.27 | 2 | 1416.56 | 1.50 | 41.42 | 0 |
|  |  |  |  |  |  |  |  |  |  |  |  |  |  |  |  | Low | NFGMPTPHGYR | 1 | 1 | 1 | 657991756 | M4(Oxidation) | 0.00 | 2.07 | 2 | 1292.58 | 0.50 | 34.55 | 0 |
| 8728 | gi\|657988901 | Cullin |  | *Malus domestica* | Nucleus | 03.22-Cell growth/division/  Cell Cycle | 13.80 | 7.86 | 1 | 2 | 6 | 7 | 738 | 85.9 | 7.06 | High | LDKVDDLSR | 1 | 1 | 1 | 657988901 |  | 0.00 | 2.72 | 3 | 1060.56 | 0.48 | 35.17 | 1 |
|  |  |  |  |  |  |  |  |  |  |  |  |  |  |  |  | High | GGAGTQEQVLIK | 2 | 1 | 1 | 657988901 | Q6(Deamidated) | 0.00 | 2.49 | 2 | 1201.64 | 0.32 | 37.29 | 0 |
|  |  |  |  |  |  |  |  |  |  |  |  |  |  |  |  | Low | IAVIDLINK | 1 | 1 | 1 | 657988901 |  | 0.00 | 2.26 | 2 | 998.62 | -0.38 | 43.30 | 0 |
|  |  |  |  |  |  |  |  |  |  |  |  |  |  |  |  | Low | MEGMVTDLTIAR | 1 | 1 | 1 | 657988901 | M1(Oxidation);  M4(Oxidation) | 0.00 | 2.23 | 2 | 1368.65 | 1.14 | 38.89 | 0 |
|  |  |  |  |  |  |  |  |  |  |  |  |  |  |  |  | Low | YAIDAAIVR | 1 | 1 | 1 | 657988901 |  | 0.00 | 2.05 | 2 | 991.56 | 0.15 | 40.12 | 0 |
|  |  |  |  |  |  |  |  |  |  |  |  |  |  |  |  | Low | IEDLITR | 1 | 1 | 1 | 657988901 |  | 0.00 | 2.04 | 2 | 859.49 | -0.30 | 38.42 | 0 |
| 8732 | gi\|658038306 | Porin |  | *Malus domestica* | Mitochondrion | 11.02-  Disease/defense/ Defense-related | 27.18 | 23.91 | 2 | 3 | 7 | 10 | 276 | 29.5 | 8.79 | High | GPGLFTDIGK | 2 | 2 | 1 | 658038306 |  | 0.00 | 3.45 | 2 | 1004.54 | -0.30 | 42.57 | 0 |
|  |  |  |  |  |  |  |  |  |  |  |  |  |  |  |  | High | SVLTISGEVDTK | 1 | 2 | 1 | 658038306 |  | 0.00 | 2.99 | 2 | 1248.67 | 0.51 | 39.78 | 0 |
|  |  |  |  |  |  |  |  |  |  |  |  |  |  |  |  | High | GGLSTGDILTQYK | 1 | 1 | 1 | 658038306 |  | 0.00 | 2.97 | 2 | 1352.71 | 0.95 | 41.10 | 0 |
|  |  |  |  |  |  |  |  |  |  |  |  |  |  |  |  | Low | YKNTVVDVK | 1 | 2 | 1 | 658038306 |  | 0.00 | 2.78 | 2 | 1065.59 | -0.12 | 34.13 | 1 |
|  |  |  |  |  |  |  |  |  |  |  |  |  |  |  |  | Low | LGALLQHEVIPK | 1 | 1 | 1 | 658038306 |  | 0.00 | 2.71 | 3 | 1317.79 | -0.13 | 38.91 | 0 |
|  |  |  |  |  |  |  |  |  |  |  |  |  |  |  |  | Low | SAAVGEITR | 3 | 1 | 1 | 658038306 |  | 0.00 | 2.56 | 2 | 903.49 | -0.32 | 35.86 | 0 |
|  |  |  |  |  |  |  |  |  |  |  |  |  |  |  |  | Low | GPGLFTDIGKK | 1 | 2 | 1 | 658038306 |  | 0.00 | 2.18 | 2 | 1132.64 | 0.44 | 38.59 | 1 |
| 9603 | gi\|657996330 | ATP-citrate synthase beta chain  protein |  | *Malus domestica* | Mitochondrion | 07.22-  Transporters/Tra nsport ATPases | 34.64 | 16.94 | 2 | 9 | 9 | 15 | 608 | 65.9 | 7.65 | High | TTQALFYNYK | 1 | 2 | 1 | 657996330 |  | 0.00 | 3.08 | 2 | 1248.63 | 0.66 | 39.73 | 0 |
|  |  |  |  |  |  |  |  |  |  |  |  |  |  |  |  | High | TTQALFYNYK | 2 | 2 | 1 | 657996330 | N8(Deamidated) | 0.00 | 3.02 | 2 | 1249.61 | 1.63 | 40.63 | 0 |
|  |  |  |  |  |  |  |  |  |  |  |  |  |  |  |  | High | VELLQAFAR | 1 | 2 | 1 | 657996330 |  | 0.00 | 2.98 | 2 | 1046.60 | -0.42 | 43.17 | 0 |
|  |  |  |  |  |  |  |  |  |  |  |  |  |  |  |  | High | SGGMSNELYNTV  AR | 2 | 2 | 1 | 657996330 | M4(Oxidation);  N10(Deamidated) | 0.00 | 2.90 | 2 | 1515.67 | -1.19 | 38.57 | 0 |
|  |  |  |  |  |  |  |  |  |  |  |  |  |  |  |  | High | DEYSLVEALK | 1 | 2 | 1 | 657996330 |  | 0.00 | 2.76 | 2 | 1166.59 | 0.57 | 44.38 | 0 |
|  |  |  |  |  |  |  |  |  |  |  |  |  |  |  |  | High | SGGMSNELYNTV  AR | 1 | 2 | 1 | 657996330 | M4(Oxidation) | 0.00 | 2.73 | 2 | 1514.69 | 0.75 | 37.48 | 0 |
|  |  |  |  |  |  |  |  |  |  |  |  |  |  |  |  | High | LYRPGSVGFVSK | 1 | 2 | 1 | 657996330 |  | 0.00 | 2.68 | 3 | 1309.72 | -3.19 | 37.07 | 0 |
|  |  |  |  |  |  |  |  |  |  |  |  |  |  |  |  | High | FGGAVDDAAR | 2 | 2 | 1 | 657996330 |  | 0.00 | 2.62 | 2 | 978.46 | -0.09 | 35.70 | 0 |
|  |  |  |  |  |  |  |  |  |  |  |  |  |  |  |  | High | SIGLIGHTFDQK | 1 | 2 | 1 | 657996330 |  | 0.00 | 2.45 | 2 | 1315.70 | 0.80 | 39.30 | 0 |
|  |  |  |  |  |  |  |  |  |  |  |  |  |  |  |  | High | VVAIIAEGVPESDT  K | 1 | 2 | 1 | 657996330 |  | 0.00 | 2.45 | 2 | 1527.83 | 1.30 | 40.68 | 0 |
|  |  |  |  |  |  |  |  |  |  |  |  |  |  |  |  | High | LIVVLGELGGR | 1 | 2 | 1 | 657996330 |  | 0.00 | 2.37 | 2 | 1125.70 | -0.39 | 44.01 | 0 |
|  |  |  |  |  |  |  |  |  |  |  |  |  |  |  |  | Low | VELLQAFAR | 1 | 2 | 1 | 657996330 | Q5(Deamidated) | 0.00 | 2.16 | 2 | 1047.58 | 0.62 | 44.22 | 0 |
| 9708 | gi\|657947113 | Beta-galactosidase |  | *Malus domestica* | Secreted | 01.05-  Metabolism/Sug ars and polysaccharides | 23.86 | 11.86 | 7 | 5 | 7 | 12 | 725 | 81.1 | 8.03 | High | SGCAAFLANYDT K | 1 | 1 | 1 | 657947113 | C3(Carbamidomethyl) | 0.00 | 3.47 | 2 | 1417.64 | 0.74 | 39.91 | 0 |
|  |  |  |  |  |  |  |  |  |  |  |  |  |  |  |  | High | LGNNQEAHVFNT  K | 4 | 1 | 1 | 657947113 | N11(Deamidated) | 0.00 | 2.73 | 2 | 1472.71 | -0.79 | 36.33 | 0 |
|  |  |  |  |  |  |  |  |  |  |  |  |  |  |  |  | High | ATFNAPPGHAPLA  LDMGSMGK | 1 | 1 | 1 | 657947113 | M16(Oxidation);  M19(Oxidation) | 0.00 | 2.71 | 3 | 2115.00 | 0.47 | 38.61 | 0 |
|  |  |  |  |  |  |  |  |  |  |  |  |  |  |  |  | High | LGNNQEAHVFNT  K | 1 | 1 | 1 | 657947113 |  | 0.00 | 2.69 | 2 | 1471.73 | 0.95 | 35.23 | 0 |
|  |  |  |  |  |  |  |  |  |  |  |  |  |  |  |  | High | ILISGSIHYPR | 1 | 7 | 1 | 657947113 |  | 0.00 | 2.64 | 2 | 1255.72 | 0.76 | 38.74 | 0 |
|  |  |  |  |  |  |  |  |  |  |  |  |  |  |  |  | High | LVQQAGLFVNLR | 1 | 1 | 1 | 657947113 |  | 0.00 | 2.63 | 2 | 1357.80 | 1.06 | 42.78 | 0 |
|  |  |  |  |  |  |  |  |  |  |  |  |  |  |  |  | Low | LTFSQNVK | 2 | 1 | 1 | 657947113 | N6(Deamidated) | 0.00 | 2.27 | 2 | 937.50 | 1.42 | 37.79 | 0 |
|  |  |  |  |  |  |  |  |  |  |  |  |  |  |  |  | Low | AIIINGQR | 1 | 3 | 1 | 657947113 |  | 0.00 | 2.15 | 2 | 884.53 | -0.04 | 36.29 | 0 |
| 9708 | gi\|657955906 | Peroxisomal acyl- coenzyme A  oxidase 1 |  | *Malus domestica* | Proxisome | 01.06-  Metabolism/Lipi d and sterol | 9.56 | 8.13 | 1 | 2 | 4 | 4 | 664 | 74.2 | 7.87 | High | FGSGAYNSMDNGVL R | 1 | 1 | 1 | 657955906 | M9(Oxidation) | 0.00 | 2.68 | 2 | 1603.72 | 1.42 | 38.36 | 0 |
|  |  |  |  |  |  |  |  |  |  |  |  |  |  |  |  | High | QTIVADASSALSR | 1 | 1 | 1 | 657955906 |  | 0.00 | 2.44 | 2 | 1318.70 | 1.32 | 39.81 | 0 |
|  |  |  |  |  |  |  |  |  |  |  |  |  |  |  |  | Low | TDEFVINSPTLTSS  K | 1 | 1 | 1 | 657955906 |  | 0.00 | 2.28 | 2 | 1638.83 | 3.66 | 41.66 | 0 |
|  |  |  |  |  |  |  |  |  |  |  |  |  |  |  |  | Low | MSIACAQDLSK | 1 | 1 | 1 | 657955906 | M1(Oxidation);  C5(Carbamidomethyl) | 0.00 | 2.17 | 2 | 1239.57 | 0.39 | 36.26 | 0 |
| 9708 | gi\|657968408 | Methyltransferase |  | *Malus domestica* | Chloroplast | 12-Unclear  classification | 6.85 | 5.06 | 1 | 1 | 3 | 3 | 632 | 71.8 | 7.93 | High | SIMDAMQYDVR | 1 | 1 | 1 | 657968408 | M3(Oxidation);  M6(Oxidation) | 0.00 | 2.49 | 2 | 1360.59 | 0.97 | 37.19 | 0 |
|  |  |  |  |  |  |  |  |  |  |  |  |  |  |  |  | Low | LINLGDGSVR | 1 | 1 | 1 | 657968408 |  | 0.00 | 2.24 | 2 | 1043.58 | -0.28 | 39.44 | 0 |
|  |  |  |  |  |  |  |  |  |  |  |  |  |  |  |  | Low | TMDYQLAEPGR | 1 | 1 | 1 | 657968408 | M2(Oxidation) | 0.00 | 2.13 | 2 | 1296.59 | 1.21 | 37.45 | 0 |
|  |  |  |  |  |  |  |  |  |  |  |  |  |  |  |  |  |  |  |  |  |  |  |  |  |  |  |  |  |  |

**Table S1B**. Identification details of differentiable proteins in the mesocarp of untreated, 1-MCP and O_3_ treated apples (cv. Granny Smith) inoculated/or not with the pathogen *B. cinerea* after of 60 days of cold storage.

| Spot N^o^ **^a^** | Accession Number **^b^** | Suggested Name **^c^** | Matching criteria **^d^** | Organism **^e^** | Subcellular localization  **f** | Functional category **^g^** | Mascot Score | MS Coverage | Protein MW | pI-Value | Sequence | # Peptides |
| --- | --- | --- | --- | --- | --- | --- | --- | --- | --- | --- | --- | --- |
| 50 | tr\|Q8W2C8\|Q8W2C | Sorbitol dehydrogenase |  | *Malus domestica* | Cytoplasm | 11.05-  Disease/Defense/Stress responses | 40 | 21 | 40152 | 8.9 | ENMAAWLVDVNTIK | 9 |
|  |  |  |  |  |  |  |  |  |  |  | ENMAAWLVDVNTIK |  |
|  |  |  |  |  |  |  |  |  |  |  | AVGICGSDVHYLK |  |
|  |  |  |  |  |  |  |  |  |  |  | AVGICGSDVHYLKTMK |  |
|  |  |  |  |  |  |  |  |  |  |  | SLGADGTVKVSR |  |
|  |  |  |  |  |  |  |  |  |  |  | EVDVVGVFRCK |  |
|  |  |  |  |  |  |  |  |  |  |  | CKNTWPLCLEFLR |  |
|  |  |  |  |  |  |  |  |  |  |  | NTWPLCLEFLRSGK |  |
|  |  |  |  |  |  |  |  |  |  |  | GGDAIKVMFNL |  |
| 109 | tr\|M5Y2Z8\|M5Y2Z | Serine/threonine-  protein phosphatase |  | *Prunus persica* | Cytoplasm | 10.04-Signal transduction/Kinases | 72 | 32 | 35442 | 4.8 | YPAHITLLR | 8 |
|  |  |  |  |  |  |  |  |  |  |  | QLTQVYGFYDECQR |  |
|  |  |  |  |  |  |  |  |  |  |  | YGNANAWR |  |
|  |  |  |  |  |  |  |  |  |  |  | GAGWLFGSR |  |
|  |  |  |  |  |  |  |  |  |  |  | VTSEFNHINNLDLVCR |  |
|  |  |  |  |  |  |  |  |  |  |  | GLVTVWSAPNYCYR |  |
|  |  |  |  |  |  |  |  |  |  |  | CGNVASILSFNDNMER |  |
|  |  |  |  |  |  |  |  |  |  |  | FFTETEENNQMR |  |
| 205 | tr\|A0A0D4CZ16\|A | CYP38 |  | *Malus domestica* | Chloroplast | 06.01-Protein destination and storage/Folding and  Stabillity | 45 | 19 | 48569 | 4.8 | LEAGMNELQQIVEDR | 7 |
|  |  |  |  |  |  |  |  |  |  |  | VKDNPSLTDCVFR |  |
|  |  |  |  |  |  |  |  |  |  |  | DNPSLTDCVFR |  |
|  |  |  |  |  |  |  |  |  |  |  | IVLDGYNAPVTAGNFVDL  VER |  |
|  |  |  |  |  |  |  |  |  |  |  | HFYDGMEIQR |  |
|  |  |  |  |  |  |  |  |  |  |  | TIPLEIMVEGEK |  |
|  |  |  |  |  |  |  |  |  |  |  | ESELTPSNSNILDGR |  |

| 313 | tr\|M5XW60\|M5XW6 | Transadolase | 100% to Prunus  mume gi\|645231139 E-value: 0.0 | *Malus domestica* | Plastid | 02.07-Energy/Pentose phosphate | 79 | 26 | 48087 | 6.4 | TYLHDLYEK | 10 |
| --- | --- | --- | --- | --- | --- | --- | --- | --- | --- | --- | --- | --- |
|  |  |  |  |  |  |  |  |  |  |  | GVTSNPAIFQK |  |
|  |  |  |  |  |  |  |  |  |  |  | AISTSNAYNDQFR |  |
|  |  |  |  |  |  |  |  |  |  |  | VVARPNVYIK |  |
|  |  |  |  |  |  |  |  |  |  |  | VTSVASFFVSR |  |
|  |  |  |  |  |  |  |  |  |  |  | IGTPEALDLR |  |
|  |  |  |  |  |  |  |  |  |  |  | AAVAQAALAYQLYQK |  |
|  |  |  |  |  |  |  |  |  |  |  | LLWASTSVK |  |
|  |  |  |  |  |  |  |  |  |  |  | TIDSNVSEAEGIYSALEK |  |
|  |  |  |  |  |  |  |  |  |  |  | SFDSLLDTLQEK |  |
| 625 | gi\|657973280 | Protein disulfide- isomerase |  | *Malus domestica* | Endoplasmic reticulum | 06.01-Protein destination and storage/Folding and  Stabillity | 88 | 14 | 56261 | 4.8 | TIQEYKGPR | 9 |
|  |  |  |  |  |  |  |  |  |  |  | EAEGIVEYLK |  |
|  |  |  |  |  |  |  |  |  |  |  | EAEGIVEYLKK |  |
|  |  |  |  |  |  |  |  |  |  |  | SDYEFGHTLDAK |  |
|  |  |  |  |  |  |  |  |  |  |  | GDSSVSGPVVR |  |
|  |  |  |  |  |  |  |  |  |  |  | FFNSPNEK |  |
|  |  |  |  |  |  |  |  |  |  |  | YYPTLYFK |  |
|  |  |  |  |  |  |  |  |  |  |  | VLSYDEEDR |  |
|  |  |  |  |  |  |  |  |  |  |  | VLSYDEEDRTK |  |
| 1037 | tr\|Q4VPK5\|Q4VPK | Allergen |  | *Malus domestica* | Cytoplasm | 11.05-  Disease/Defense/Stress responses | 83 | 58 | 17416 | 5.1 | GVLTYETEYASVIPPAR | 8 |
|  |  |  |  |  |  |  |  |  |  |  | LYNALVLDADNLIPK |  |
|  |  |  |  |  |  |  |  |  |  |  | TVEILEGDGGVGTIK |  |
|  |  |  |  |  |  |  |  |  |  |  | KVSFGEGSEYSYVK |  |
|  |  |  |  |  |  |  |  |  |  |  | VSFGEGSEYSYVK |  |
|  |  |  |  |  |  |  |  |  |  |  | DNFDYSYSLIEGDAISDK |  |
|  |  |  |  |  |  |  |  |  |  |  | ISYEIK |  |
|  |  |  |  |  |  |  |  |  |  |  | NTSHYHTK |  |
| 1037 | tr\|B0B0L6\|B0B0L | Major allergen |  | *Malus domestica* | Cytoplasm | 11.02-  Disease/defense/Defens e-related | 68 | 48 | 17440 | 5.1 | LYNALVLDADNLIPK | 7 |
|  |  |  |  |  |  |  |  |  |  |  | TVEILEGDGGVGTIK |  |
|  |  |  |  |  |  |  |  |  |  |  | KVSFGEGSEYSYVK |  |
|  |  |  |  |  |  |  |  |  |  |  | VSFGEGSEYSYVK |  |
|  |  |  |  |  |  |  |  |  |  |  | DNFDYSYSLIEGDAISDK |  |
|  |  |  |  |  |  |  |  |  |  |  | ISYEIK |  |

|  |  |  |  |  |  |  |  |  |  |  | NTSHYHTK |  |
| --- | --- | --- | --- | --- | --- | --- | --- | --- | --- | --- | --- | --- |
| 1132 | tr\|R4I6H0\|R4I6H | ACC oxidase |  | *Malus domestica* | Cytoplasm | 11.05-  Disease/Defense/Stress responses | 41 | 23 | 34274 | 5 | GLDDVQSEIHDLDWESTFF LR | 5 |
|  |  |  |  |  |  |  |  |  |  |  | HLPSSNISEIPDLEEEYR |  |
|  |  |  |  |  |  |  |  |  |  |  | HLPSSNISEIPDLEEEYRK |  |
|  |  |  |  |  |  |  |  |  |  |  | VSNYPPCPKPDLIK |  |
|  |  |  |  |  |  |  |  |  |  |  | AHSDAGGIILLFQDDK |  |
| 1212 | tr\|R4I6H0\|R4I6H | ACC oxidase |  | *Malus domestica* | Cytoplasm | 11.05-  Disease/Defense/Stress responses | 83 | 41 | 34274 | 5 | GLDDVQSEIHDLDWESTFF LR | 9 |
|  |  |  |  |  |  |  |  |  |  |  | HLPSSNISEIPDLEEEYR |  |
|  |  |  |  |  |  |  |  |  |  |  | HLPSSNISEIPDLEEEYRK |  |
|  |  |  |  |  |  |  |  |  |  |  | LLDLLCENLGLEK |  |
|  |  |  |  |  |  |  |  |  |  |  | VSNYPPCPKPDLIK |  |
|  |  |  |  |  |  |  |  |  |  |  | AHSDAGGIILLFQDDK |  |
|  |  |  |  |  |  |  |  |  |  |  | VIAQSDGTR |  |
|  |  |  |  |  |  |  |  |  |  |  | MSIASFYNPGNDSFISPAPA  VLEK Oxidation (M) |  |
|  |  |  |  |  |  |  |  |  |  |  | FVFDDYMK Oxidation (M) |  |
| 1220 | tr\|R4I6H0\|R4I6H | ACC oxidase |  | *Malus domestica* | Cytoplasm | 11.05-  Disease/Defense/Stress responses | 60 | 35 | 34274 | 5 | GLDDVQSEIHDLDWESTFF LR | 7 |
|  |  |  |  |  |  |  |  |  |  |  | HLPSSNISEIPDLEEEYR |  |
|  |  |  |  |  |  |  |  |  |  |  | HLPSSNISEIPDLEEEYRK |  |
|  |  |  |  |  |  |  |  |  |  |  | LLDLLCENLGLEK |  |
|  |  |  |  |  |  |  |  |  |  |  | VSNYPPCPKPDLIK |  |
|  |  |  |  |  |  |  |  |  |  |  | AHSDAGGIILLFQDDK |  |
|  |  |  |  |  |  |  |  |  |  |  | MSIASFYNPGNDSFISPAPA  VLEK |  |
| 1507 | tr\|J9PZL0\|J9PZL | Dehydrin 8 |  | *Malus domestica* | Nucleus | 11.05-  Disease/Defense/Stress responses | 52 | 25 | 32802 | 5.1 | SQEHEYER | 19 |
|  |  |  |  |  |  |  |  |  |  |  | GLFDFLGK |  |
|  |  |  |  |  |  |  |  |  |  |  | ISDHHEALAPDQYTSSYSK |  |
|  |  |  |  |  |  |  |  |  |  |  | VYEEEHHHQAPAPVVHHH  EEPTDYPTEEK |  |
|  |  |  |  |  |  |  |  |  |  |  | LPGYHSK |  |
| 1510 | tr\|A0A0B4S3Q7\|A | V-ATPase |  | *Eriobotrya japonica* | Chloroplast | 07.22-  Transporters/Transport ATPases | 70 | 41 | 54667 | 4.89 | TVSGVAGPLVILEK | 14 |

|  |  |  |  |  |  |  |  |  |  |  | AIVQVFEGTSGIDNK |  |
| --- | --- | --- | --- | --- | --- | --- | --- | --- | --- | --- | --- | --- |
|  |  |  |  |  |  |  |  |  |  |  | YTTVQFTGEVLK |  |
|  |  |  |  |  |  |  |  |  |  |  | IFNGSGKPIDNGPPILPEAY  LDISGSSINPSER |  |
|  |  |  |  |  |  |  |  |  |  |  | TYPEEMIQTGISTIDVMNSI  AR |  |
|  |  |  |  |  |  |  |  |  |  |  | TYPEEMIQTGISTIDVMNSI  AR Oxidation (M) |  |
|  |  |  |  |  |  |  |  |  |  |  | IPLFSAAGLPHNEIAAQICR |  |
|  |  |  |  |  |  |  |  |  |  |  | VTLFLNLANDPTIER |  |
|  |  |  |  |  |  |  |  |  |  |  | IITPRIALTTAEYLAYECGK |  |
|  |  |  |  |  |  |  |  |  |  |  | GYPGYMYTDLAQIYER |  |
|  |  |  |  |  |  |  |  |  |  |  | GYPGYMYTDLAQIYER  Oxidation (M) |  |
|  |  |  |  |  |  |  |  |  |  |  | QIYPPINVLPSLSR |  |
|  |  |  |  |  |  |  |  |  |  |  | RDHSDVSNQLYANYAIGK |  |
|  |  |  |  |  |  |  |  |  |  |  | DHSDVSNQLYANYAIGK |  |
| 1511 | tr\|M5VIV9\|M5VIV | V-ATPase | 100% to Prunus mume gi\|645260938  E-value: 0.0 | *Prunus persica* | Chloroplast | 07.22-  Transporters/Transport ATPases | 109 | 48 | 54622 | 4.8 | TVSGVAGPLVILEK | 17 |
|  |  |  |  |  |  |  |  |  |  |  | AIVQVFEGTSGIDNK |  |
|  |  |  |  |  |  |  |  |  |  |  | TVQFTGEVLK |  |
|  |  |  |  |  |  |  |  |  |  |  | IFNGSGKPIDNGPPILPEAY  LDISGSSINPSER |  |
|  |  |  |  |  |  |  |  |  |  |  | TYPEEMIQTGISTIDVMNSI  AR |  |
|  |  |  |  |  |  |  |  |  |  |  | TYPEEMIQTGISTIDVMNSI  AR Oxidation (M) |  |
|  |  |  |  |  |  |  |  |  |  |  | IPLFSAAGLPHNEIAAQICR |  |
|  |  |  |  |  |  |  |  |  |  |  | VTLFLNLANDPTIER |  |
|  |  |  |  |  |  |  |  |  |  |  | IITPRIALTTAEYLAYECGK |  |
|  |  |  |  |  |  |  |  |  |  |  | IALTTAEYLAYECGK |  |
|  |  |  |  |  |  |  |  |  |  |  | HVLVILTDMSSYADALR  Oxidation (M) |  |
|  |  |  |  |  |  |  |  |  |  |  | GYPGYMYTDLAQIYER |  |

|  |  |  |  |  |  |  |  |  |  |  | GYPGYMYTDLAQIYER  Oxidation (M) |  |
| --- | --- | --- | --- | --- | --- | --- | --- | --- | --- | --- | --- | --- |
|  |  |  |  |  |  |  |  |  |  |  | QIYPPINVLPSLSR |  |
|  |  |  |  |  |  |  |  |  |  |  | RDHSDVSNQLYANYAIGK |  |
|  |  |  |  |  |  |  |  |  |  |  | DHSDVSNQLYANYAIGK |  |
|  |  |  |  |  |  |  |  |  |  |  | AVVGEEALSSEDLLYLEFL  DKFEK |  |
| 1725 | tr\|Q9M6R1\|Q9M6R | HSP High MW |  | *Malus domestica* | Endoplasmic reticulum | 06.01-Protein destination and storage/Folding and  Stabillity | 133 | 47 | 71570 | 5 | GEGPAIGIDLGTTYSCVGV WQHDR | 22 |
|  |  |  |  |  |  |  |  |  |  |  | VEIIANDQGNR |  |
|  |  |  |  |  |  |  |  |  |  |  | TTPSYVAFTDTER |  |
|  |  |  |  |  |  |  |  |  |  |  | NQVAMNPVNTVFDAK |  |
|  |  |  |  |  |  |  |  |  |  |  | QFAAEEISSMVLVKMR 2  Oxidation (M) |  |
|  |  |  |  |  |  |  |  |  |  |  | EIAEAYLGSSIK |  |
|  |  |  |  |  |  |  |  |  |  |  | NAVVTVPAYFNDSQR |  |
|  |  |  |  |  |  |  |  |  |  |  | DAGVIAGLNVLR |  |
|  |  |  |  |  |  |  |  |  |  |  | IINEPTAAAIAYGLDK |  |
|  |  |  |  |  |  |  |  |  |  |  | IINEPTAAAIAYGLDKK |  |
|  |  |  |  |  |  |  |  |  |  |  | ATAGDTHLGGEDFDNR |  |
|  |  |  |  |  |  |  |  |  |  |  | MVNHFVQEFK |  |
|  |  |  |  |  |  |  |  |  |  |  | TLSSTAQTTIEIDSLYEGVD  FYSTITR |  |
|  |  |  |  |  |  |  |  |  |  |  | ARFEELNMDLFR |  |
|  |  |  |  |  |  |  |  |  |  |  | FEELNMDLFR |  |
|  |  |  |  |  |  |  |  |  |  |  | STVHDVVLVGGSTR |  |
|  |  |  |  |  |  |  |  |  |  |  | VQQLLQDFFNGK |  |
|  |  |  |  |  |  |  |  |  |  |  | SINPDEAVAYGAAVQAAIL  SGEGNEK |  |
|  |  |  |  |  |  |  |  |  |  |  | EQVFSTYSDNQPGVLIQVY  EGER |  |
|  |  |  |  |  |  |  |  |  |  |  | FELSGIPPAPR |  |
|  |  |  |  |  |  |  |  |  |  |  | NALENYAYNMR |  |
|  |  |  |  |  |  |  |  |  |  |  | ELESICNPIIAK |  |
| 2029 | tr\|Q4VPK5\|Q4VPK | Allergen |  | *Malus domestica* | Cytoplasm | 11.05-  Disease/Defense/Stress responses | 81 | 51 | 17416 | 5.1 | GVLTYETEYASVIPPAR | 7 |
|  |  |  |  |  |  |  |  |  |  |  | LYNALVLDADNLIPK |  |
|  |  |  |  |  |  |  |  |  |  |  | TVEILEGDGGVGTIK |  |

|  |  |  |  |  |  |  |  |  |  |  | KVSFGEGSEYSYVK |  |
| --- | --- | --- | --- | --- | --- | --- | --- | --- | --- | --- | --- | --- |
|  |  |  |  |  |  |  |  |  |  |  | VSFGEGSEYSYVK |  |
|  |  |  |  |  |  |  |  |  |  |  | DNFDYSYSLIEGDAISDK |  |
|  |  |  |  |  |  |  |  |  |  |  | DNFDYSYSLIEGDAISDKIE  K |  |
| 2032 | gi\|658024705 | Glycine-rich RNA- binding protein |  | *Malus domestica* | Nucleus | 11.05-  Disease/Defense/Stress responses | 89 | 52 | 16877 | 5.5 | CFVGGLAWATDNEALER | 9 |
|  |  |  |  |  |  |  |  |  |  |  | AFSQYGEIIESK |  |
|  |  |  |  |  |  |  |  |  |  |  | GFGFVTFGNEQAMR |  |
|  |  |  |  |  |  |  |  |  |  |  | GFGFVTFGNEQAMR  Oxidation (M) |  |
|  |  |  |  |  |  |  |  |  |  |  | DAIEAMNGQNLDGR |  |
|  |  |  |  |  |  |  |  |  |  |  | DAIEAMNGQNLDGR  Oxidation (M) |  |
|  |  |  |  |  |  |  |  |  |  |  | NITVNEAQSR |  |
|  |  |  |  |  |  |  |  |  |  |  | EGGYGGGEGGYSR |  |
|  |  |  |  |  |  |  |  |  |  |  | GGGGAEGGSWR |  |
| 2110 | gi\|658011365 | Stem-specific  protein |  | *Malus domestica* | Cytoplasm | 12-Unclear  classification | 50 | 26 | 25208 | 5.3 | DEIFCLFEGALDNLGSLR | 4 |
|  |  |  |  |  |  |  |  |  |  |  | SANEVILVIEAYK |  |
|  |  |  |  |  |  |  |  |  |  |  | STSTVFVASDQFGK |  |
|  |  |  |  |  |  |  |  |  |  |  | ITAVPATDEEIWGATFK |  |
| 2115 | gi\|657960718 | Plasma membrane- associated cation-  binding protein |  | *Malus domestica* | Plasma membrane | 12-Unclear classification | 53 | 32 | 23495 | 5 | VIELYEASSAEIK | 4 |
|  |  |  |  |  |  |  |  |  |  |  | YGSAYVSGPVFFVFEK |  |
|  |  |  |  |  |  |  |  |  |  |  | VSTFIVTEETVEPLPPPAEA  TK |  |
|  |  |  |  |  |  |  |  |  |  |  | KPKPTEPEAAAEAPPPK |  |
| 2213 | tr\|R4I6H0\|R4I6H | ACC oxidase |  | *Malus domestica* | Cytoplasm | 11.05-  Disease/Defense/Stress responses | 77 | 28 | 34274 | 5 | GLDDVQSEIHDLDWESTFF LR | 7 |
|  |  |  |  |  |  |  |  |  |  |  | HLPSSNISEIPDLEEEYR |  |
|  |  |  |  |  |  |  |  |  |  |  | HLPSSNISEIPDLEEEYRK |  |
|  |  |  |  |  |  |  |  |  |  |  | VSNYPPCPKPDLIK |  |
|  |  |  |  |  |  |  |  |  |  |  | AHSDAGGIILLFQDDK |  |
|  |  |  |  |  |  |  |  |  |  |  | AHSDAGGIILLFQDDKVSG  LQLLK |  |
|  |  |  |  |  |  |  |  |  |  |  | FVFDDYMK |  |

| 2320 | sp\|Q1KLZ2\|CAS1_ | L-3-cyanoalanine synthase |  | *Malus domestica* | Mitochondrion | 01.01-  Metabolism/Amino Acid | 102 | 39 | 41085 | 8.7 | TQVSQLIGR | 11 |
| --- | --- | --- | --- | --- | --- | --- | --- | --- | --- | --- | --- | --- |
|  |  |  |  |  |  |  |  |  |  |  | DRPALSMINDAEEK |  |
|  |  |  |  |  |  |  |  |  |  |  | GYKMVLTMPSYTSLER |  |
|  |  |  |  |  |  |  |  |  |  |  | MVLTMPSYTSLER |  |
|  |  |  |  |  |  |  |  |  |  |  | CFGADLILTDPTK |  |
|  |  |  |  |  |  |  |  |  |  |  | AYDLLESTPNAYMLQQFS  NPANTK |  |
|  |  |  |  |  |  |  |  |  |  |  | VIEVTSEDAVNMAR |  |
|  |  |  |  |  |  |  |  |  |  |  | EGLMVGISSGANTVAAME  LAK |  |
|  |  |  |  |  |  |  |  |  |  |  | GKLIVTVHPSFGER |  |
|  |  |  |  |  |  |  |  |  |  |  | LIVTVHPSFGER |  |
|  |  |  |  |  |  |  |  |  |  |  | YLSSVLFQELRQEAENMQ  PVAVDYP Oxidation (M) |  |
| 2326 | tr\|M5W4J8\|M5W4J | 40S ribosomal protein |  | *Malus domestica* | Cytosol | 05.01-Protein synthesis/Ribosomal  proteins | 63 | 22 | 32506 | 5.2 | NCDFQMER | 5 |
|  |  |  |  |  |  |  |  |  |  |  | FAQHTGANAIAGR |  |
|  |  |  |  |  |  |  |  |  |  |  | HTPGTFTNQLQTSFNEPR |  |
|  |  |  |  |  |  |  |  |  |  |  | LLILTDPR |  |
|  |  |  |  |  |  |  |  |  |  |  | EAALGNIPTIAFCDTDSPM  R |  |
| 2634 | tr\|M5W737\|M5W73 | RuBisCO large subunit-binding protein subunit  beta | 100% to Prunus mume gi\|645215194  E-value: 0.0 | *Prunus mume* | Chloroplast | 06.01-Protein destination and storage/Folding and  Stabillity | 51 | 13 | 64778 | 5.7 | MASTFAAMTSVGSLAAPS SR | 5 |
|  |  |  |  |  |  |  |  |  |  |  | SAENSLYVVEGMQFDR |  |
|  |  |  |  |  |  |  |  |  |  |  | DLINILEEAIR |  |
|  |  |  |  |  |  |  |  |  |  |  | SQYLDDIAILTGGTVIR |  |
|  |  |  |  |  |  |  |  |  |  |  | AAVEEGIVVGGGCTLLR |  |
| 2636 | tr\|A0A023IRP7\|A | Malic enzyme |  | *Malus domestica* | Mitochondrion | 02.10-Energy/TCA  pathway | 74 | 25 | 66495 | 5.1 | GTSFSFTER | 12 |
|  |  |  |  |  |  |  |  |  |  |  | GLLPPNVMSTEHQIER |  |
|  |  |  |  |  |  |  |  |  |  |  | RLEEQVR |  |
|  |  |  |  |  |  |  |  |  |  |  | GMYFSAEDR |  |
|  |  |  |  |  |  |  |  |  |  |  | LDLYVAAAGINPQR |  |
|  |  |  |  |  |  |  |  |  |  |  | MFNDDVQGTAGVALAGL  LGAVR |  |
|  |  |  |  |  |  |  |  |  |  |  | IVVAGAGSAGIGVLNAAR |  |

|  |  |  |  |  |  |  |  |  |  |  | MLGNNEDAFQSAGR |  |
| --- | --- | --- | --- | --- | --- | --- | --- | --- | --- | --- | --- | --- |
|  |  |  |  |  |  |  |  |  |  |  | QFWVVDAK |  |
|  |  |  |  |  |  |  |  |  |  |  | EDIDPDARPFAR |  |
|  |  |  |  |  |  |  |  |  |  |  | GIIYPSISSIR |  |
|  |  |  |  |  |  |  |  |  |  |  | EAIEEDLAEGYR |  |
| 2727 | tr\|B3SGC7\|B3SGC | V-ATPase |  | *Malus domestica* | Vacuole | 07.22-  Transporters/Transport ATPases | 231 | 55 | 69161 | 5.2 | LTTFEDSEKESEYGYVR | 28 |
|  |  |  |  |  |  |  |  |  |  |  | ESEYGYVR |  |
|  |  |  |  |  |  |  |  |  |  |  | VSGPVVVADGMAGAAMY  ELVR |  |
|  |  |  |  |  |  |  |  |  |  |  | VGHDNLIGEIIR |  |
|  |  |  |  |  |  |  |  |  |  |  | LEGDSATIQVYEETAGLM  VNDPVLR |  |
|  |  |  |  |  |  |  |  |  |  |  | SGDVYIPR |  |
|  |  |  |  |  |  |  |  |  |  |  | GVSVPALDKDILWEFQPK |  |
|  |  |  |  |  |  |  |  |  |  |  | IGEGDHLTGGDLYATVFE  NSLMEHR |  |
|  |  |  |  |  |  |  |  |  |  |  | DTVLELEFQGQK |  |
|  |  |  |  |  |  |  |  |  |  |  | FTMLQTWPVR |  |
|  |  |  |  |  |  |  |  |  |  |  | TPRPVASK |  |
|  |  |  |  |  |  |  |  |  |  |  | LAADTPLLTGQR |  |
|  |  |  |  |  |  |  |  |  |  |  | YSNSDTVVYVGCGER |  |
|  |  |  |  |  |  |  |  |  |  |  | TTLVANTSNMPVAAR |  |
|  |  |  |  |  |  |  |  |  |  |  | EASIYTGITIAEYFR |  |
|  |  |  |  |  |  |  |  |  |  |  | DMGYNVSMMADSTSR |  |
|  |  |  |  |  |  |  |  |  |  |  | LAEMPADSGYPAYLAAR |  |
|  |  |  |  |  |  |  |  |  |  |  | LAEMPADSGYPAYLAAR  Oxidation (M) |  |
|  |  |  |  |  |  |  |  |  |  |  | LASFYER |  |
|  |  |  |  |  |  |  |  |  |  |  | VKCLGGPER |  |
|  |  |  |  |  |  |  |  |  |  |  | YSTALESFYDQFDPDFINIR |  |
|  |  |  |  |  |  |  |  |  |  |  | EDDLNEIVQLVGK |  |
|  |  |  |  |  |  |  |  |  |  |  | EDYLAQNAFTPYDK |  |
|  |  |  |  |  |  |  |  |  |  |  | SVWMMR |  |
|  |  |  |  |  |  |  |  |  |  |  | NIIHYFNLANQAVER |  |
|  |  |  |  |  |  |  |  |  |  |  | LGDLFYR |  |
|  |  |  |  |  |  |  |  |  |  |  | KLHEDLTSGFR |  |
|  |  |  |  |  |  |  |  |  |  |  | LHEDLTSGFR |  |

| 2839 | tr\|M5WX60\|M5WX6 | HSP 70 | 98% to Prunus mume gi\|645244630 E-  value: 0.0 | *Prunus mume* | Mitochondrion | 06.01-Protein destination and storage/Folding and  Stabillity | 79 | 26 | 95449 | 5 | FIGTAGAASSLMNPKNTIS QIK | 16 |
| --- | --- | --- | --- | --- | --- | --- | --- | --- | --- | --- | --- | --- |
|  |  |  |  |  |  |  |  |  |  |  | NLNAAVVDCCIGIPVYFTD  LQR |  |
|  |  |  |  |  |  |  |  |  |  |  | LFHETTATALAYGIYK |  |
|  |  |  |  |  |  |  |  |  |  |  | ILAHSFDQSLGGR |  |
|  |  |  |  |  |  |  |  |  |  |  | DFDEVLFHHFAAK |  |
|  |  |  |  |  |  |  |  |  |  |  | KMLSANPEAPLNIECLMEE  K 2 Oxidation (M) |  |
|  |  |  |  |  |  |  |  |  |  |  | GCALQCAILSPTFK |  |
|  |  |  |  |  |  |  |  |  |  |  | SGTFSVDVQYADVSDLQA  PAK |  |
|  |  |  |  |  |  |  |  |  |  |  | AIEKEFEMALQDR |  |
|  |  |  |  |  |  |  |  |  |  |  | NAVEAYVYDMR |  |
|  |  |  |  |  |  |  |  |  |  |  | LQEVEDWLYEDGEDETK |  |
|  |  |  |  |  |  |  |  |  |  |  | LQEVEDWLYEDGEDETKG  VYIAK |  |
|  |  |  |  |  |  |  |  |  |  |  | GTVIDQLGYCINSYR |  |
|  |  |  |  |  |  |  |  |  |  |  | VLNECVEAEAWLR |  |
|  |  |  |  |  |  |  |  |  |  |  | VLNECVEAEAWLREK |  |
|  |  |  |  |  |  |  |  |  |  |  | YANPVLLSADVR |  |
| 3040 | tr\|Q941P6\|Q941P | Major allergen |  | *Malus domestica* | Unclear | 11.02-  Disease/defense/Defens e-related | 65 | 62 | 17694 | 5.6 | GVYTFENEYTSEIPPPR | 7 |
|  |  |  |  |  |  |  |  |  |  |  | AFVLDADNLIPK |  |
|  |  |  |  |  |  |  |  |  |  |  | QAEILEGNGGPGTIK |  |
|  |  |  |  |  |  |  |  |  |  |  | KITFGEGSQYGYVK |  |
|  |  |  |  |  |  |  |  |  |  |  | ITFGEGSQYGYVK |  |
|  |  |  |  |  |  |  |  |  |  |  | IDSIDEASYSYSYTLIEGDA  LTDTIEK |  |
|  |  |  |  |  |  |  |  |  |  |  | LIESYLKDHPDAYN |  |
| 3220 | tr\|G5ELM7\|G5ELM | Actin |  | *Malus domestica* | Cytoskeleton | 09.04-Cell  structure/Cytoskeleton | 101 | 47 | 40369 | 5.6 | HTGVMVGMGQK 2  Oxidation (M) | 13 |
|  |  |  |  |  |  |  |  |  |  |  | YPIEHGIVSNWDDMEK |  |
|  |  |  |  |  |  |  |  |  |  |  | IWHHTFYNELR |  |
|  |  |  |  |  |  |  |  |  |  |  | VAPEEHPVLLTEAPLNPK |  |
|  |  |  |  |  |  |  |  |  |  |  | TTGIVLDSGDGVSHTVPIY  EGYALPHAILR |  |
|  |  |  |  |  |  |  |  |  |  |  | LDLAGRDLTDSLMK |  |

|  |  |  |  |  |  |  |  |  |  |  | LAYVALDYEQELETAK |  |
| --- | --- | --- | --- | --- | --- | --- | --- | --- | --- | --- | --- | --- |
|  |  |  |  |  |  |  |  |  |  |  | SSSSVEKNYELPDGQVITIG  AER |  |
|  |  |  |  |  |  |  |  |  |  |  | NYELPDGQVITIGAER |  |
|  |  |  |  |  |  |  |  |  |  |  | KDLYGNIVLSGGSTMFPGI  ADR |  |
|  |  |  |  |  |  |  |  |  |  |  | DLYGNIVLSGGSTMFPGIA  DR |  |
|  |  |  |  |  |  |  |  |  |  |  | DLYGNIVLSGGSTMFPGIA  DR Oxidation (M) |  |
|  |  |  |  |  |  |  |  |  |  |  | GEYDESGPSIVHR |  |
| 3220 | tr\|R4I6H0\|R4I6H | ACC oxidase |  | *Malus domestica* | Cytoplasm | 11.05-  Disease/Defense/Stress responses | 67 | 35 | 34274 | 4.98 | EMVAAKGLDDVQSEIHDL DWESTFFLR | 8 |
|  |  |  |  |  |  |  |  |  |  |  | GLDDVQSEIHDLDWESTFF  LR |  |
|  |  |  |  |  |  |  |  |  |  |  | HLPSSNISEIPDLEEEYR |  |
|  |  |  |  |  |  |  |  |  |  |  | HLPSSNISEIPDLEEEYRK |  |
|  |  |  |  |  |  |  |  |  |  |  | VSNYPPCPKPDLIK |  |
|  |  |  |  |  |  |  |  |  |  |  | VSNYPPCPKPDLIKGLR |  |
|  |  |  |  |  |  |  |  |  |  |  | AHSDAGGIILLFQDDK |  |
|  |  |  |  |  |  |  |  |  |  |  | MSIASFYNPGNDSFISPAPA  VLEKK Oxidation (M) |  |
| 3324 | sp\|Q1KLZ1\|CAS2_ | L-3-cyanoalanine synthase |  | *Malus domestica* | Mytochondrion | 01.01-  Metabolism/Amino Acid | 87 | 31 | 40688 | 8.8 | TQVSQLIGR | 9 |
|  |  |  |  |  |  |  |  |  |  |  | VTEGCGAFIAVK |  |
|  |  |  |  |  |  |  |  |  |  |  | QEMFQPTASIK |  |
|  |  |  |  |  |  |  |  |  |  |  | DRPALSMINDAEEK |  |
|  |  |  |  |  |  |  |  |  |  |  | MVLTMPSYTSLER |  |
|  |  |  |  |  |  |  |  |  |  |  | CFGADLILTDPTK |  |
|  |  |  |  |  |  |  |  |  |  |  | AYDLLESTPNAFMLQQFS  NPANTK |  |
|  |  |  |  |  |  |  |  |  |  |  | LIVTVHASFGER |  |
|  |  |  |  |  |  |  |  |  |  |  | YLSSVLFQDLR |  |
| 3327 | tr\|G5ELM7\|G5ELM | Actin |  | *Malus domestica* | Cytoskeleton | 09.04-Cell  structure/Cytoskeleton | 65 | 34 | 40369 | 5.6 | AGFAGDDAPR | 10 |
|  |  |  |  |  |  |  |  |  |  |  | HTGVMVGMGQK 2  Oxidation (M) |  |
|  |  |  |  |  |  |  |  |  |  |  | VAPEEHPVLLTEAPLNPK |  |
|  |  |  |  |  |  |  |  |  |  |  | EIVRDMK Oxidation (M) |  |

|  |  |  |  |  |  |  |  |  |  |  | LAYVALDYEQELETAK |  |
| --- | --- | --- | --- | --- | --- | --- | --- | --- | --- | --- | --- | --- |
|  |  |  |  |  |  |  |  |  |  |  | SSSSVEKNYELPDGQVITIG  AER |  |
|  |  |  |  |  |  |  |  |  |  |  | NYELPDGQVITIGAER |  |
|  |  |  |  |  |  |  |  |  |  |  | DLYGNIVLSGGSTMFPGIA  DR |  |
|  |  |  |  |  |  |  |  |  |  |  | VVAPPER |  |
|  |  |  |  |  |  |  |  |  |  |  | GEYDESGPSIVHR |  |
| 3719 | sp\|P48981\|BGAL_ | Beta-galactosidase |  | *Malus domestica* | Apoplast | 01.05-  Metabolism/Sugars and polysaccharides | 57 | 11 | 81628 | 5.6 | ILISGSIHYPR | 8 |
|  |  |  |  |  |  |  |  |  |  |  | LVQQEGLFVNLR |  |
|  |  |  |  |  |  |  |  |  |  |  | YVPGIAFR |  |
|  |  |  |  |  |  |  |  |  |  |  | SESDCAAFLANYDAK |  |
|  |  |  |  |  |  |  |  |  |  |  | LSFSQNVNLR |  |
|  |  |  |  |  |  |  |  |  |  |  | GQIWINGQSVGR |  |
|  |  |  |  |  |  |  |  |  |  |  | HWPGYIAR |  |
|  |  |  |  |  |  |  |  |  |  |  | WYHIPR |  |
| 4016 | sp\|Q40280\|MAL12 | Major allergen |  | *Malus domestica* | Unclear | 11.02-  Disease/defense/Defens e-related | 85 | 49 | 17528 | 5.6 | GVYTFENEYTSEIPPPR | 8 |
|  |  |  |  |  |  |  |  |  |  |  | GVYTFENEYTSEIPPPRLFK |  |
|  |  |  |  |  |  |  |  |  |  |  | HAEILEGDGGPGTIK |  |
|  |  |  |  |  |  |  |  |  |  |  | HAEILEGDGGPGTIKK |  |
|  |  |  |  |  |  |  |  |  |  |  | KITFGEGSQYGYVK |  |
|  |  |  |  |  |  |  |  |  |  |  | ITFGEGSQYGYVK |  |
|  |  |  |  |  |  |  |  |  |  |  | HKIDSVDEANYSYAYTLIE  GDALTDTIEK |  |
|  |  |  |  |  |  |  |  |  |  |  | IDSVDEANYSYAYTLIEGD  ALTDTIEK |  |
| 4026 | gi\|657979046 | 5'-3'  exoribonuclease |  | *Malus domestica* | Nucleus | 04.99-  transcription/Others | 51 | 34 | 22112 | 5.7 | YSETTAAYGSTTTHESELD  YK | 5 |
|  |  |  |  |  |  |  |  |  |  |  | YSETTAAYGSTTTHESELD  YKK |  |
|  |  |  |  |  |  |  |  |  |  |  | HLEHLGEAGVAAAGAFAL  HEK |  |
|  |  |  |  |  |  |  |  |  |  |  | HKIEEEIAAAAAVGSGGFA  FHEHHEK |  |
|  |  |  |  |  |  |  |  |  |  |  | IEEEIAAAAAVGSGGFAFH  EHHEK |  |

| 4105 | gi\|658000119 | Proteasome subunit alpha type |  | *Malus domestica* | Cytosol | 06.13-Protein destination and  storage/Proteolysis | 103 | 42 | 27638 | 5.7 | VFQIEYAAK | 10 |
| --- | --- | --- | --- | --- | --- | --- | --- | --- | --- | --- | --- | --- |
|  |  |  |  |  |  |  |  |  |  |  | MMLPGSNR |  |
|  |  |  |  |  |  |  |  |  |  |  | HSGMAVAGLAADGR |  |
|  |  |  |  |  |  |  |  |  |  |  | SEATNYESVYGEQIPVK |  |
|  |  |  |  |  |  |  |  |  |  |  | DGPQLYMVEPSGVSYR |  |
|  |  |  |  |  |  |  |  |  |  |  | DGPQLYMVEPSGVSYR  Oxidation (M) |  |
|  |  |  |  |  |  |  |  |  |  |  | LSELTCR |  |
|  |  |  |  |  |  |  |  |  |  |  | IIYGIHDEAK |  |
|  |  |  |  |  |  |  |  |  |  |  | EFELEMSWVCDESNR |  |
|  |  |  |  |  |  |  |  |  |  |  | VPDELLEEAK |  |
| 4313 | gi\|657982223 | Phosphoglycerate  kinase |  | *Malus domestica* | Chloroplast | 02.30-  Energy/Photosynthesis | 102 | 30 | 50159 | 8.7 | ADLNVPLDDSQNITDDTR | 9 |
|  |  |  |  |  |  |  |  |  |  |  | FSLAPLVPR |  |
|  |  |  |  |  |  |  |  |  |  |  | LVASLPEGGVLLLENVR |  |
|  |  |  |  |  |  |  |  |  |  |  | LAALADLYVNDAFGTAHR |  |
|  |  |  |  |  |  |  |  |  |  |  | FLRPSVAGFLLQK |  |
|  |  |  |  |  |  |  |  |  |  |  | RPFAAIVGGSK |  |
|  |  |  |  |  |  |  |  |  |  |  | IVPASSIPDGWMGLDIGPD  SIK |  |
|  |  |  |  |  |  |  |  |  |  |  | TIIWNGPMGVFEFDK |  |
|  |  |  |  |  |  |  |  |  |  |  | VGVASVMSHISTGGGASL  ELLEGK |  |
| 4314 | tr\|A0A0M4PFM6\|A | Glutamine synthetase |  | *Pyrus bretschneideri* | Cytosol | 01.01-  Metabolism/Amino acid | 75 | 28 | 39145 | 5.9 | KIIAEYIWIGGSGMDIR | 12 |
|  |  |  |  |  |  |  |  |  |  |  | KIIAEYIWIGGSGMDIR  Oxidation (M) |  |
|  |  |  |  |  |  |  |  |  |  |  | IIAEYIWIGGSGMDIR |  |
|  |  |  |  |  |  |  |  |  |  |  | IIAEYIWIGGSGMDIR  Oxidation (M) |  |
|  |  |  |  |  |  |  |  |  |  |  | TLPGPVSDPSKLPK |  |
|  |  |  |  |  |  |  |  |  |  |  | GNNILVICDTYTPAGEPIPT  NKR |  |
|  |  |  |  |  |  |  |  |  |  |  | SMREDGGYEVIK |  |
|  |  |  |  |  |  |  |  |  |  |  | HKEHIAAYGEGNER |  |
|  |  |  |  |  |  |  |  |  |  |  | EHIAAYGEGNER |  |
|  |  |  |  |  |  |  |  |  |  |  | LTGRHETADINTFK |  |

|  |  |  |  |  |  |  |  |  |  |  | HETADINTFK |  |
| --- | --- | --- | --- | --- | --- | --- | --- | --- | --- | --- | --- | --- |
|  |  |  |  |  |  |  |  |  |  |  | HETADINTFKWGVANR |  |
| 4517 | tr\|V9SI88\|V9SI8 | Glutamate decarboxylase |  | *Malus domestica* | Unclear | 01.01-  Metabolism/Amino acid | 95 | 33 | 56941 | 5.5 | GEQVNCTFASR | 16 |
|  |  |  |  |  |  |  |  |  |  |  | DSAYQIINDELMLDGNPR |  |
|  |  |  |  |  |  |  |  |  |  |  | DSAYQIINDELMLDGNPR  Oxidation (M) |  |
|  |  |  |  |  |  |  |  |  |  |  | LNLASFVTTWMEPECDR |  |
|  |  |  |  |  |  |  |  |  |  |  | NYVDMDEYPVTTELQNR |  |
|  |  |  |  |  |  |  |  |  |  |  | LSEGYYVMDPAK |  |
|  |  |  |  |  |  |  |  |  |  |  | GSSQIIAQYYQFIR |  |
|  |  |  |  |  |  |  |  |  |  |  | NVMENCMENTR |  |
|  |  |  |  |  |  |  |  |  |  |  | DIGVPLVAFSLK |  |
|  |  |  |  |  |  |  |  |  |  |  | HTVFEVADSLR |  |
|  |  |  |  |  |  |  |  |  |  |  | HTVFEVADSLRK |  |
|  |  |  |  |  |  |  |  |  |  |  | FGWTVPAYTMPANAEHV  AVLR |  |
|  |  |  |  |  |  |  |  |  |  |  | FGWTVPAYTMPANAEHV  AVLR Oxidation (M) |  |
|  |  |  |  |  |  |  |  |  |  |  | VVIREDFSR |  |
|  |  |  |  |  |  |  |  |  |  |  | STVHKSETEAEQEIVSR |  |
|  |  |  |  |  |  |  |  |  |  |  | SETEAEQEIVSR |  |
| 4730 | tr\|Q0GE69\|Q0GE6 | NBS-containing resistance |  | *Prunus cerasus var. caproniana* | Unclear | 11.02-  Disease/defense/Defens e-related | 52 | 46 | 18538 | 5.7 | EVYATHGLVPLQK | 6 |
|  |  |  |  |  |  |  |  |  |  |  | QLLSNILR |  |
|  |  |  |  |  |  |  |  |  |  |  | KVLLILDDVDQSDQLEMLI  R |  |
|  |  |  |  |  |  |  |  |  |  |  | DCFGLGSRIIITTR |  |
|  |  |  |  |  |  |  |  |  |  |  | VYKVMPLTQDEALYLFSR |  |
|  |  |  |  |  |  |  |  |  |  |  | VMPLTQDEALYLFSR  Oxidation (M) |  |
| 5223 | sp\|Q84V25\|ENOXE | 2-methylene-furan-  3-one reductase |  | *Fragaria*  *ananassa* | Chloroplast | 02.20-Energy/Electron-  transport | 52 | 30 | 34205 | 5.5 | AWVYSEYGK | 8 |
|  |  |  |  |  |  |  |  |  |  |  | FDPSVAVPEVK |  |
|  |  |  |  |  |  |  |  |  |  |  | FDPSVAVPEVKEDQVLIK |  |
|  |  |  |  |  |  |  |  |  |  |  | VVAASLNPVDFK |  |
|  |  |  |  |  |  |  |  |  |  |  | VVAASLNPVDFKR |  |
|  |  |  |  |  |  |  |  |  |  |  | DTDSPLPTVPGYDVAGVV  VKVGSQVTK |  |

|  |  |  |  |  |  |  |  |  |  |  | FKVGDEVYGDLNEAALVN  PTR |  |
| --- | --- | --- | --- | --- | --- | --- | --- | --- | --- | --- | --- | --- |
|  |  |  |  |  |  |  |  |  |  |  | LKPYLESGK |  |
| 5521 | tr\|A0A0F7EVJ8\|A | RuBisCO |  | *Malus domestica* | Chloroplast | 02.30-  Energy/Photosynthesis | 72 | 34 | 50013 | 6.5 | DYKLTYYTPDYETK | 15 |
|  |  |  |  |  |  |  |  |  |  |  | LTYYTPDYETK |  |
|  |  |  |  |  |  |  |  |  |  |  | LTYYTPDYETKDTDILAAF  R |  |
|  |  |  |  |  |  |  |  |  |  |  | LEDLRIPVAYVK |  |
|  |  |  |  |  |  |  |  |  |  |  | TFQGPPHGIQVER |  |
|  |  |  |  |  |  |  |  |  |  |  | YGRPLLGCTIKPK |  |
|  |  |  |  |  |  |  |  |  |  |  | AVYECLRGGLDFTK |  |
|  |  |  |  |  |  |  |  |  |  |  | GGLDFTKDDENVNSQPFM  R |  |
|  |  |  |  |  |  |  |  |  |  |  | GGLDFTKDDENVNSQPFM  R Oxidation (M) |  |
|  |  |  |  |  |  |  |  |  |  |  | DRFLFCAEAIYK |  |
|  |  |  |  |  |  |  |  |  |  |  | FLFCAEAIYK |  |
|  |  |  |  |  |  |  |  |  |  |  | ELGVPIVMHDYLTGGFTA  NTTLAHYCR |  |
|  |  |  |  |  |  |  |  |  |  |  | EITLGFVDLLR |  |
|  |  |  |  |  |  |  |  |  |  |  | EITLGFVDLLRDDFVEK |  |
|  |  |  |  |  |  |  |  |  |  |  | DLAREGNEIIR |  |
| 5627 | sp\|P28475\|S6PD_ | NADP-dependent D-sorbitol-6- phosphate  dehydrogenase |  | *Malus domestica* | Chloroplast/Cy toplasm/ Peroxisome. | 01.05-  Metabolism/Sugars and polysaccharides | 46 | 35 | 35168 | 6.9 | STVTLSSGYEMPVIGLGLW R | 8 |
|  |  |  |  |  |  |  |  |  |  |  | EVILNAIK |  |
|  |  |  |  |  |  |  |  |  |  |  | LQIDYLDLYLVHYPMPTK  Oxidation (M) |  |
|  |  |  |  |  |  |  |  |  |  |  | VLDIDVTISLQQTWEGME  K Oxidation (M) |  |
|  |  |  |  |  |  |  |  |  |  |  | TVSLGLVR |  |
|  |  |  |  |  |  |  |  |  |  |  | HGVLPTAHTPLGGAAANK |  |
|  |  |  |  |  |  |  |  |  |  |  | DMFGSVSPLDDPVLNDVA  K |  |
|  |  |  |  |  |  |  |  |  |  |  | DMFGSVSPLDDPVLNDVA  KK |  |
| 5840 | tr\|Q6YNR9\|Q6YNR | Aconitate  hydratase |  | *Prunus avium* | Cytosol | 02.10-Energy/TCA  pathway | 51 | 24 | 99444 | 6 | ILTALEKPDGGEFGK | 15 |

|  |  |  |  |  |  |  |  |  |  |  | YYSLPALNDPR |  |
| --- | --- | --- | --- | --- | --- | --- | --- | --- | --- | --- | --- | --- |
|  |  |  |  |  |  |  |  |  |  |  | ILLESAIR |  |
|  |  |  |  |  |  |  |  |  |  |  | QVEIPFKPAR |  |
|  |  |  |  |  |  |  |  |  |  |  | VPLQDFTGVPAVVDLACM  R Oxidation (M) |  |
|  |  |  |  |  |  |  |  |  |  |  | INPLVPVDLVIDHSVQVDV  AR |  |
|  |  |  |  |  |  |  |  |  |  |  | DGVTATDLVLTVTQMLR |  |
|  |  |  |  |  |  |  |  |  |  |  | DGVTATDLVLTVTQMLR  Oxidation (M) |  |
|  |  |  |  |  |  |  |  |  |  |  | FVEFYGEGMSELSLADR |  |
|  |  |  |  |  |  |  |  |  |  |  | SDDKVALIESYLR |  |
|  |  |  |  |  |  |  |  |  |  |  | VYSSYLELNLNEVEPCISG  PK |  |
|  |  |  |  |  |  |  |  |  |  |  | STYIHEPPYFK |  |
|  |  |  |  |  |  |  |  |  |  |  | NAYCLLNFGDSITTDHISP  AGSIHK |  |
|  |  |  |  |  |  |  |  |  |  |  | DFNSYGSR |  |
|  |  |  |  |  |  |  |  |  |  |  | FDTEVELAYFDHGGILQYV  IR |  |
| 5846 | tr\|K4HUT4\|K4HUT | Sucrose synthase |  | *Malus domestica* | Cytosol | 01.05-  Metabolism/Sugars and polysaccharides | 53 | 12 | 93175 | 5.7 | VNVYELSVEELTVSEYLR | 6 |
|  |  |  |  |  |  |  |  |  |  |  | SSSIGNGVQFLNR |  |
|  |  |  |  |  |  |  |  |  |  |  | LQPETPYSEFEYLFQGMGF  ER |  |
|  |  |  |  |  |  |  |  |  |  |  | DTVGQYESHSSYTLPGQY  R |  |
|  |  |  |  |  |  |  |  |  |  |  | DLANLVIVAGYIDAK |  |
|  |  |  |  |  |  |  |  |  |  |  | EDPSYWNTISDAGLQR |  |
| 6016 | gi\|658003653 | Apolipoprotein |  | *Malus domestica* | Cell membrane.  Cell wall/Cytoplasm  . Nucleus.  Vacuole. | 07.99-  Transporters/Others | 71 | 38 | 21471 | 6.2 | WYEIASVPSR | 6 |
|  |  |  |  |  |  |  |  |  |  |  | DDGTVNVLNETWTDGK |  |
|  |  |  |  |  |  |  |  |  |  |  | GFIEGTAYK |  |
|  |  |  |  |  |  |  |  |  |  |  | NSLWILSR |  |
|  |  |  |  |  |  |  |  |  |  |  | RPHLDEEIYNQLVQR |  |
|  |  |  |  |  |  |  |  |  |  |  | QSESPPEGEEGPK |  |

| 6018 | tr\|Q6V8T2\|Q6V8T | Actin- depolymerizing  factor |  | *Malus domestica* | Unclear | 09.04-Cell structure/Cytoskeleton | 74 | 64 | 11189 | 9.5 | SIVFKIEEK | 6 |
| --- | --- | --- | --- | --- | --- | --- | --- | --- | --- | --- | --- | --- |
|  |  |  |  |  |  |  |  |  |  |  | QKQVVVEHVGEPAETYEQ  FTEK |  |
|  |  |  |  |  |  |  |  |  |  |  | QVVVEHVGEPAETYEQFT  EK |  |
|  |  |  |  |  |  |  |  |  |  |  | YAIFDFDFLTPEGVQK |  |
|  |  |  |  |  |  |  |  |  |  |  | SRIFFIAWSPDTSR |  |
|  |  |  |  |  |  |  |  |  |  |  | IFFIAWSPDTSR |  |
| 6218 | gi\|657974830 | 2-methylene-furan-  3-one reductase |  | *Malus domestica* | Chloroplast | 02.20-Energy/Electron-  transport |  | 25 | 36974 | 7.6 | VVAASLNPVDFK | 6 |
|  |  |  |  |  |  |  |  |  |  |  | IGSLAEYTAAEER |  |
|  |  |  |  |  |  |  |  |  |  |  | NLSFVEAASLPLAIETAYE  GLER |  |
|  |  |  |  |  |  |  |  |  |  |  | FDVVYDAVGQSDR |  |
|  |  |  |  |  |  |  |  |  |  |  | VKPVLDPTGPYPFSK |  |
|  |  |  |  |  |  |  |  |  |  |  | TVEAFAYLETSR |  |
| 6225 | tr\|M5XFH2\|M5XFH | Fructose- bisphosphate  aldolase |  | *Prunus persica* | Cytosol | 02.01-  Energy/Glycolysis | 59 | 25 | 42973 | 9 | GILAIDESNATCGK | 8 |
|  |  |  |  |  |  |  |  |  |  |  | GILAIDESNATCGKR |  |
|  |  |  |  |  |  |  |  |  |  |  | RLDSIGLDNTEVNR |  |
|  |  |  |  |  |  |  |  |  |  |  | LDSIGLDNTEVNR |  |
|  |  |  |  |  |  |  |  |  |  |  | GLVPLPGSNNESWCQGLD  GLASR |  |
|  |  |  |  |  |  |  |  |  |  |  | TVVSIPCGPSALAVK |  |
|  |  |  |  |  |  |  |  |  |  |  | EAAWGLAR |  |
|  |  |  |  |  |  |  |  |  |  |  | YAAISQDNGLVPIVEPEILL  DGDHPIER |  |
| 6545 | gi\|657975136 | Mitochondrial- processing peptidase subunit  beta |  | *Malus domestica* | Mitochondrion | 06.04-Protein destination and storage/Targeting | 115 | 31 | 59284 | 6.3 | YGSPHPSLTDHSHILTAPET R | 13 |
|  |  |  |  |  |  |  |  |  |  |  | VTTLPNGLR |  |
|  |  |  |  |  |  |  |  |  |  |  | TATVGVWIDAGSR |  |
|  |  |  |  |  |  |  |  |  |  |  | DLEEEVENMGGHLNAYTS  R |  |
|  |  |  |  |  |  |  |  |  |  |  | DLEEEVENMGGHLNAYTS  R Oxidation (M) |  |

|  |  |  |  |  |  |  |  |  |  |  | LSGDPATASQLVAEEPSFF  TGSEVR |  |
| --- | --- | --- | --- | --- | --- | --- | --- | --- | --- | --- | --- | --- |
|  |  |  |  |  |  |  |  |  |  |  | HMGSELAQR |  |
|  |  |  |  |  |  |  |  |  |  |  | VAINDIAENYMAFNTNYK |  |
|  |  |  |  |  |  |  |  |  |  |  | DTGLFGVFATAK |  |
|  |  |  |  |  |  |  |  |  |  |  | SSLLLHLDGTSAVAEDIGR |  |
|  |  |  |  |  |  |  |  |  |  |  | QLLTYGR |  |
|  |  |  |  |  |  |  |  |  |  |  | IPLAEMCAR |  |
|  |  |  |  |  |  |  |  |  |  |  | LPDYNWFR |  |
| 6834 | tr\|B7SKM8\|B7SKM | Phosphoenolpyruv  ate carboxylase |  | *Malus domestica* | Cytosol | 02.10-Energy/TCA  pathway | 46 | 16 | 110632 | 7.4 | KSPQEVFDALK | 16 |
|  |  |  |  |  |  |  |  |  |  |  | NQTVDLVLTAHPTQSVR |  |
|  |  |  |  |  |  |  |  |  |  |  | EIQAAFR |  |
|  |  |  |  |  |  |  |  |  |  |  | HYIEFWK |  |
|  |  |  |  |  |  |  |  |  |  |  | LYHTRER |  |
|  |  |  |  |  |  |  |  |  |  |  | AIADGSLLDFLR |  |
|  |  |  |  |  |  |  |  |  |  |  | QESDRHTDVIDAITK |  |
|  |  |  |  |  |  |  |  |  |  |  | FSAAWQLYK |  |
|  |  |  |  |  |  |  |  |  |  |  | FGVKLTMFHGR Oxidation  (M) |  |
|  |  |  |  |  |  |  |  |  |  |  | LTMFHGR |  |
|  |  |  |  |  |  |  |  |  |  |  | GGGPTHLAILSQPPDTIHGS  LR |  |
|  |  |  |  |  |  |  |  |  |  |  | RALMDEMAVVSTEDYR  Oxidation (M) |  |
|  |  |  |  |  |  |  |  |  |  |  | SIVFQEPR |  |
|  |  |  |  |  |  |  |  |  |  |  | FVEYFR |  |
|  |  |  |  |  |  |  |  |  |  |  | MNIGSRPSK |  |
| 6835 | tr\|M5WQE7\|M5WQE | Translation elongation factor | 100% to Prunus persica gi\|388540218  E-value: 0.0 | *Prunus persica* | Unclear | 05.04-Protein synthesis/Translation factors | 190 | 47 | 91248 | 5.5 | KSTLTDSLVAAAGIIAQEV AGDVR | 32 |
|  |  |  |  |  |  |  |  |  |  |  | STLTDSLVAAAGIIAQEVA  GDVR |  |
|  |  |  |  |  |  |  |  |  |  |  | NGNEYLINLIDSPGHVDFS  SEVTAALR |  |
|  |  |  |  |  |  |  |  |  |  |  | ITDGALVVVDCIEGVCVQT  ETVLR |  |
|  |  |  |  |  |  |  |  |  |  |  | IRPVLTVNK |  |
|  |  |  |  |  |  |  |  |  |  |  | CFLELQVDGEEAYQTFQR |  |

|  |  |  |  |  |  |  |  |  |  |  | VIENANVIMATYEDPLLGD  VQVYPEK |  |
| --- | --- | --- | --- | --- | --- | --- | --- | --- | --- | --- | --- | --- |
|  |  |  |  |  |  |  |  |  |  |  | GTVAFSAGLHGWAFTLTN  FAK |  |
|  |  |  |  |  |  |  |  |  |  |  | LWGENYFDPATK |  |
|  |  |  |  |  |  |  |  |  |  |  | LWGENYFDPATKK |  |
|  |  |  |  |  |  |  |  |  |  |  | GFVQFCYEPIK |  |
|  |  |  |  |  |  |  |  |  |  |  | EKLWPMLTK |  |
|  |  |  |  |  |  |  |  |  |  |  | LGVTMKSDEK |  |
|  |  |  |  |  |  |  |  |  |  |  | YRVENLYEGPLDDQYANA  IR |  |
|  |  |  |  |  |  |  |  |  |  |  | VENLYEGPLDDQYANAIR |  |
|  |  |  |  |  |  |  |  |  |  |  | NCDPEGPLMLYVSK |  |
|  |  |  |  |  |  |  |  |  |  |  | VQTGLKVR |  |
|  |  |  |  |  |  |  |  |  |  |  | VRIMGPNYVPGEK  Oxidation (M) |  |
|  |  |  |  |  |  |  |  |  |  |  | FSVSPVVR |  |
|  |  |  |  |  |  |  |  |  |  |  | DLQDDFMGGAEIIK |  |
|  |  |  |  |  |  |  |  |  |  |  | LYMEARPLEEGLPEAIDDG  R |  |
|  |  |  |  |  |  |  |  |  |  |  | ILAEEFGWDK |  |
|  |  |  |  |  |  |  |  |  |  |  | ILAEEFGWDKDLAK |  |
|  |  |  |  |  |  |  |  |  |  |  | KIWCFGPETTGPNMVVDM  CK |  |
|  |  |  |  |  |  |  |  |  |  |  | IWCFGPETTGPNMVVDMC  K |  |
|  |  |  |  |  |  |  |  |  |  |  | EGALAEENMR |  |
|  |  |  |  |  |  |  |  |  |  |  | GGGQVIPTAR |  |
|  |  |  |  |  |  |  |  |  |  |  | VIYASQLTAKPR |  |
|  |  |  |  |  |  |  |  |  |  |  | RGHVFEEMQRPGTPLYNI  K Oxidation (M) |  |
|  |  |  |  |  |  |  |  |  |  |  | GHVFEEMQRPGTPLYNIK |  |
|  |  |  |  |  |  |  |  |  |  |  | GHVFEEMQRPGTPLYNIK  Oxidation (M) |  |
|  |  |  |  |  |  |  |  |  |  |  | EQMTPLSEFEDKL |  |
| 6905 | tr\|A0A0C5GYF8\|A | Phosphoenolpyruv  ate carboxylase |  | *Prunus sibirica* | Cytoplasm | 02.10-Energy/TCA  pathway | 58 | 14 | 110706 | 6.1 | ETVQDCYELSAEYER | 11 |
|  |  |  |  |  |  |  |  |  |  |  | VPYNAPLIQFSSWMGGDR |  |
|  |  |  |  |  |  |  |  |  |  |  | QIPPNEPYR |  |
|  |  |  |  |  |  |  |  |  |  |  | LFSIDWYK |  |

|  |  |  |  |  |  |  |  |  |  |  | GGGPTHLAILSQPPDTIHGS  LR |  |
| --- | --- | --- | --- | --- | --- | --- | --- | --- | --- | --- | --- | --- |
|  |  |  |  |  |  |  |  |  |  |  | VTVQGEVIEQSFGEEHLCF  R |  |
|  |  |  |  |  |  |  |  |  |  |  | SIVFQEPR |  |
|  |  |  |  |  |  |  |  |  |  |  | FVEYFR |  |
|  |  |  |  |  |  |  |  |  |  |  | EMYNQWPFFR |  |
|  |  |  |  |  |  |  |  |  |  |  | GDPGIASLYDK |  |
|  |  |  |  |  |  |  |  |  |  |  | ALLEGDPYLR |  |
| 6908 | tr\|A0A0C5GYF8\|A | Phosphoenolpyruv  ate carboxylase |  | *Prunus sibirica* | Cytoplasm | 02.10-Energy/TCA  pathway | 64 | 13 | 110706 | 6.1 | ETVQDCYELSAEYER | 12 |
|  |  |  |  |  |  |  |  |  |  |  | EIQAAFR |  |
|  |  |  |  |  |  |  |  |  |  |  | VPYNAPLIQFSSWMGGDR |  |
|  |  |  |  |  |  |  |  |  |  |  | HLEIGSYR |  |
|  |  |  |  |  |  |  |  |  |  |  | EWSEER |  |
|  |  |  |  |  |  |  |  |  |  |  | LFSIDWYK |  |
|  |  |  |  |  |  |  |  |  |  |  | VTVQGEVIEQSFGEEHLCF  R |  |
|  |  |  |  |  |  |  |  |  |  |  | SIVFQEPR |  |
|  |  |  |  |  |  |  |  |  |  |  | FVEYFR |  |
|  |  |  |  |  |  |  |  |  |  |  | DAKNLHMLR Oxidation  (M) |  |
|  |  |  |  |  |  |  |  |  |  |  | GDPGIASLYDK |  |
|  |  |  |  |  |  |  |  |  |  |  | ALLEGDPYLR |  |
| 7012 | tr\|H2EIF7\|H2EIF | ADP-ribosylation  factor |  | *Malus domestica* | Unclear | 08.07-Intracellular  traffic/Vesicular | 124 | 59 | 21566 | 6.2 | ILMVGLDAAGK | 11 |
|  |  |  |  |  |  |  |  |  |  |  | LKLGEIVTTIPTIGFNVETV  EYK |  |
|  |  |  |  |  |  |  |  |  |  |  | LGEIVTTIPTIGFNVETVEY  K |  |
|  |  |  |  |  |  |  |  |  |  |  | NISFTVWDVGGQDK |  |
|  |  |  |  |  |  |  |  |  |  |  | IRPLWR |  |
|  |  |  |  |  |  |  |  |  |  |  | HYFQNTQGLIFVVDSNDR |  |
|  |  |  |  |  |  |  |  |  |  |  | VVEARDELHR |  |
|  |  |  |  |  |  |  |  |  |  |  | MLNEDELR |  |
|  |  |  |  |  |  |  |  |  |  |  | QDLPNAMNAAEITDK |  |
|  |  |  |  |  |  |  |  |  |  |  | QDLPNAMNAAEITDKLGL  HSLR Oxidation (M) |  |
|  |  |  |  |  |  |  |  |  |  |  | LGLHSLR |  |

| 7227 | tr\|M5W2W1\|M5W2W | 3-oxoacyl-[acyl- carrier-protein] reductase | 96% to Prunus mume gi\|645270925 E-  value: 1e-180 | *Prunus persica* | Chloroplast | 01.06-Metabolism/Lipid and sterol | 61 | 23 | 31340 | 8.9 | MANQVADHLEPWR | 6 |
| --- | --- | --- | --- | --- | --- | --- | --- | --- | --- | --- | --- | --- |
|  |  |  |  |  |  |  |  |  |  |  | VVMVTGASSGLGR |  |
|  |  |  |  |  |  |  |  |  |  |  | TNLTGSWLVSK |  |
|  |  |  |  |  |  |  |  |  |  |  | VNAISPGLFR |  |
|  |  |  |  |  |  |  |  |  |  |  | DWLHNVAMK |  |
|  |  |  |  |  |  |  |  |  |  |  | TFGTSDPALTSLVR |  |
| 7711 | tr\|M5VVJ1\|M5VVJ | NADP-dependent  malic enzyme |  | *Prunus persica* | Chloroplast | 02.10-Energy/TCA  pathway | 52 | 17 | 55230 | 7.7 | QYQVPLQK | 8 |
|  |  |  |  |  |  |  |  |  |  |  | YMALTELQER |  |
|  |  |  |  |  |  |  |  |  |  |  | RPQGLYISLK |  |
|  |  |  |  |  |  |  |  |  |  |  | TVQVIVVTDGER |  |
|  |  |  |  |  |  |  |  |  |  |  | ILGLGDLGCQGMGIPVGK |  |
|  |  |  |  |  |  |  |  |  |  |  | IWLVDSK |  |
|  |  |  |  |  |  |  |  |  |  |  | AIKPTVLIGSSGVGR |  |
|  |  |  |  |  |  |  |  |  |  |  | LPRPENLVK |  |
| 7720 | tr\|M5WFB9\|M5WFB | 5-  methyltetrahydropt eroyltriglutamate- homocysteine  methyltransferase | 100% to Prunus mume gi\|645263470  E-value: 0.0 | *Prunus persica* | Chloroplast | 01.01-  Metabolism/Amino Acid | 69 | 18 | 84901 | 6.1 | YGWNGGEIGFDTYFSMAR | 13 |
|  |  |  | 98% to Prunus mume gi\|645263470  E-value: 0.0 |  |  |  |  |  |  |  | YGWNGGEIGFDTYFSMAR  Oxidation (M) |  |
|  |  |  |  |  |  |  |  |  |  |  | TLTSLKGVTAYGFDLVR |  |
|  |  |  |  |  |  |  |  |  |  |  | GVTAYGFDLVR |  |
|  |  |  |  |  |  |  |  |  |  |  | SWLAFAAQK |  |
|  |  |  |  |  |  |  |  |  |  |  | ALAGHKDEAFFSANAAAQ  ASR |  |
|  |  |  |  |  |  |  |  |  |  |  | DEAFFSANAAAQASR |  |
|  |  |  |  |  |  |  |  |  |  |  | AAAALKGSDHR |  |
|  |  |  |  |  |  |  |  |  |  |  | KLNLPILPTTTIGSFPQTIEL  R |  |
|  |  |  |  |  |  |  |  |  |  |  | LNLPILPTTTIGSFPQTIELR |  |
|  |  |  |  |  |  |  |  |  |  |  | LQEELDIDVLVHGEPER |  |
|  |  |  |  |  |  |  |  |  |  |  | SDEKLLSVFR |  |
|  |  |  |  |  |  |  |  |  |  |  | YGAGIGPGVYDIHSPR |  |

| 7728 | tr\|M5WFB9\|M5WFB | 5-  methyltetrahydropt eroyltriglutamate-- homocysteine  methyltransferase | 100% to Prunus mume gi\|645263470  E-value: 0.0 | *Prunus persica* | Chloroplast | 01.01-  Metabolism/Amino Acid | 52 | 16 | 84901 | 6.1 | YGWNGGEIGFDTYFSMAR | 9 |
| --- | --- | --- | --- | --- | --- | --- | --- | --- | --- | --- | --- | --- |
|  |  |  |  |  |  |  |  |  |  |  | GVTAYGFDLVR |  |
|  |  |  |  |  |  |  |  |  |  |  | YLFAGLVDGR |  |
|  |  |  |  |  |  |  |  |  |  |  | ALAGHKDEAFFSANAAAQ  ASR |  |
|  |  |  |  |  |  |  |  |  |  |  | DEAFFSANAAAQASR |  |
|  |  |  |  |  |  |  |  |  |  |  | AAAALKGSDHR |  |
|  |  |  |  |  |  |  |  |  |  |  | LNLPILPTTTIGSFPQTIELR |  |
|  |  |  |  |  |  |  |  |  |  |  | LQEELDIDVLVHGEPER |  |
|  |  |  |  |  |  |  |  |  |  |  | YGAGIGPGVYDIHSPR |  |
| 8115 | tr\|C0IRH7\|C0IRH | Xyloglucan endotransglucosyla  se/hydrolase |  | *Malus domestica* | Cell wall | 09.01-Cell structure/Cell wall | 140 | 49 | 34142 | 7.8 | VTWSDSHIR | 18 |
|  |  |  |  |  |  |  |  |  |  |  | AIQLVLDQNSGCGFASK |  |
|  |  |  |  |  |  |  |  |  |  |  | QKYLFGR |  |
|  |  |  |  |  |  |  |  |  |  |  | YLFGRVSMK Oxidation (M) |  |
|  |  |  |  |  |  |  |  |  |  |  | LIPGDSAGTVTAFYMNSDT  NTVR |  |
|  |  |  |  |  |  |  |  |  |  |  | LIPGDSAGTVTAFYMNSDT  NTVR Oxidation (M) |  |
|  |  |  |  |  |  |  |  |  |  |  | TGQPYTVQTNIYAHGK |  |
|  |  |  |  |  |  |  |  |  |  |  | TGQPYTVQTNIYAHGKGD  R |  |
|  |  |  |  |  |  |  |  |  |  |  | GDREQR |  |
|  |  |  |  |  |  |  |  |  |  |  | LQPMGVFSTLWEADDWA  TR |  |
|  |  |  |  |  |  |  |  |  |  |  | LQPMGVFSTLWEADDWA  TR Oxidation (M) |  |
|  |  |  |  |  |  |  |  |  |  |  | LQPMGVFSTLWEADDWA  TRGGLEK |  |
|  |  |  |  |  |  |  |  |  |  |  | APFFSYYK |  |
|  |  |  |  |  |  |  |  |  |  |  | YRWVR |  |

|  |  |  |  |  |  |  |  |  |  |  | INHMIYDYCTDR |  |
| --- | --- | --- | --- | --- | --- | --- | --- | --- | --- | --- | --- | --- |
|  |  |  |  |  |  |  |  |  |  |  | INHMIYDYCTDR Oxidation  (M) |  |
|  |  |  |  |  |  |  |  |  |  |  | SRYPVAPPECTAGF |  |
|  |  |  |  |  |  |  |  |  |  |  | YPVAPPECTAGF |  |
| 8116 | tr\|Q154Z0\|Q154Z | Putative NBS-LRR  disease resistance protein |  | *Malus domestica* | Unclear | 11.02-  Disease/defense/Defens e-related | 43 | 31 | 16498 | 5.7 | IFHHFEVHCFLDSPMLK | 3 |
|  |  |  |  |  |  |  |  |  |  |  | ILTQQWGINYTK |  |
|  |  |  |  |  |  |  |  |  |  |  | NMPEDGFLELSECFIK |  |
| 8232 | sp\|P28475\|S6PD_ | NADP-dependent D-sorbitol-6- phosphate  dehydrogenase |  | *Malus domestica* | Chloroplast | 01.05-  Metabolism/Sugars and polysaccharides | 130 | 60 | 35168 | 6.9 | HFDCAAHYK | 15 |
|  |  |  |  |  |  |  |  |  |  |  | SEADVGEALAEAFK |  |
|  |  |  |  |  |  |  |  |  |  |  | REELFITTK |  |
|  |  |  |  |  |  |  |  |  |  |  | IWNSDHGHVVEACK |  |
|  |  |  |  |  |  |  |  |  |  |  | VLDIDVTISLQQTWEGME  K Oxidation (M) |  |
|  |  |  |  |  |  |  |  |  |  |  | TVSLGLVR |  |
|  |  |  |  |  |  |  |  |  |  |  | SIGLSNYELFLTR |  |
|  |  |  |  |  |  |  |  |  |  |  | IKPAVSQFETHPYFQR |  |
|  |  |  |  |  |  |  |  |  |  |  | HGVLPTAHTPLGGAAANK |  |
|  |  |  |  |  |  |  |  |  |  |  | DMFGSVSPLDDPVLNDVA  K |  |
|  |  |  |  |  |  |  |  |  |  |  | DMFGSVSPLDDPVLNDVA  K Oxidation (M) |  |
|  |  |  |  |  |  |  |  |  |  |  | SVAQICLR |  |
|  |  |  |  |  |  |  |  |  |  |  | SVAQICLRWGIQR |  |
|  |  |  |  |  |  |  |  |  |  |  | ENLEVLEFQLSDEDMQLIY  SIDR |  |
|  |  |  |  |  |  |  |  |  |  |  | TSLPSKTWGLDVYA |  |
| 8319 | tr\|C0LQA0\|C0LQA | GDP-D-mannose pyrophosphorylase |  | *Malus domestica* | Chloroplast | 01.05-  Metabolism/Sugars and polysaccharides | 43 | 22 | 36849 | 5.9 | ITCSQETEPLGTAGPLALA R | 4 |
|  |  |  |  |  |  |  |  |  |  |  | LIDDSGEPFFVLNSDVISEY  PFK |  |
|  |  |  |  |  |  |  |  |  |  |  | LYLDSLR |  |
|  |  |  |  |  |  |  |  |  |  |  | GEGCLIGPDVAIGPGCIIES  GVR |  |

| 8530 | gi\|658010328 | Pyruvate kinase  isozyme |  | *Malus domestica* | Cytosol | 02.01-  Energy/Glycolysis | 57 | 16 | 55983 | 9 | NTILCADGTITLTVLSCDP  AAGTVR | 6 |
| --- | --- | --- | --- | --- | --- | --- | --- | --- | --- | --- | --- | --- |
|  |  |  |  |  |  |  |  |  |  |  | IDMIALSFVR |  |
|  |  |  |  |  |  |  |  |  |  |  | IDMIALSFVR Oxidation (M) |  |
|  |  |  |  |  |  |  |  |  |  |  | VENQEGVINFDEILR |  |
|  |  |  |  |  |  |  |  |  |  |  | STPLPMSPLESLASSAVR |  |
|  |  |  |  |  |  |  |  |  |  |  | LCTPGDAVVALHR |  |
| 8606 | tr\|M5XNZ7\|M5XNZ | ATP-citrate synthase beta chain protein | 99% to Prunus mume gi\|645233471  Evalue: 0.0 | *Prunus persica* | Cytosol | 01.06-Metabolism/Lipid and sterol | 52 | 20 | 66383 | 8.8 | MLDFDFLCGR | 9 |
|  |  |  |  |  |  |  |  |  |  |  | LYRPGSVGFVSK |  |
|  |  |  |  |  |  |  |  |  |  |  | SGGMSNELYNTVAR |  |
|  |  |  |  |  |  |  |  |  |  |  | VTDGLFEGIAIGGDVFPGS  TLSDHVLR |  |
|  |  |  |  |  |  |  |  |  |  |  | LIVVLGELGGR |  |
|  |  |  |  |  |  |  |  |  |  |  | ETFEKLVEQGK |  |
|  |  |  |  |  |  |  |  |  |  |  | EFKPPQIPEDLNSAIK |  |
|  |  |  |  |  |  |  |  |  |  |  | NLTPYEFVESMK |  |
|  |  |  |  |  |  |  |  |  |  |  | VELLQAFAR |  |
| 8608 | tr\|F2YQ40\|F2YQ4 | Pyrophosphate- fructose 6-  phosphate 1- phosphotransferase | 99% to Prunus mume gi\|1027110833  E-value: 0.0 | *Prunus mume* | Chloroplast/ Cytoplasm | 01.05-  Metabolism/Sugars and polysaccharides | 113 | 34 | 68113 | 7.2 | SQYQPELPPCLQGATVR | 16 |
|  |  |  |  |  |  |  |  |  |  |  | VEFGDTTTSLDPTDSHTISR |  |
|  |  |  |  |  |  |  |  |  |  |  | YFPHTYGQPLAHFLR |  |
|  |  |  |  |  |  |  |  |  |  |  | VPGAQVITEHPPVR |  |
|  |  |  |  |  |  |  |  |  |  |  | VHNPNNTLLGFLGGSEGLF  AQK |  |
|  |  |  |  |  |  |  |  |  |  |  | TLEITDEILATYK |  |
|  |  |  |  |  |  |  |  |  |  |  | NQGGYDLLGR |  |
|  |  |  |  |  |  |  |  |  |  |  | TVEQVNATLTACK |  |
|  |  |  |  |  |  |  |  |  |  |  | NQFVEANVGFDTICK |  |
|  |  |  |  |  |  |  |  |  |  |  | YYYFIR |  |
|  |  |  |  |  |  |  |  |  |  |  | LTLFDITTQICDAVEAR |  |
|  |  |  |  |  |  |  |  |  |  |  | FNAICHFFGYQAR |  |
|  |  |  |  |  |  |  |  |  |  |  | FLLDDIYR |  |
|  |  |  |  |  |  |  |  |  |  |  | AVSLCVEDQDYMGR |  |

|  |  |  |  |  |  |  |  |  |  |  | AVSLCVEDQDYMGR  Oxidation (M) |  |
| --- | --- | --- | --- | --- | --- | --- | --- | --- | --- | --- | --- | --- |
|  |  |  |  |  |  |  |  |  |  |  | TIVKPGCSQEVLK |  |
| 9117 | gi\|658038306 | Porin |  | *Malus domestica* | Mitochondrion | 11.02-  Disease/defense/Defens e-related | 86 | 35 | 29460 | 9.2 | FTVSTYSDAGVALTSTAV K | 6 |
|  |  |  |  |  |  |  |  |  |  |  | GGLSTGDILTQYK |  |
|  |  |  |  |  |  |  |  |  |  |  | YTAGISITKPDQSASIILGD  K |  |
|  |  |  |  |  |  |  |  |  |  |  | ASYVHYMDLLK |  |
|  |  |  |  |  |  |  |  |  |  |  | FSTNENTFTVGGQYAVDIY  TIVK |  |
|  |  |  |  |  |  |  |  |  |  |  | LGALLQHEVIPK |  |
| 9118 | gi\|658038306 | Porin |  | *Malus domestica* | Mitochondrion | 11.02-  Disease/defense/Defens e-related | 76 | 34 | 29460 | 9.2 | FTVSTYSDAGVALTSTAV K | 5 |
|  |  |  |  |  |  |  |  |  |  |  | VDTESNISTTFTINEIVPSTK |  |
|  |  |  |  |  |  |  |  |  |  |  | YTAGISITKPDQSASIILGD  K |  |
|  |  |  |  |  |  |  |  |  |  |  | FSTNENTFTVGGQYAVDIY  TIVK |  |
|  |  |  |  |  |  |  |  |  |  |  | LGALLQHEVIPK |  |
| 9221 | sp\|P28475\|S6PD_ | NADP-dependent D-sorbitol-6- phosphate  dehydrogenase |  | *Malus domestica* | Chloroplast/Cy toplasm/ Peroxisome | 01.05-  Metabolism/Sugars and polysaccharides | 45 | 25 | 35168 | 6.9 | HFDCAAHYK | 7 |
|  |  |  |  |  |  |  |  |  |  |  | VLDIDVTISLQQTWEGME  KTVSLGLVR |  |
|  |  |  |  |  |  |  |  |  |  |  | TVSLGLVR |  |
|  |  |  |  |  |  |  |  |  |  |  | IKPAVSQFETHPYFQR |  |
|  |  |  |  |  |  |  |  |  |  |  | HGVLPTAHTPLGGAAANK |  |
|  |  |  |  |  |  |  |  |  |  |  | SVAQICLR |  |
|  |  |  |  |  |  |  |  |  |  |  | GKYADELIANAAYIGTPGK |  |
| 9227 | tr\|M5W2H9\|M5W2H | Fructose-  bisphosphate aldolase |  | *Prunus persica* | Mitochondrion | 02.01-  Energy/Glycolysis | 63 | 30 | 38617 | 7.6 | FASINVENVESNR | 9 |
|  |  |  |  |  |  |  |  |  |  |  | FASINVENVESNRR |  |
|  |  |  |  |  |  |  |  |  |  |  | VDKGTVELAGTNGETTTQ  GLDGLAQR |  |

|  |  |  |  |  |  |  |  |  |  |  | GTVELAGTNGETTTQGLD  GLAQR |  |
| --- | --- | --- | --- | --- | --- | --- | --- | --- | --- | --- | --- | --- |
|  |  |  |  |  |  |  |  |  |  |  | GTVELAGTNGETTTQGLD  GLAQRCQK |  |
|  |  |  |  |  |  |  |  |  |  |  | YYEAGAR |  |
|  |  |  |  |  |  |  |  |  |  |  | YAIICQENGLVPIVEPEILV  DGPHDIEK |  |
|  |  |  |  |  |  |  |  |  |  |  | KPWSLSFSFGR |  |
| 9313 | tr\|X5D281\|X5D28 | Glyceraldehyde-3- phosphate  dehydrogenase |  | *Malus baccata* | Cytoplasm/Mit ochondrion | 11.05-  Disease/Defense/Stress responses | 59 | 24 | 37104 | 9.2 | TLLFGEKPVTVFGIR | 5 |
|  |  |  |  |  |  |  |  |  |  |  | FGIVEGLMTTVHSITATQK  Oxidation (M) |  |
|  |  |  |  |  |  |  |  |  |  |  | VPTVDVSVVDLTVR |  |
|  |  |  |  |  |  |  |  |  |  |  | GILGYTEDDVVSTDFIGDS  R |  |
|  |  |  |  |  |  |  |  |  |  |  | LVSWYDNEWGYSTR |  |
| 9314 | tr\|X5D281\|X5D28 | Glyceraldehyde-3- phosphate  dehydrogenase |  | *Malus baccata* | Cytosol | 11.05-  Disease/Defense/Stress responses | 112 | 59 | 37104 | 9.2 | DDVELVAVNDPFITTDYM TYMFK | 16 |
|  |  |  |  |  |  |  |  |  |  |  | TLLFGEKPVTVFGIR |  |
|  |  |  |  |  |  |  |  |  |  |  | DAPMFVVGVNEK |  |
|  |  |  |  |  |  |  |  |  |  |  | EYKPDIHILSNASCTTNCL  APLAK |  |
|  |  |  |  |  |  |  |  |  |  |  | FGIVEGLMTTVHSITATQK |  |
|  |  |  |  |  |  |  |  |  |  |  | FGIVEGLMTTVHSITATQK  Oxidation (M) |  |
|  |  |  |  |  |  |  |  |  |  |  | TVDGPSQKDWR |  |
|  |  |  |  |  |  |  |  |  |  |  | AASFNIIPSSTGAAK |  |
|  |  |  |  |  |  |  |  |  |  |  | VLPALNGK |  |
|  |  |  |  |  |  |  |  |  |  |  | LTGMAFR |  |
|  |  |  |  |  |  |  |  |  |  |  | LTGMAFRVPTVDVSVVDL  TVR |  |
|  |  |  |  |  |  |  |  |  |  |  | VPTVDVSVVDLTVR |  |
|  |  |  |  |  |  |  |  |  |  |  | LAKPASYDQIK |  |
|  |  |  |  |  |  |  |  |  |  |  | GILGYTEDDVVSTDFIGDS  R |  |
|  |  |  |  |  |  |  |  |  |  |  | AGIALNDTFVK |  |
|  |  |  |  |  |  |  |  |  |  |  | LVSWYDNEWGYSTR |  |

**^a^** Spot N**^o^**, protein spot number of the reference gel maps presented in **Figure S1**;

**^b^** Accession number, UNIPROT accession number;

**^c^** Suggested name, Suggested protein name according to database identification or manual blast against current databases;

**^d^** Matching criteria adopted criteria for the valuation of similarity for proteins identified after manual blast against current databases. Blank cells correspond to proteins readily identified without further blast;

**^e^** Organism, organism in which the protein has been matched to by BLAST search;

**^f^** Subcellular localization, Subcellular localization of proteins was assigned based on database searches;

**^g^** Functional category, proteins ontologically classified into functional categories proposed by Bevan et al., 1998;

| **Treatments^a^** | | | | | | | | | |
| --- | --- | --- | --- | --- | --- | --- | --- | --- | --- |
| **^b^Functional classification** | Control vs. Control-Bc  (n=50) | | | Control vs. 1-MCP-Bc  (n=49) | | | Control vs. O_3_-Bc  (n=40) | | |
|  | **^c^Modulation (%)** | | | | | | | | |
|  | **Total** | **Up** | **Down** | **Total** | **Up** | **Down** | **Total** | **Up** | **Down** |
| Metabolism | 30 | 38 | 19 | 22 | 30 | 15 | 30 | 35 | 24 |
| Energy | 10 | 10 | 10 | 16 | 17 | 15 | 10 | 9 | 12 |
| Cell growth/division | 0 | 0 | 0 | 2 | 4 | 0 | 0 | 0 | 0 |
| Transcription | 0 | 0 | 0 | 2 | 0 | 4 | 3 | 0 | 6 |
| Protein synthesis | 0 | 0 | 0 | 4 | 9 | 0 | 0 | 0 | 0 |
| Protein destination and storage | 4 | 0 | 10 | 20 | 17 | 23 | 13 | 4 | 24 |
| Transporters | 6 | 7 | 5 | 4 | 4 | 4 | 5 | 0 | 12 |
| Intracellular traffic | 0 | 0 | 0 | 0 | 0 | 0 | 0 | 0 | 0 |
| Cell structure | 6 | 7 | 5 | 4 | 4 | 4 | 0 | 0 | 0 |
| Signal transduction | 4 | 3 | 5 | 0 | 0 | 0 | 8 | 9 | 6 |
| Disease/defense | 30 | 31 | 29 | 16 | 9 | 23 | 25 | 39 | 6 |
| Unclear classification | 8 | 3 | 14 | 2 | 4 | 0 | 5 | 4 | 6 |
| Secondary metabolism | 2 | 0 | 5 | 6 | 0 | 12 | 3 | 0 | 6 |

**Table S2**. Percentage (%) of modulated proteins by 1-MCP and O_3_ treatments in fruit artificially inoculated with *Botrytis cinerea* based on their functional category*.*

**^a^** Control: untreated and non-inoculated fruit, Control-Bc: Untreated fruit, inoculated with *B. cinerea,* 1-MCP-Bc: fruit treated with 1-MCP and inoculated with *B. cinerea,* O_3_-Bc: fruit incubated in O_3_ enriched-atmosphere chamber and inoculated with *B. cinerea*

**^b^** Functional category, proteins ontologically classified into functional categories proposed by Bevan et al. (1998).

**^c^** Percentage of total, up- or down-regulated proteins to each functional category

**Table S3**. Quantitative data for apple protein spot volumes on 2DE-gels. Protein extracts have been analyzed by two-dimensional electrophoresis and 2D-gels were submitted to image analysis as described in experimental procedures

| Spot N^o^ | Control Mean | Control CV (%) | Control-Bc Mean | Control-Bc CV (%) | Ratio Control Mean/Control-Bc Mean | p-value Control/ Control- Bc | 1-MCP-Bc  Mean | 1-MCP- Bc CV (%) | Ratio  1-MCP-Bc  Mean/Control Mean | p- value 1- MCP-  Bc/Contro l | O_3_-Bc  Mean | O_3_-Bc CV (%) | Ratio O_3_-Bc Mean/Control Mean | p-value O_3_-Bc  /Control | 1-MCP  Mean | 1-MCP CV (%) | Ratio 1-MCP Mean/Control Mean | p-value 1- MCP/Con trol | O_3_ Mean | O_3_ CV (%) | Ratio O_3_ Mean/Control Mean | p-value O_3_/Contro l | Ratio O_3_ Mean/1-MCP Mean | p-value O_3_/1- MCP |
| --- | --- | --- | --- | --- | --- | --- | --- | --- | --- | --- | --- | --- | --- | --- | --- | --- | --- | --- | --- | --- | --- | --- | --- | --- |
| 23 | 1233.1 | 23.60% | 573 | 16.20% | 0.465 | 0.02 | 966.2 | 18.90% | 0.784 | 0.25 | 1398.4 | 14.80% | 1.13 | 0.47 | 725 | 18.90% | 0.59 | 0.052 | 594.8 | 35.60% | 0.48 | 0.037 | 0.82 | 0.422 |
| 50 | 3382.3 | 33.90% | 1124.9 | 31.40% | 0.333 | 0.031 | 2236.1 | 15.60% | 0.661 | 0.17 | 2909.4 | 13.90% | 0.86 | 0.54 | 2501.6 | 7.70% | 0.74 | 0.26 | 1467.8 | 21.10% | 0.43 | 0.049 | 0.59 | 0.008 |
| 109 | 276.5 | 26.40% | 977.4 | 30.90% | 3.535 | 0.017 | 376.3 | 28.40% | 1.361 | 0.25 | 1189.8 | 10.00% | 4.30 | 0.00 | 376.9 | 8.80% | 1.36 | 0.095 | 529.3 | 31.00% | 1.91 | 0.071 | 1.40 | 0.19 |
| 205 | 704.6 | 8.00% | 293.3 | 26.00% | 0.416 | 0.002 | 303.1 | 21.30% | 0.430 | 0.00 | 325.5 | 12.60% | 0.46 | 0.00 | 368.4 | 17.60% | 0.52 | 0.002 | 249.9 | 63.30% | 0.35 | 0.009 | 0.68 | 0.296 |
| 313 | 1030.8 | 1.20% | 733.3 | 46.30% | 0.711 | 0.204 | 1583.2 | 22.80% | 1.536 | 0.06 | 1050.2 | 18.70% | 1.02 | 0.87 | 1184.4 | 12.90% | 1.15 | 0.157 | 661 | 47.80% | 0.64 | 0.113 | 0.56 | 0.061 |
| 317 | 823.3 | 9.00% | 351.8 | 15.50% | 0.427 | 0.001 | 438.9 | 28.30% | 0.533 | 0.01 | 449.3 | 7.10% | 0.55 | 0.00 | 512.6 | 14.20% | 0.62 | 0.007 | 357.2 | 33.50% | 0.43 | 0.005 | 0.70 | 0.127 |
| 625 | 1895.5 | 15.60% | 1956.8 | 10.30% | 1.032 | 0.782 | 1949.8 | 9.20% | 1.029 | 0.80 | 2004.6 | 0.40% | 1.06 | 0.56 | 2631.3 | 6.30% | 1.39 | 0.02 | 4341.2 | 3.90% | 2.29 | 0 | 1.65 | 0 |
| 923 | 240.1 | 17.40% | 185.2 | 14.90% | 0.771 | 0.131 | 94.3 | 26.50% | 0.393 | 0.01 | 263 | 15.60% | 1.10 | 0.54 | 547.1 | 116.60% | 2.28 | 0.452 | 276.4 | 7.00% | 1.15 | 0.244 | 0.51 | 0.503 |
| 1026 | 1540.8 | 21.90% | 1279.1 | 28.70% | 0.830 | 0.416 | 1221.1 | 20.50% | 0.793 | 0.26 | 1438.7 | 1.90% | 0.93 | 0.63 | 521.7 | 9.10% | 0.34 | 0.007 | 143.1 | 14.30% | 0.09 | 0.002 | 0.27 | 0 |
| 1034 | 665.6 | 5.70% | 1162.9 | 4.60% | 1.747 | 0 | 1047.4 | 25.30% | 1.574 | 0.07 | 1707.7 | 20.50% | 2.57 | 0.01 | 567.9 | 13.10% | 0.85 | 0.112 | 1151.3 | 66.90% | 1.73 | 0.337 | 2.03 | 0.262 |
| 1037 | 536.7 | 11.70% | 2422.3 | 13.80% | 4.513 | 0.001 | 790.2 | 34.70% | 1.472 | 0.19 | 3244.2 | 12.10% | 6.04 | 0.00 | 696.4 | 18.50% | 1.30 | 0.126 | 1559.9 | 36.60% | 2.91 | 0.037 | 2.24 | 0.063 |
| 1038 | 510.8 | 17.40% | 1131.5 | 20.10% | 2.215 | 0.012 | 544.7 | 57.90% | 1.066 | 0.87 | 1751 | 16.90% | 3.43 | 0.00 | 221.3 | 67.40% | 0.43 | 0.045 | 973.7 | 14.10% | 1.91 | 0.008 | 4.40 | 0.003 |
| 1039 | 1550.5 | 3.70% | 808.4 | 16.10% | 0.521 | 0.001 | 445.4 | 53.00% | 0.287 | 0.00 | 847.7 | 9.70% | 0.55 | 0.00 | 709.9 | 28.60% | 0.46 | 0.002 | 785 | 29.30% | 0.51 | 0.005 | 1.11 | 0.694 |
| 1130 | 2276.6 | 1.30% | 1350 | 17.80% | 0.593 | 0.003 | 752.4 | 29.70% | 0.330 | 0.00 | 1541.2 | 13.60% | 0.68 | 0.00 | 1158.3 | 17.00% | 0.51 | 0.001 | 662.4 | 43.10% | 0.29 | 0.001 | 0.57 | 0.069 |
| 1132 | 1258.5 | 7.20% | 645.9 | 19.50% | 0.513 | 0.002 | 1306.9 | 17.70% | 1.038 | 0.75 | 1013.6 | 9.90% | 0.81 | 0.03 | 1244.4 | 6.00% | 0.99 | 0.844 | 499.9 | 14.80% | 0.40 | 0 | 0.40 | 0 |
| 1212 | 1745 | 19.60% | 4360.4 | 10.30% | 2.499 | 0.001 | 1670.5 | 17.10% | 0.957 | 0.79 | 3710 | 3.20% | 2.13 | 0.00 | 1129.2 | 10.10% | 0.65 | 0.041 | 3543.7 | 55.10% | 2.03 | 0.191 | 3.14 | 0.099 |
| 1220 | 1204.4 | 3.70% | 1349.3 | 11.50% | 1.120 | 0.196 | 648.6 | 13.80% | 0.539 | 0.00 | 1129.7 | 12.90% | 0.94 | 0.44 | 799.3 | 25.20% | 0.66 | 0.027 | 715.3 | 55.50% | 0.59 | 0.101 | 0.89 | 0.76 |
| 1507 | 2080.1 | 8.50% | 1023.8 | 7.20% | 0.492 | 0.001 | 1738.3 | 12.00% | 0.836 | 0.10 | 1965.2 | 5.90% | 0.94 | 0.40 | 1845.4 | 3.50% | 0.89 | 0.097 | 1890.5 | 13.10% | 0.91 | 0.341 | 1.02 | 0.775 |
| 1510 | 967.7 | 16.30% | 1292.5 | 43.40% | 1.336 | 0.389 | 1703 | 16.20% | 1.760 | 0.02 | 718.5 | 7.70% | 0.74 | 0.06 | 957.2 | 80.80% | 0.99 | 0.983 | 541.9 | 32.20% | 0.56 | 0.035 | 0.57 | 0.416 |
| 1511 | 1189.8 | 13.10% | 920.7 | 24.20% | 0.774 | 0.162 | 826.5 | 13.50% | 0.695 | 0.03 | 520.9 | 11.60% | 0.44 | 0.00 | 645.5 | 8.60% | 0.54 | 0.005 | 385.3 | 24.00% | 0.32 | 0.002 | 0.60 | 0.014 |
| 1624 | 1827.7 | 24.10% | 646.6 | 14.30% | 0.354 | 0.01 | 1401.8 | 3.50% | 0.767 | 0.17 | 654 | 4.60% | 0.36 | 0.01 | 1009.4 | 7.90% | 0.55 | 0.034 | 1137.9 | 8.00% | 0.62 | 0.056 | 1.13 | 0.141 |
| 1725 | 907.1 | 16.20% | 563.1 | 18.50% | 0.621 | 0.03 | 764.7 | 9.60% | 0.843 | 0.21 | 590.6 | 23.30% | 0.65 | 0.05 | 984.3 | 9.20% | 1.09 | 0.483 | 1277.3 | 19.70% | 1.41 | 0.092 | 1.30 | 0.13 |
| 2029 | 533.4 | 19.90% | 2619.6 | 14.20% | 4.911 | 0.001 | 665.3 | 40.80% | 1.247 | 0.48 | 3644.6 | 15.40% | 6.83 | 0.00 | 197.6 | 27.00% | 0.37 | 0.008 | 2789.6 | 10.00% | 5.23 | 0 | 14.12 | 0 |
| 2032 | 2565.2 | 6.20% | 2240.3 | 20.40% | 0.873 | 0.309 | 3032.7 | 23.40% | 1.182 | 0.33 | 4055.4 | 19.00% | 1.58 | 0.03 | 1510.5 | 12.30% | 0.59 | 0.002 | 1200.8 | 33.40% | 0.47 | 0.005 | 0.79 | 0.292 |
| 2110 | 1355.5 | 12.50% | 478.2 | 17.90% | 0.353 | 0.001 | 929.5 | 7.40% | 0.686 | 0.02 | 720.1 | 6.40% | 0.53 | 0.00 | 1038.1 | 15.50% | 0.77 | 0.078 | 636.5 | 47.70% | 0.47 | 0.023 | 0.61 | 0.113 |
| 2113 | 823.2 | 4.70% | 411.1 | 9.20% | 0.499 | 0 | 658.5 | 23.00% | 0.800 | 0.14 | 363.6 | 11.70% | 0.44 | 0.00 | 685.2 | 22.80% | 0.83 | 0.211 | 318.1 | 62.30% | 0.39 | 0.012 | 0.46 | 0.065 |
| 2115 | 3563.3 | 3.40% | 1576.2 | 6.20% | 0.442 | 0 | 2542.4 | 18.60% | 0.713 | 0.02 | 2632.6 | 12.40% | 0.74 | 0.01 | 4462.7 | 9.70% | 1.25 | 0.026 | 2199.3 | 13.50% | 0.62 | 0.002 | 0.49 | 0.002 |
| 2213 | 6234 | 8.40% | 11211.6 | 1.80% | 1.798 | 0 | 5544 | 6.60% | 0.889 | 0.13 | 8510.1 | 7.70% | 1.37 | 0.01 | 5906.7 | 10.70% | 0.95 | 0.528 | 7733.3 | 11.90% | 1.24 | 0.07 | 1.31 | 0.047 |
| 2320 | 1350.7 | 2.00% | 4056 | 20.60% | 3.003 | 0.005 | 1792.2 | 23.00% | 1.327 | 0.14 | 1648 | 2.30% | 1.22 | 0.00 | 1284.8 | 15.90% | 0.95 | 0.609 | 1444.4 | 18.90% | 1.07 | 0.585 | 1.12 | 0.462 |
| 2326 | 668.5 | 15.40% | 509.6 | 11.00% | 0.762 | 0.079 | 599.9 | 2.30% | 0.897 | 0.32 | 792.1 | 15.40% | 1.18 | 0.25 | 347.2 | 2.20% | 0.52 | 0.006 | 298.2 | 9.20% | 0.45 | 0.004 | 0.86 | 0.041 |
| 2631 | 1178 | 17.70% | 727.3 | 9.10% | 0.617 | 0.023 | 516.2 | 4.20% | 0.438 | 0.01 | 864.3 | 12.30% | 0.73 | 0.08 | 292.8 | 16.10% | 0.25 | 0.002 | 422.8 | 11.50% | 0.36 | 0.004 | 1.44 | 0.029 |
| 2634 | 1357.5 | 1.70% | 1089.2 | 37.80% | 0.802 | 0.323 | 633.5 | 2.90% | 0.467 | 0.00 | 486.5 | 2.30% | 0.36 | 0.00 | 535.1 | 8.60% | 0.39 | 0 | 648.4 | 3.90% | 0.48 | 0 | 1.21 | 0.02 |
| 2636 | 1317.7 | 20.60% | 809.2 | 7.80% | 0.614 | 0.034 | 644.3 | 1.50% | 0.489 | 0.01 | 784.4 | 0.80% | 0.60 | 0.03 | 527.9 | 1.40% | 0.40 | 0.007 | 757.8 | 18.90% | 0.58 | 0.034 | 1.44 | 0.05 |
| 2727 | 756 | 20.40% | 617.4 | 5.30% | 0.817 | 0.202 | 374.1 | 10.70% | 0.495 | 0.01 | 234.4 | 1.90% | 0.31 | 0.00 | 830.4 | 7.80% | 1.10 | 0.484 | 726.7 | 6.70% | 0.96 | 0.769 | 0.88 | 0.091 |
| 2839 | 225.7 | 9.50% | 325.9 | 5.50% | 1.444 | 0.003 | 496.6 | 14.00% | 2.200 | 0.00 | 306.7 | 6.50% | 1.36 | 0.01 | 562.8 | 4.20% | 2.49 | 0 | 670.3 | 6.40% | 2.97 | 0 | 1.19 | 0.019 |
| 3040 | 1324.9 | 20.30% | 1941.1 | 17.30% | 1.465 | 0.068 | 2384.3 | 16.60% | 1.800 | 0.02 | 3017.8 | 39.00% | 2.28 | 0.07 | 572.5 | 11.60% | 0.43 | 0.009 | 651.9 | 65.50% | 0.49 | 0.082 | 1.14 | 0.766 |
| 3211 | 109.9 | 10.70% | 267.3 | 9.30% | 2.432 | 0.001 | 129 | 14.80% | 1.174 | 0.21 | 219.4 | 9.80% | 2.00 | 0.00 | 93.6 | 15.90% | 0.85 | 0.21 | 155.3 | 17.00% | 1.41 | 0.053 | 1.66 | 0.024 |
| 3220 | 1232.3 | 17.40% | 2597.9 | 19.00% | 2.108 | 0.012 | 1435.5 | 18.20% | 1.165 | 0.36 | 2200 | 10.10% | 1.79 | 0.01 | 532 | 6.40% | 0.43 | 0.005 | 913.4 | 10.70% | 0.74 | 0.079 | 1.72 | 0.003 |
| 3322 | 1240.5 | 13.20% | 1506.4 | 22.20% | 1.214 | 0.284 | 1676.3 | 1.00% | 1.351 | 0.01 | 1213.4 | 4.80% | 0.98 | 0.80 | 687.5 | 8.30% | 0.55 | 0.005 | 626.2 | 49.10% | 0.50 | 0.038 | 0.91 | 0.752 |
| 3324 | 815.1 | 14.80% | 2476.1 | 11.90% | 3.038 | 0.001 | 897.4 | 4.80% | 1.101 | 0.33 | 2101.8 | 19.10% | 2.58 | 0.01 | 1179.3 | 10.00% | 1.45 | 0.02 | 1989.1 | 44.60% | 2.44 | 0.086 | 1.69 | 0.192 |
| 3325 | 1507.9 | 5.10% | 1462.8 | 15.70% | 0.970 | 0.764 | 405.8 | 10.60% | 0.269 | 0.00 | 1549.5 | 2.40% | 1.03 | 0.44 | 1026.1 | 9.20% | 0.68 | 0.002 | 509.1 | 30.80% | 0.34 | 0.001 | 0.50 | 0.008 |
| 3327 | 705.1 | 22.60% | 1455 | 12.30% | 2.064 | 0.006 | 1060.2 | 7.00% | 1.504 | 0.02 | 973.6 | 21.40% | 1.38 | 0.15 | 820.1 | 15.90% | 1.16 | 0.388 | 588.5 | 7.70% | 0.83 | 0.29 | 0.72 | 0.044 |
| 3522 | 1433.9 | 4.60% | 443.5 | 20.80% | 0.309 | 0 | 447.9 | 23.30% | 0.312 | 0.00 | 273 | 8.10% | 0.19 | 0.00 | 1201.9 | 19.30% | 0.84 | 0.17 | 924.7 | 15.40% | 0.64 | 0.005 | 0.77 | 0.152 |
| 3719 | 131.2 | 10.40% | 738.3 | 3.70% | 5.627 | 0 | 158.8 | 4.20% | 1.210 | 0.03 | 483 | 15.70% | 3.68 | 0.00 | 128.8 | 10.10% | 0.98 | 0.841 | 597.6 | 46.30% | 4.55 | 0.043 | 4.64 | 0.043 |
| 4016 | 6123.1 | 10.40% | 12124.4 | 8.50% | 1.980 | 0.001 | 8677.2 | 12.50% | 1.417 | 0.02 | 12580.9 | 12.90% | 2.05 | 0.00 | 5000.1 | 9.70% | 0.82 | 0.072 | 11064.9 | 9.80% | 1.81 | 0.002 | 2.21 | 0.001 |
| 4026 | 5944.4 | 10.80% | 5078.3 | 6.10% | 0.854 | 0.103 | 2782.7 | 14.50% | 0.468 | 0.00 | 2090.6 | 8.50% | 0.35 | 0.00 | 7916.8 | 6.40% | 1.33 | 0.014 | 4971 | 31.70% | 0.84 | 0.377 | 0.63 | 0.037 |
| 4105 | 1006.1 | 4.10% | 689 | 20.10% | 0.685 | 0.019 | 463 | 16.60% | 0.460 | 0.00 | 279.8 | 7.40% | 0.28 | 0.00 | 320.4 | 14.70% | 0.32 | 0 | 269.5 | 65.80% | 0.27 | 0.002 | 0.84 | 0.656 |
| 4313 | 454.8 | 18.30% | 1187.7 | 7.50% | 2.611 | 0 | 682.4 | 22.00% | 1.500 | 0.08 | 1032.3 | 13.70% | 2.27 | 0.00 | 611.6 | 4.70% | 1.34 | 0.037 | 1042.1 | 15.20% | 2.29 | 0.005 | 1.70 | 0.01 |
| 4314 | 246 | 7.40% | 545.4 | 21.60% | 2.217 | 0.012 | 324.3 | 18.10% | 1.318 | 0.09 | 430.6 | 16.40% | 1.75 | 0.01 | 284.6 | 18.20% | 1.16 | 0.291 | 388.1 | 27.70% | 1.58 | 0.087 | 1.36 | 0.208 |
| 4517 | 797 | 4.00% | 1679.6 | 6.60% | 2.107 | 0 | 1218.5 | 18.80% | 1.529 | 0.03 | 748.3 | 18.00% | 0.94 | 0.57 | 950.9 | 12.60% | 1.19 | 0.097 | 701.6 | 23.30% | 0.88 | 0.377 | 0.74 | 0.1 |
| 4730 | 424.7 | 16.30% | 537 | 14.30% | 1.264 | 0.133 | 505.2 | 7.80% | 1.190 | 0.15 | 143.5 | 12.70% | 0.34 | 0.00 | 238.8 | 10.40% | 0.56 | 0.012 | 222.1 | 10.60% | 0.52 | 0.009 | 0.93 | 0.447 |
| 5223 | 4577.4 | 2.80% | 2175.6 | 19.20% | 0.475 | 0.001 | 1503.1 | 2.20% | 0.328 | 0.00 | 2268.7 | 13.00% | 0.50 | 0.00 | 1153.9 | 71.60% | 0.25 | 0.002 | 1391.8 | 35.90% | 0.30 | 0 | 1.21 | 0.692 |
| 5414 | 251.7 | 9.20% | 640.5 | 6.50% | 2.545 | 0 | 662.9 | 15.80% | 2.634 | 0.00 | 785.6 | 12.20% | 3.12 | 0.00 | 230.5 | 6.80% | 0.92 | 0.26 | 166.8 | 7.30% | 0.66 | 0.005 | 0.72 | 0.005 |
| 5521 | 3573.6 | 12.20% | 2297.2 | 2.40% | 0.643 | 0.007 | 2078.2 | 15.80% | 0.582 | 0.01 | 2569.8 | 12.00% | 0.72 | 0.03 | 4327.6 | 6.60% | 1.21 | 0.066 | 2857.8 | 11.20% | 0.80 | 0.084 | 0.66 | 0.004 |
| 5627 | 566.5 | 2.70% | 1036.4 | 19.10% | 1.829 | 0.015 | 1480.8 | 14.70% | 2.614 | 0.00 | 1374.6 | 14.60% | 2.43 | 0.00 | 860.1 | 14.10% | 1.52 | 0.014 | 2443.6 | 17.40% | 4.31 | 0.002 | 2.84 | 0.003 |
| 5840 | 224.2 | 7.40% | 580.1 | 15.90% | 2.587 | 0.003 | 410.7 | 18.90% | 1.832 | 0.02 | 313.8 | 8.90% | 1.40 | 0.01 | 349.6 | 21.90% | 1.56 | 0.05 | 547.9 | 4.60% | 2.44 | 0 | 1.57 | 0.013 |
| 5845 | 192.6 | 18.30% | 444.1 | 16.80% | 2.306 | 0.006 | 284.9 | 12.40% | 1.479 | 0.03 | 261.1 | 16.50% | 1.36 | 0.10 | 250.2 | 8.20% | 1.30 | 0.07 | 523.3 | 5.00% | 2.72 | 0 | 2.09 | 0 |
| 5846 | 263.5 | 17.60% | 373 | 19.90% | 1.416 | 0.096 | 423.2 | 0.80% | 1.606 | 0.00 | 283.1 | 10.30% | 1.07 | 0.57 | 172.5 | 50.40% | 0.65 | 0.185 | 242.2 | 94.40% | 0.92 | 0.882 | 1.40 | 0.648 |
| 5848 | 221.8 | 26.10% | 353 | 6.20% | 1.592 | 0.021 | 464.7 | 10.70% | 2.095 | 0.01 | 277.1 | 5.40% | 1.25 | 0.18 | 340 | 15.60% | 1.53 | 0.059 | 681.1 | 78.10% | 3.07 | 0.211 | 2.00 | 0.331 |
| 6016 | 1530.2 | 13.90% | 568.3 | 4.00% | 0.371 | 0.001 | 876.8 | 1.30% | 0.573 | 0.01 | 786.6 | 8.20% | 0.51 | 0.00 | 648.9 | 4.40% | 0.42 | 0.002 | 495.6 | 9.30% | 0.32 | 0.001 | 0.76 | 0.008 |
| 6018 | 1477.4 | 19.90% | 763.3 | 10.30% | 0.517 | 0.015 | 889.2 | 8.40% | 0.602 | 0.03 | 1043 | 11.10% | 0.71 | 0.08 | 336.3 | 13.20% | 0.23 | 0.003 | 418.9 | 15.90% | 0.28 | 0.004 | 1.25 | 0.149 |
| 6019 | 956.8 | 14.40% | 334.5 | 12.30% | 0.350 | 0.002 | 454.9 | 29.90% | 0.475 | 0.01 | 635.6 | 5.00% | 0.66 | 0.02 | 560.6 | 41.90% | 0.59 | 0.065 | 302.2 | 42.40% | 0.32 | 0.004 | 0.54 | 0.169 |
| 6218 | 2461.5 | 11.60% | 3229.9 | 6.20% | 1.312 | 0.019 | 2487.8 | 13.80% | 1.011 | 0.92 | 2285.5 | 16.40% | 0.93 | 0.55 | 2457.1 | 11.80% | 1.00 | 0.986 | 1053.7 | 56.40% | 0.43 | 0.021 | 0.43 | 0.021 |
| 6222 | 331.5 | 17.50% | 470.2 | 6.00% | 1.418 | 0.02 | 428.9 | 18.40% | 1.294 | 0.16 | 393.1 | 17.80% | 1.19 | 0.31 | 1140.1 | 15.90% | 3.44 | 0.002 | 743.5 | 65.50% | 2.24 | 0.219 | 0.65 | 0.257 |

| 6225 | 1567.4 | 10.80% | 1906.7 | 13.40% | 1.216 | 0.128 | 1526.1 | 14.10% | 0.974 | 0.81 | 1463.9 | 14.40% | 0.93 | 0.54 | 603.3 | 16.30% | 0.38 | 0.001 | 373.5 | 52.30% | 0.24 | 0.001 | 0.62 | 0.143 |
| --- | --- | --- | --- | --- | --- | --- | --- | --- | --- | --- | --- | --- | --- | --- | --- | --- | --- | --- | --- | --- | --- | --- | --- | --- |
| 6545 | 252.5 | 9.40% | 461 | 10.60% | 1.826 | 0.003 | 590.1 | 7.90% | 2.337 | 0.00 | 341.6 | 10.60% | 1.35 | 0.02 | 463.6 | 18.30% | 1.84 | 0.014 | 447.1 | 12.10% | 1.77 | 0.005 | 0.96 | 0.791 |
| 6834 | 271.9 | 7.30% | 506.9 | 11.80% | 1.864 | 0.003 | 549.1 | 28.90% | 2.019 | 0.04 | 368.5 | 8.80% | 1.36 | 0.01 | 583.1 | 7.20% | 2.14 | 0 | 671.9 | 23.50% | 2.47 | 0.012 | 1.15 | 0.4 |
| 6835 | 934.8 | 8.90% | 1057.3 | 19.20% | 1.131 | 0.389 | 1838 | 7.10% | 1.966 | 0.00 | 869.2 | 8.20% | 0.93 | 0.36 | 1173.6 | 6.40% | 1.26 | 0.021 | 1486.9 | 59.70% | 1.59 | 0.344 | 1.27 | 0.576 |
| 6905 | 209.5 | 8.40% | 371.5 | 4.30% | 1.773 | 0 | 515 | 2.50% | 2.458 | 0.00 | 280.4 | 9.70% | 1.34 | 0.02 | 303.2 | 1.80% | 1.45 | 0.001 | 473.3 | 18.90% | 2.26 | 0.007 | 1.56 | 0.03 |
| 6908 | 259.4 | 12.90% | 589.3 | 13.30% | 2.272 | 0.003 | 568.3 | 14.50% | 2.191 | 0.00 | 361.7 | 16.30% | 1.39 | 0.06 | 355.1 | 7.70% | 1.37 | 0.019 | 501.3 | 88.80% | 1.93 | 0.401 | 1.41 | 0.601 |
| 7012 | 2364.1 | 9.30% | 1684.3 | 17.20% | 0.712 | 0.032 | 2177.7 | 17.00% | 0.921 | 0.49 | 2349.6 | 21.30% | 0.99 | 0.97 | 1122.1 | 20.70% | 0.47 | 0.003 | 890.2 | 10.60% | 0.38 | 0 | 0.79 | 0.184 |
| 7220 | 397.7 | 6.60% | 989.9 | 18.80% | 2.489 | 0.006 | 640.8 | 17.40% | 1.611 | 0.02 | 770.4 | 3.70% | 1.94 | 0.00 | 255.5 | 12.50% | 0.64 | 0.004 | 278.8 | 19.00% | 0.70 | 0.025 | 1.09 | 0.552 |
| 7222 | 1017.7 | 5.70% | 1684.7 | 2.10% | 1.655 | 0 | 511.3 | 0.60% | 0.502 | 0.00 | 1385.3 | 7.90% | 1.36 | 0.01 | 910 | 16.40% | 0.89 | 0.31 | 1240.6 | 16.90% | 1.22 | 0.151 | 1.36 | 0.091 |
| 7226 | 1025.6 | 14.50% | 2240.5 | 7.50% | 2.185 | 0.001 | 978.4 | 1.50% | 0.954 | 0.61 | 2171.7 | 31.00% | 2.12 | 0.04 | 603.5 | 16.90% | 0.59 | 0.015 | 1014.6 | 52.10% | 0.99 | 0.974 | 1.68 | 0.257 |
| 7227 | 355.5 | 15.30% | 452 | 13.70% | 1.271 | 0.113 | 256.4 | 3.30% | 0.721 | 0.04 | 431.6 | 6.90% | 1.21 | 0.10 | 177.5 | 13.90% | 0.50 | 0.007 | 211.9 | 57.20% | 0.60 | 0.135 | 1.19 | 0.656 |
| 7711 | 6097.3 | 20.20% | 6236.9 | 10.00% | 1.023 | 0.869 | 7059.6 | 13.10% | 1.158 | 0.34 | 6176.7 | 7.70% | 1.01 | 0.92 | 2685.2 | 28.00% | 0.44 | 0.015 | 2472.8 | 12.90% | 0.41 | 0.008 | 0.92 | 0.675 |
| 7720 | 1773.2 | 8.40% | 4388.7 | 11.20% | 2.475 | 0.001 | 3989.7 | 11.40% | 2.250 | 0.00 | 3823.7 | 12.20% | 2.16 | 0.00 | 3127 | 10.90% | 1.76 | 0.003 | 2992.1 | 18.40% | 1.69 | 0.021 | 0.96 | 0.736 |
| 7728 | 745.5 | 9.10% | 925.2 | 12.50% | 1.241 | 0.081 | 1201.2 | 9.40% | 1.611 | 0.00 | 747.9 | 5.10% | 1.00 | 0.96 | 685 | 50.20% | 0.92 | 0.78 | 349.8 | 20.10% | 0.47 | 0.002 | 0.51 | 0.174 |
| 8115 | 842.6 | 6.10% | 1391.8 | 21.90% | 1.652 | 0.037 | 496.2 | 3.80% | 0.589 | 0.00 | 1214.3 | 9.90% | 1.44 | 0.01 | 547.9 | 14.90% | 0.65 | 0.006 | 1379.7 | 13.30% | 1.64 | 0.008 | 2.52 | 0.002 |
| 8116 | 139.9 | 21.90% | 319.4 | 16.70% | 2.283 | 0.007 | 164.9 | 3.40% | 1.179 | 0.24 | 299.9 | 20.00% | 2.14 | 0.01 | 218.2 | 11.90% | 1.56 | 0.028 | 149.1 | 14.40% | 1.07 | 0.693 | 0.68 | 0.024 |
| 8123 | 571.8 | 5.80% | 543.3 | 10.40% | 0.950 | 0.494 | 556.8 | 2.10% | 0.974 | 0.50 | 521.4 | 8.20% | 0.91 | 0.18 | 328.9 | 9.90% | 0.58 | 0.001 | 237.7 | 4.80% | 0.42 | 0 | 0.72 | 0.01 |
| 8232 | 533.8 | 18.30% | 1122.7 | 3.80% | 2.103 | 0.001 | 704.6 | 13.30% | 1.320 | 0.09 | 1160.1 | 4.70% | 2.17 | 0.00 | 177.2 | 3.60% | 0.33 | 0.003 | 414 | 6.80% | 0.78 | 0.11 | 2.34 | 0 |
| 8319 | 354.4 | 6.40% | 650 | 8.10% | 1.834 | 0.001 | 711.6 | 8.60% | 2.008 | 0.00 | 357.5 | 8.90% | 1.01 | 0.90 | 732.5 | 12.50% | 2.07 | 0.002 | 511.9 | 10.80% | 1.44 | 0.01 | 0.70 | 0.023 |
| 8327 | 389.5 | 12.80% | 411.1 | 2.80% | 1.055 | 0.508 | 784 | 19.00% | 2.013 | 0.01 | 611.5 | 15.90% | 1.57 | 0.02 | 384.3 | 14.70% | 0.99 | 0.91 | 465.2 | 2.30% | 1.19 | 0.063 | 1.21 | 0.071 |
| 8530 | 350.7 | 27.90% | 494.4 | 9.80% | 1.410 | 0.085 | 720.3 | 20.30% | 2.054 | 0.02 | 428.2 | 17.00% | 1.22 | 0.33 | 489.2 | 14.30% | 1.39 | 0.117 | 408.5 | 14.40% | 1.16 | 0.43 | 0.84 | 0.201 |
| 8606 | 420 | 19.50% | 641.5 | 16.30% | 1.527 | 0.045 | 731.5 | 13.10% | 1.742 | 0.01 | 735.3 | 15.10% | 1.75 | 0.02 | 299.1 | 14.40% | 0.71 | 0.086 | 364.7 | 17.40% | 0.87 | 0.407 | 1.22 | 0.213 |
| 8608 | 340 | 6.70% | 612.6 | 16.90% | 1.802 | 0.011 | 719.2 | 1.50% | 2.115 | 0.00 | 496.6 | 6.30% | 1.46 | 0.00 | 625.2 | 18.40% | 1.84 | 0.013 | 351 | 5.10% | 1.03 | 0.546 | 0.56 | 0.015 |
| 8726 | 201.5 | 9.10% | 247 | 12.60% | 1.226 | 0.094 | 268.3 | 3.30% | 1.332 | 0.00 | 263.2 | 6.50% | 1.31 | 0.01 | 170 | 9.90% | 0.84 | 0.094 | 122.1 | 8.00% | 0.61 | 0.003 | 0.72 | 0.013 |
| 8728 | 446.7 | 9.90% | 694.6 | 5.00% | 1.555 | 0.002 | 897.5 | 11.00% | 2.009 | 0.00 | 542.3 | 6.70% | 1.21 | 0.04 | 725.2 | 17.80% | 1.62 | 0.024 | 390.9 | 9.40% | 0.88 | 0.169 | 0.54 | 0.013 |
| 8732 | 811.5 | 18.00% | 1060.3 | 20.10% | 1.307 | 0.171 | 1100.2 | 2.80% | 1.356 | 0.03 | 1107.9 | 5.40% | 1.37 | 0.03 | 478.9 | 13.00% | 0.59 | 0.022 | 438.3 | 39.60% | 0.54 | 0.047 | 0.92 | 0.723 |
| 9117 | 850.1 | 14.60% | 264.9 | 30.90% | 0.312 | 0.002 | 1085.4 | 42.10% | 1.277 | 0.44 | 548.4 | 4.60% | 0.65 | 0.01 | 769.7 | 36.10% | 0.91 | 0.671 | 776.3 | 78.80% | 0.91 | 0.848 | 1.01 | 0.987 |
| 9118 | 1025.6 | 7.50% | 1323 | 2.00% | 1.290 | 0.003 | 1139.6 | 31.20% | 1.111 | 0.62 | 1137.9 | 26.50% | 1.11 | 0.57 | 261.1 | 20.40% | 0.25 | 0 | 206.5 | 12.40% | 0.20 | 0 | 0.79 | 0.185 |
| 9221 | 1461.4 | 16.40% | 257 | 18.30% | 0.176 | 0.001 | 1548.7 | 9.00% | 1.060 | 0.62 | 332 | 19.10% | 0.23 | 0.00 | 1849.7 | 8.20% | 1.27 | 0.077 | 238.6 | 9.20% | 0.16 | 0.001 | 0.13 | 0 |
| 9227 | 594.6 | 5.50% | 756.8 | 10.20% | 1.273 | 0.028 | 1100.4 | 15.50% | 1.851 | 0.01 | 755.6 | 38.00% | 1.27 | 0.39 | 484.2 | 38.60% | 0.81 | 0.37 | 584 | 57.70% | 0.98 | 0.96 | 1.21 | 0.677 |
| 9313 | 626.7 | 34.80% | 1304.2 | 5.30% | 2.081 | 0.007 | 1368.8 | 19.80% | 2.184 | 0.02 | 820.6 | 30.10% | 1.31 | 0.37 | 503 | 30.60% | 0.80 | 0.467 | 682.5 | 16.50% | 1.09 | 0.714 | 1.36 | 0.178 |
| 9314 | 452.2 | 45.80% | 543.3 | 14.30% | 1.201 | 0.515 | 1232.4 | 12.50% | 2.725 | 0.01 | 837.2 | 57.60% | 1.85 | 0.27 | 269.4 | 46.30% | 0.60 | 0.261 | 316 | 64.50% | 0.70 | 0.462 | 1.17 | 0.752 |
| 9603 | 429.4 | 14.70% | 826.6 | 13.10% | 1.925 | 0.005 | 1010.4 | 13.50% | 2.353 | 0.00 | 809.4 | 15.20% | 1.88 | 0.01 | 438.8 | 4.10% | 1.02 | 0.816 | 362.7 | 34.70% | 0.84 | 0.458 | 0.83 | 0.359 |
| 9708 | 168.9 | 15.20% | 399.2 | 11.80% | 2.364 | 0.002 | 462.8 | 19.80% | 2.740 | 0.01 | 386.8 | 15.60% | 2.29 | 0.00 | 300.4 | 15.20% | 1.78 | 0.012 | 190.7 | 38.30% | 1.13 | 0.651 | 0.63 | 0.092 |

Spot N°, spot label on the reference 2DE-map presented in **Figure S1**;

Control Mean, average of normalized spot volumes of untreated apple fruit as obtained from densitometric analysis of individual spots from proteins in 2D gels stained with silver nitrate from three different gels and independent extractions

Control Coef. Var %, Coefficient of variation of normalized spot volumes of untreated fruit as obtained from densitometric analysis of individual spots from proteins in 2D gels stained with silver nitrate from three different gels and independent extractions

Control-Bc Mean, average of normalized spot volumes of untreated fruit inoculated with *B. cinerea*, as obtained from densitometric analysis of individual spots from proteins in 2D gels stained with silver nitrate from three different gels and independent extractions

Control-Bc Coef. Var %, Coefficient of variation of normalized spot volumes of untreated apple fruit inoculated with *B. cinerea* as obtained from densitometric analysis of individual spots from proteins in 2D gels stained with silver nitrate from three different gels and independent extractions

Ratio Control-Bc/Control Mean, The Control-Bc (Mean) spot volume divided by the Control (Mean) spot volume

P value Control-Bc/Control, p value between normalized spot volumes of individual spots from three different gels and independent extractions of untreated apple fruit and normalized spot volumes of individual spots from three different gels and independent extractions of apple fruit inoculated with *B. cinerea*, as obtained from densitometric analysis of individual spots from proteins in 2D gels stained with silver nitrate from three different gels and independent extractions

1-MCP-Bc Mean, average of normalized spot volumes of 1-MCP treated apple fruit inoculated with *B. cinerea* as obtained from densitometric analysis of individual spots from proteins in 2D gels stained with silver nitrate from three different gels and independent extractions

1-MCP-Bc Coef. Var %, Coefficient of variation of normalized spot volumes of 1-MCP treated apple fruit inoculated with *B. cinerea* as obtained from densitometric analysis of individual spots from proteins in 2D gels stained with silver nitrate from three different gels and independent extractions

Ratio 1-MCP-Bc Mean/Control Mean, the 1-MCP-Bc (Mean) spot volume divided by the Control (Mean) spot volume

P value 1-MCP-Bc / Control, p value between normalized spot volumes of individual spots from three different gels and independent extractions of inoculated 1-MCP treated apple fruit and normalized spot volumes of individual spots from three different gels and independent extractions of untreated apple fruit as obtained from densitometric analysis of individual spots from proteins in 2D gels stained with silver nitrate from three different gels and independent extractions

O_3_-Bc Mean, average of normalized spot volumes of O_3_ treated apple fruit inoculated with *B. cinerea* as obtained from densitometric analysis of individual spots from proteins in 2D gels stained with silver nitrate from three different gels and independent extractions

O_3_-Bc Coef. Var %, Coefficient of variation of normalized spot volumes of O_3_ treated apple fruit inoculated with *B. cinerea* as obtained from densitometric analysis of individual spots from proteins in 2D gels stained with silver nitrate from three different gels and independent extractions

Ratio O_3_-Bc Mean/Control Mean, the O_3_-Bc (Mean) spot volume divided by the Control (Mean) spot volume

P value O_3_-Bc / Control, p value between normalized spot volumes of individual spots from three different gels and independent extractions of inoculated O_3_ treated apple fruit and normalized spot volumes of individual spots from three different gels and independent extractions of untreated apple fruit as obtained from densitometric analysis of individual spots from proteins in 2D gels stained with silver nitrate from three different gels and independent extractions

1-MCP Mean, average of normalized spot volumes of 1-MCP apple fruit as obtained from densitometric analysis of individual spots from proteins in 2D gels stained with silver nitrate from three different gels and independent extractions

1-MCP Coef. Var %, Coefficient of variation of normalized spot volumes of 1-MCP treated fruit as obtained from densitometric analysis of individual spots from proteins in 2D gels stained with silver nitrate from three different gels and independent extractions

Ratio 1-MCP Mean/Control Mean, the 1-MCP (Mean) spot volume divided by the Control (Mean) spot volume

P value 1-MCP/Control, p value between normalized spot volumes of individual spots from three different gels and independent extractions of 1-MCP treated apple fruit and normalized spot volumes of individual spots from three different gels and independent extractions of untreated apple fruit as obtained from densitometric analysis of individual spots from proteins in 2D gels stained with silver nitrate from three different gels and independent extractions

O_3_ Mean, average of normalized spot volumes of O_3_ apple fruit as obtained from densitometric analysis of individual spots from proteins in 2D gels stained with silver nitrate from three different gels and independent extractions

O_3_ Coef. Var %, Coefficient of variation of normalized spot volumes of O_3_ treated fruit as obtained from densitometric analysis of individual spots from proteins in 2D gels stained with silver nitrate from three different gels and independent extractions

Ratio O_3_ Mean/Control Mean, the O_3_ (Mean) spot volume divided by the Control (Mean) spot volume

P value O_3_ /Control, p value between normalized spot volumes of individual spots from three different gels and independent extractions of O_3_ treated apple fruit and normalized spot volumes of individual spots from three different gels and independent extractions of untreated apple fruit as obtained from densitometric analysis of individual spots from proteins in 2D gels stained with silver nitrate from three different gels and independent extractions

Ratio O_3_ Mean/1-MCP Mean, The O_3_ (Mean) spot volume divided by the 1-MCP (Mean) spot volume

P value O_3_/1-MCP, p value between normalized spot volumes of individual spots from three different gels and independent extractions of O_3_ treated apple fruit and normalized spot volumes of individual spots from three different gels and independent extractions of 1-MCP treated apple fruit as obtained from densitometric analysis of individual spots from proteins in 2D gels stained with silver nitrate from three different gels and independent extractions


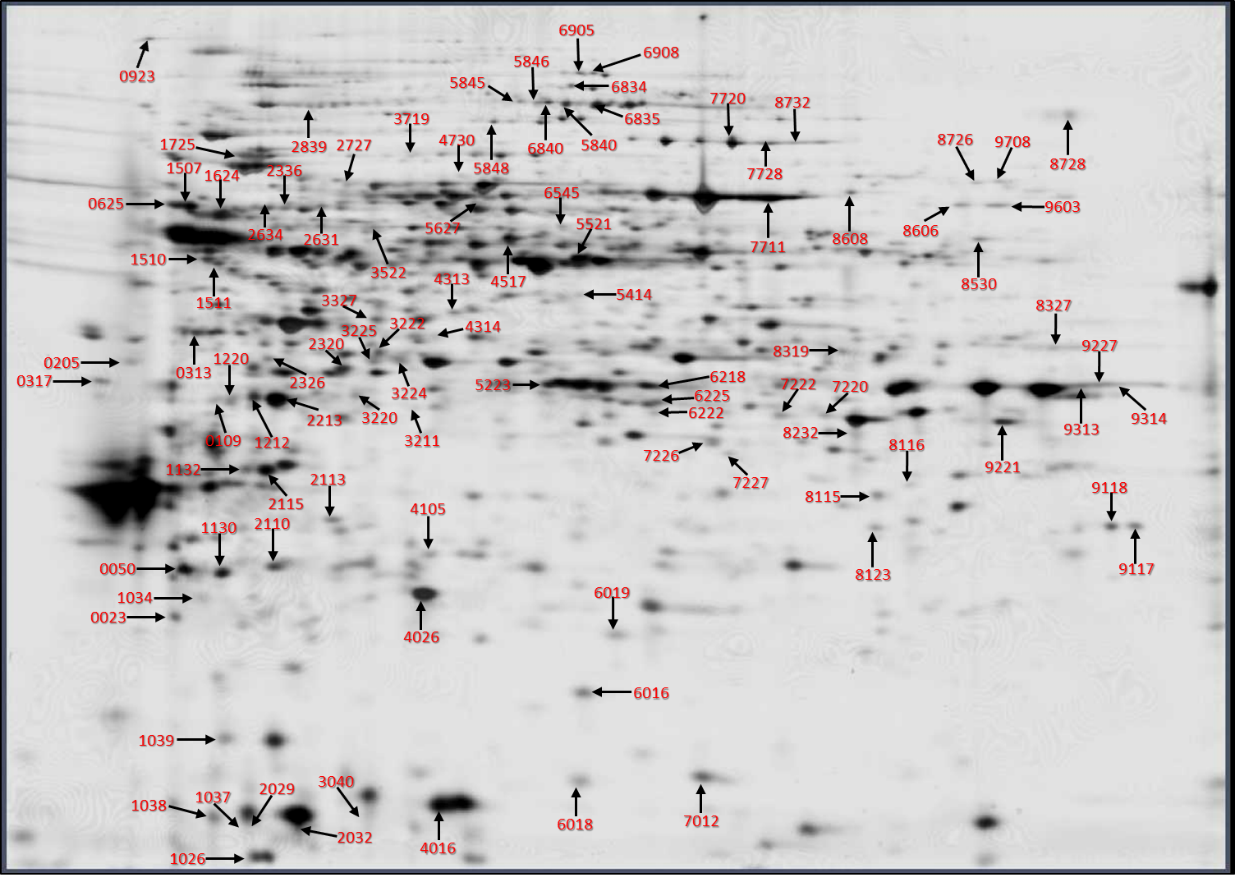


Figure S1. Reference map for apple fruit proteins. The specific proteins spots labelled with the arrows correspond to proteins listed in the Supplementary Data **Table S1A & B**.


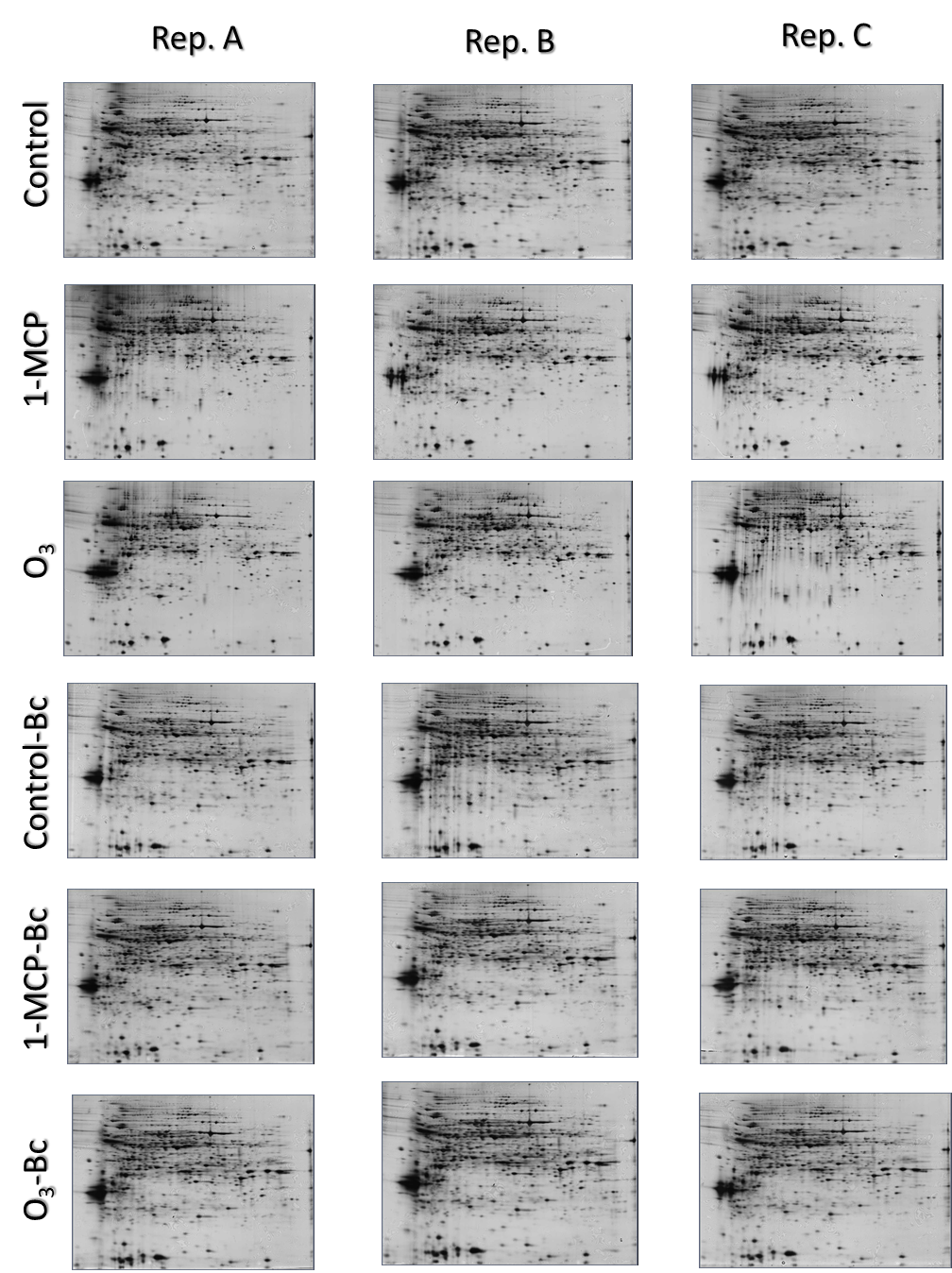


Figure S2. Full-length gels of apple fruit proteins of non-inoculated (‘Control,’ ‘1-MCP’ and ‘O_3_’)/or inoculated with *B. cinerea* pathogen (‘Control-Bc’, 1-MCP-Bc’ and ‘O3-Bc’) of untreated or 1-MCP and O_3_ treated apple fruit in which zoomed in views of selected areas are presented in **Figure 3**.
